# MBNL depletion drives stem cell fusion and immature myonuclear states in myotonic dystrophy type 1

**DOI:** 10.1038/s41467-026-76476-6

**Published:** 2026-08-06

**Authors:** Vanessa Todorow, Xavière Lornage, Shinichiro Hayashi, Fiorella Carla Grandi, Zoé Clerc, Jeanne Lainé, Michel Ney, Mégane Lemaitre, Corentin Rouxel, Maria Kondili, Piera Smeriglio, Hanseul Oh, Mie Kato, Nobuyuki Eura, Yoshihiko Saito, Francia Victoria A. De Los Reyes, Satoru Noguchi, Benedikt Schoser, Denis Furling, Ichizo Nishino, Peter Meinke, Frédérique Rau

**Affiliations:** 1 Friedrich-Baur-Institute at the Department of Neurology, LMU University Hospital, LMU Medizin, Ludwig-Maximilians-Universität München, Munich, Germany; 2 Department of Neuromuscular Research, National Institute of Neuroscience, National Center of Neurology and Psychiatry, Tokyo, Japan; 3 Sorbonne Université, Inserm, Institut de Myologie, Centre de Recherche en Myologie, Paris, France; 4 Sorbonne Université, Inserm, Phénotypage du Petit Animal, Paris, France; 5 Department of Neurology, Nara Medical University, Nara, Japan

**Keywords:** Mechanisms of disease, Regeneration, Skeletal muscle, Transcriptomics, Neuromuscular disease

## Abstract

Myotonic dystrophy type 1 is caused by the expression of expanded CTG repeats in the *DMPK* gene and the resulting loss of function of MBNL protein. Affected skeletal muscle displays abundant centrally located nuclei despite limited immune-cell–associated fibre necrosis, complicating interpretation of muscle damage and remodelling mechanisms. Here we show that muscle stem cells are activated and fuse with existing muscle fibres in myotonic dystrophy type 1. Single-nucleus transcriptomics in patient’s muscle identifies increased activated muscle stem cells and distinct myonuclear populations exhibiting transitional transcriptional states, including muscle stem cell associated markers and elevated *DMPK* expression. Myofibre-specific MBNL knockdown mouse models demonstrate that muscle stem cell fusion contributes to central nucleation, whereas their deletion does not improve myotonia or muscle strength. Together, these findings indicate that loss of MBNL function in muscle drives myonuclear accretion through stem cell-mediated fusion, giving rise to myonuclei with immature states in myotonic dystrophy type 1.

## Abstract (web summary)

This study shows that muscle stem cells fuse with existing muscle fibres in myotonic dystrophy type 1, producing centrally located nuclei with altered gene-expression states and reshaping diseased muscle.

## Introduction

Myotonic dystrophy type 1 (DM1), the most common form of adult-onset muscular dystrophy, is caused by an unstable expansion of a CTG repeat located in the 3’ untranslated region of the *DMPK* gene^1–3^. Individuals affected by this condition often demonstrate delayed muscle relaxation (myotonia), progressive muscle weakness and wasting, cardiac conduction defects, gastrointestinal dysmotility, early-onset cataracts, endocrine abnormalities, and cognitive deficits^4,5^. This inherited disorder is characterised by a multisystemic clinical presentation, and patients exhibit high variability in muscular and extra-muscular symptoms, spanning a continuum from mild to severe^6–8^. Nevertheless, clinical symptom severity correlates with the size of the CTG expansion and earlier disease onset^9,10^.

DM1 is an RNA-dominant disease triggered by the expression of pathogenic *DMPK* transcripts containing expanded CUG repeats (CUGexp). These CUGexp-RNAs are retained in the nucleus as ribonucleoprotein aggregates known as foci^11,12^ and disrupt the function of RNA-binding proteins, particularly those of the muscleblind-like protein (MBNL) family (also known as muscleblind-like splicing regulators), leading to widespread alterations in RNA metabolism, including alternative splicing, mRNA stability, polyadenylation, and miRNA processing^13–16^. Indeed, MBNL proteins bind with high affinity to YGCY motifs (Y = pyrimidine) and, therefore, to expanded CUG repeats in proportion to the size of this expansion; their sequestration within nuclear CUGexp-foci results in a loss of MBNL activity^17^ and concomitant splicing misregulation that mirrors embryonic splicing patterns^18^. Beyond sequestration, additional mechanisms lower effective MBNL1 levels in DM1. Patient-derived myogenic models show repeat-dependent dysregulation of MBNL1/2 splicing, with increased inclusion of specific exons and imbalanced isoform pools that reduce functional protein and alter nuclear-cytoplasmic partitioning already at the myoblast stage^19^. These data indicate that MBNL insufficiency starts upstream of mature fibres. Moreover, microRNA-mediated repression of MBNL1—most notably by miR-23b and miR-218, whose levels correlate with CTG repeat size—actively suppresses MBNL1 protein; antimiR treatment both restores MBNL1 and improves DM1 molecular and cellular phenotypes^20^. Together, these findings support a multi-hit model in which sequestration, isoform/partitioning changes, and active post-transcriptional repression converge to deplete functional MBNL1 in DM1. These abnormalities contribute to several core symptoms of DM1, including myotonia, muscle weakness, and cardiac dysfunction^21–25^. Moreover, mouse models with genetic knockout of Mbnl1 alone or in combination with Mbnl2 recapitulate phenotypes reminiscent of DM1, further supporting the key role of MBNL loss of function in DM1 pathogenesis^26–28^.

Skeletal muscle from DM1 patients exhibits characteristics of a slow progressive dystrophic myopathology. In dystrophic muscle, myofibre damage can arise through distinct processes with different cellular consequences. Myofibre necrosis involves immune-cell infiltration, sarcolemmal rupture, or a metabolic breakdown as seen in rhabdomyolysis, and ends in irreversible fibre loss. This triggers canonical regeneration through de novo myofibre formation mediated by muscle stem cells (MuSCs), also referred to as satellite cells. In contrast, sublethal damage preserves sarcolemmal integrity, lacks immune infiltration, and results in structural alterations of surviving fibres, reflecting adaptive or maladaptive remodelling rather than fibre replacement. DM1 muscle presents a distinctive combination of these features. As in other muscular dystrophies, centrally located myonuclei are abundant; however, their prevalence in DM1 is unusually high despite the relative paucity of overt necrotic myofibre degeneration and infiltration. DM1 muscles instead display pronounced fibre size variability, fibre-type-specific atrophy and hypertrophy, fibre splitting, ringed fibres, and progressive fibrosis and fat replacement, ultimately leading to muscle weakness and wasting^4,5^.

The coexistence of extensive central nucleation with minimal immune-cell-associated fibre necrosis complicates interpretation of the underlying damage and repair processes, raising the question of whether canonical degeneration–regeneration cycles or alternative, homeostatic remodelling mechanisms predominate in DM1 muscle. In adults, maintenance of skeletal muscle homeostasis critically depends on resident MuSCs^29^. Under homeostatic conditions, MuSCs are quiescent but become activated in response to injury, inflammation, damage, and to mechanical stress^30^. Furthermore, a subset of MuSCs self-renews to maintain the stem cell pool^31^. Previous in vitro studies have shown that primary myoblasts derived from DM1 patient MuSCs display reduced proliferative and differentiation capacity and premature cellular senescence, a condition also observed in situ in patient muscle specimens^32–38^. Although these results suggest that the altered myogenic potential of MuSCs may contribute to DM1 muscle pathogenesis, their activation and behaviour in vivo have not been examined in adult disease muscles.

To address this question, we selectively depleted *Mbnl1* and *Mbnl2* in post-mitotic myofibres of adult mice, thereby avoiding developmental confounders. MBNL-knockdown muscles recapitulated key molecular, physiological, and histological features of DM1, including central nucleation accompanied by an increased number of peripheral myonuclei. We demonstrate that myofibre-restricted MBNL depletion triggers MuSC activation, proliferation, and subsequent fusion into existing myofibres, resulting in both central and peripheral nuclear positioning. Single-nucleus RNA-sequencing (snRNA-seq) revealed the emergence of transcriptionally distinct myonuclear populations with features of incomplete maturation.

In parallel, snRNA-seq analysis of human DM1 muscle biopsies identified a transcriptionally distinct cluster characterised by transitional gene expression signatures and elevated *DMPK* levels. Consistent with the mouse model, DM1 muscles exhibited increased numbers of MuSCs, including activated cells, distributed throughout the tissue in the absence of overt necrotic degeneration or immune-cell infiltration. Together, these findings indicate that MuSC fusion contributes to myonuclear heterogeneity in DM1 muscle and gives rise to centrally located myonuclei with altered transcriptional states and high expression of the toxic *DMPK* transcript.

## Results

### *Mbnl* knockdown in adult myofibres recapitulates adult human DM1 muscle features

MBNL loss of function plays a pivotal role in DM1 pathogenesis^18^. To assess the specific consequences of MBNL protein depletion in adult skeletal muscle, we developed an AAV-based mouse model using a specific Pol II-miRNA interference system to silence *Mbnl1* and *Mbnl2* exclusively within myofibres. In this model, *tibialis anterior* (TA) muscles of adult 2-month-old wild-type mice were injected with AAV2/9 vectors expressing Mbnl-shmiR under the control of the muscle-specific Spc5.12 promoter^39–42^ (Mbnl knockdown or KD). In contrast, contralateral TA muscles received a control AAV2/9 expressing a non-targeting shmiR (Ctrl). Quantitative analyses confirmed an 80% reduction in *Mbnl1* and approximately 50% reduction in *Mbnl2* transcript levels in Mbnl KD muscles compared to controls (Supplementary Fig. 1a, b), validating the efficacy of the silencing strategy. To assess whether these changes were reflected at the protein level, we performed Western blot analysis of MBNL1 and MBNL2. Consistent with the transcript data, MBNL1 protein levels were decreased by approximately 50% in Mbnl KD muscles. In contrast, MBNL2 protein abundance was increased (Supplementary Fig. 1c–e). RNA-seq analysis revealed abnormal inclusion of Mbnl2 exon 9, a splicing event regulated by MBNL1. Inclusion of this exon is known to stabilise the MBNL2+ex9 isoform^43^, likely contributing to the elevated MBNL2 protein levels (Supplementary Fig. 1f) as previously described^43^.

**Fig. 1:**
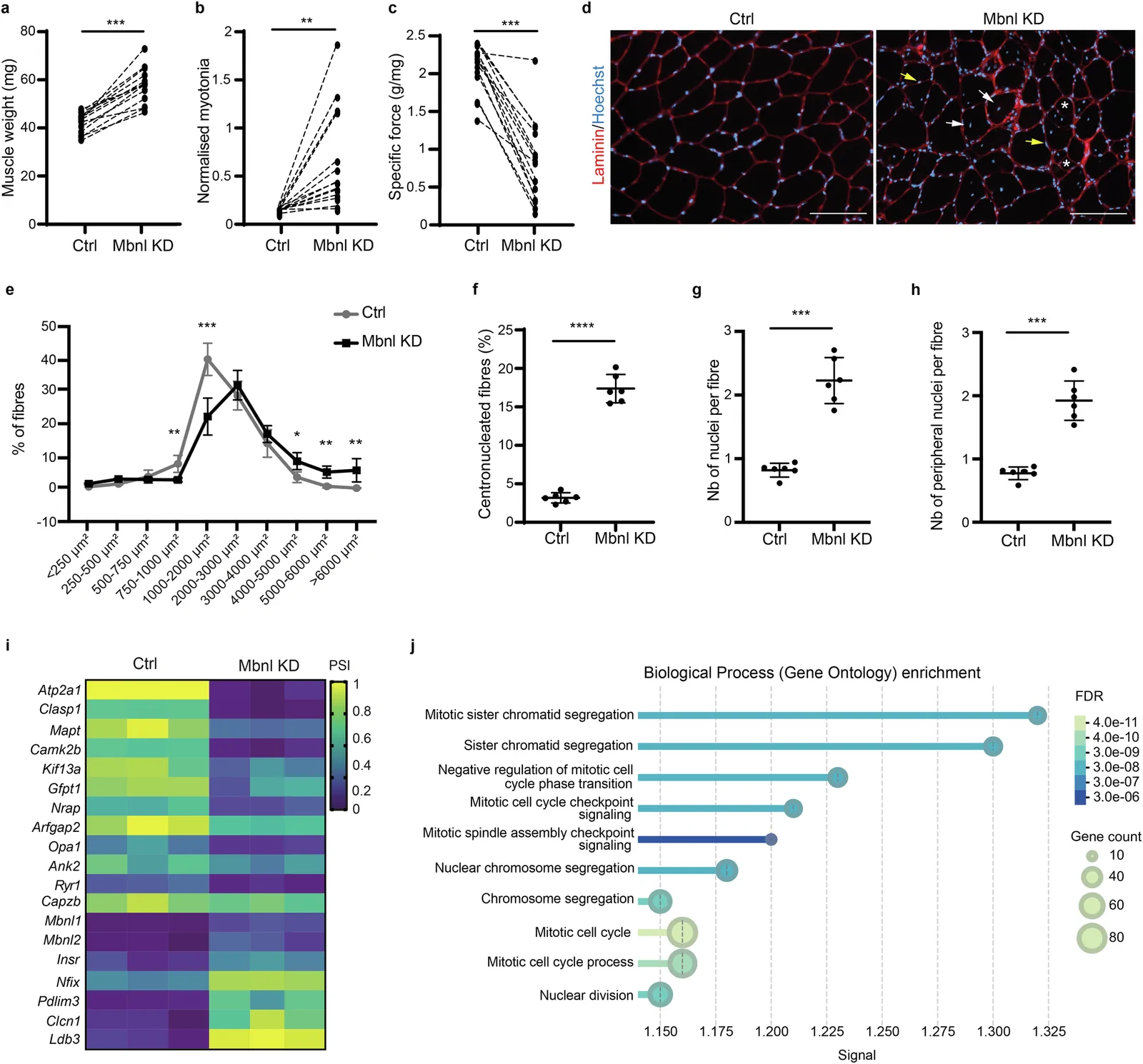
Functional and histological impact of Mbnl knockdown in adult skeletal myofibres. **a** Comparison of TA muscle weight across groups (Mbnl KD vs. Ctrl). **b** Myotonia. **c** Specific force corresponding to maximal force normalised to muscle weight (g/mg). **d** Transverse muscle sections stained with Hoechst (blue) and laminin (red), highlighting histological abnormalities induced by AAV-shMbnl injections (Mbnl-KD). Asterisks (*) denote split fibres; white arrows indicate central nuclei; yellow arrows indicate peripheral nuclei. Scale bars: 100 μm. **e** Myofibre size distribution across different categories (μm^2^). **f** Percentage of muscle fibres with central nuclei in transverse sections. **g** Number of nuclei per myofibre in transverse sections. **h** Number of peripheral nuclei per myofibre in transverse sections. **i** Heatmap showing selected differentially expressed splicing (DES) events in Mbnl KD TA muscles compared with Ctrl TA muscles. **j** Pathway analysis of upregulated genes in Mbnl KD TA compared with Ctrl TA based on RNA-seq data. Statistical analysis: **a**–**c**: *n* = 14 mice; **e**–**h**: *n* = 6 mice; **i**, **j**: *n* = 3 mice were used; Ctrl and Mbnl KD muscles were contralateral muscles from the same animals. For the quantification of transversal muscle sections, at least 1500 fibres were analysed. Data are presented as mean ± SD. *p < 0.05, **p < 0.01, ***p < 0.001, ****p < 0.0001; two-sided paired *t*-tests (**a**–**c**, **f**–**h**) and two-way ANOVA followed by Šídák’s multiple-comparisons test (**e**). Exact *p* values are provided in the Source data file.

Ten weeks post-injection, Mbnl KD TA muscles displayed DM1-like features, including myotonia and reduced specific tetanic strength compared to contralateral control TA muscles, accompanied by increased muscle mass (Fig. 1a–c). Histopathological examination revealed overt dystrophic changes, including fibre splitting and fibre size heterogeneity with an increase of hypertrophic fibres (Fig. 1d, e; Supplementary Fig. 1g). However, the total number of myofibres was not significantly altered in Mbnl-knockdown TA muscles (Supplementary Fig. 1h). A shift toward oxidative fibre types was observed in Mbnl-depleted TA muscles, with type IIa fibres increasing from 15 to 62% and type IIb fibres decreasing from 50 to 25%; these features were absent in contralateral control muscles (Supplementary Fig. 1i–k). Importantly, these histological changes occurred in the absence of major myofibre degeneration or necrosis, consistent with previous reports in MBNL KO mice^26^. Additionally, a two-fold increase in the total number of nuclei per fibre was observed, mainly associated with peripheral nuclei. Moreover, 18% of myofibres present centrally located nuclei (Fig. 1f–h). Analysis of the relationship between myofibre size and myonuclear number (both centralised and peripheral) further revealed that fibres containing centralised nuclei had cross-sectional areas ranging from 250 to 5000 µm², whereas peripheral myonuclei were more frequent in larger fibres (above 4000 µm²; Supplementary Fig. 2a, b). Moreover, fibres containing one or two centralised nuclei were significantly smaller than the average of all myofibres, while those with three or more centralised nuclei exhibited sizes comparable to the mean of total myofibres (Supplementary Fig. 2c).

Altogether, these results recapitulate key histopathological hallmarks of DM1, including central nucleation, fibre-type transition, and increased myonuclear number. Importantly, the broad distribution of centrally nucleated fibres across myofibre sizes, together with the unchanged total myofibre number, argues against canonical degeneration–regeneration cycles and instead supports a model in which MBNL depletion drives myofibre remodelling and myonuclear accretion in the absence of overt degenerative pathology.

To determine whether the molecular features of DM1 were recapitulated in this model, we performed bulk RNA-sequencing on Mbnl KD and control TA muscles. Analysis revealed widespread mis-splicing events previously associated with DM1 and known to be MBNL-regulated, notably the inclusion of exon 22 in *Atp2a1* and exon 7a in *Clcn1* (Fig. 1i; Δ*Ψ* > 0.2, FDR < 0.05; Supplementary Data 1). The presence of these specific splicing defects further confirmed that MBNL silencing in adult muscle reproduces key molecular signatures of DM1.

Gene Ontology (GO) enrichment analysis of upregulated genes in Mbnl KD muscles (Log2FC > 1; FDR < 0.05; Supplementary Data 2) highlighted pathways related to mitosis and cell division (Fig. 1j), and revealed increased expression of multiple growth factors (*Hgf*, *Fgf6*, *Areg*, *Gdf11*, *Gdf15*), concomitant with upregulation of extracellular matrix components and remodelling enzymes (*Col1a1*, *Fn1*, *Mmp3*, *Postn*; Supplementary Data 2). Together, these changes reflect a shift in the muscle microenvironment that is favourable for MuSCs activation, and suggest that MBNL depletion in post-mitotic fibres promotes the activation and proliferation of MuSCs and/or other cell types present in the muscle.

**Fig. 2:**
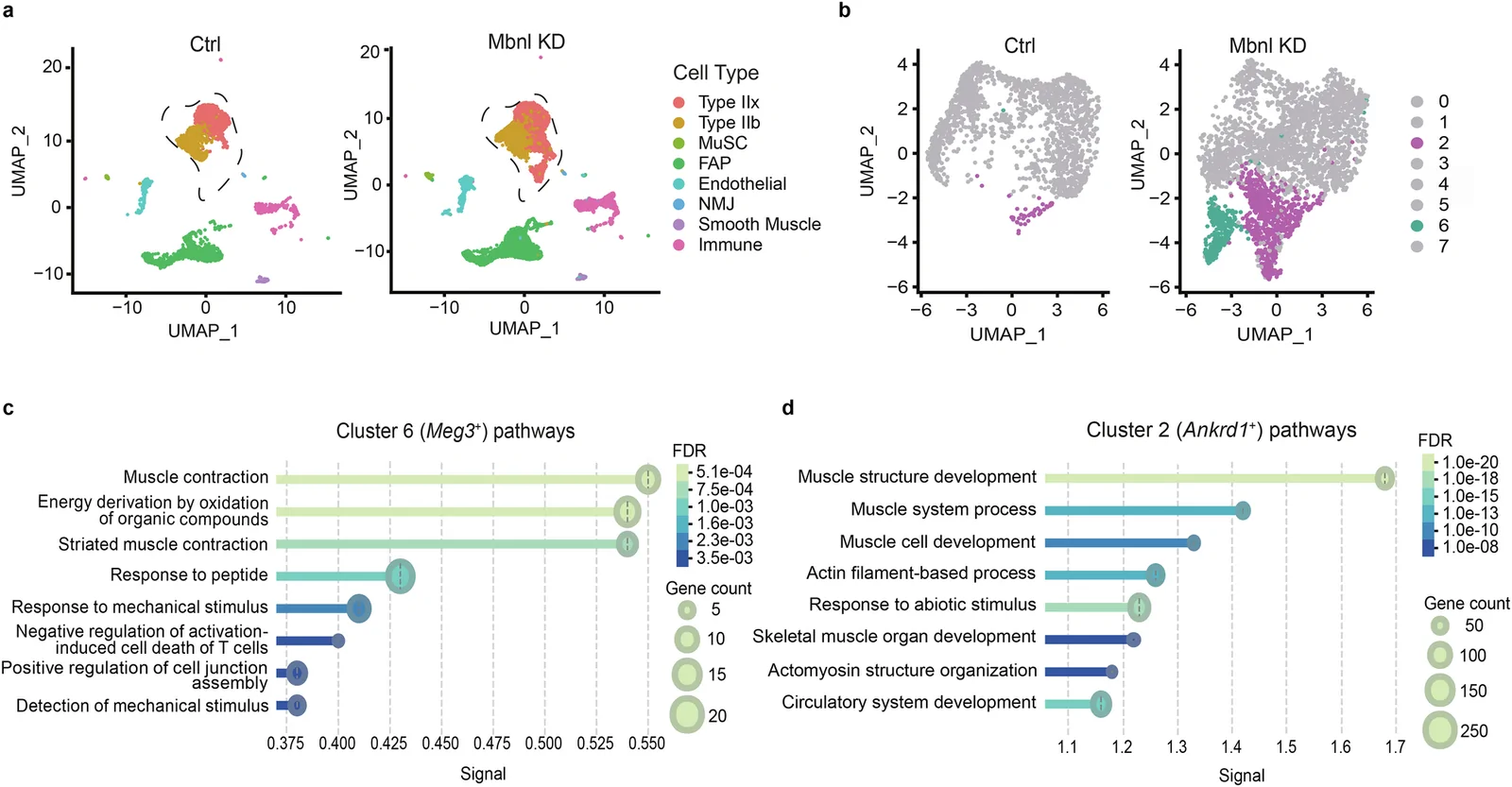
snRNA-seq analysis reveals two distinct myonuclear clusters in Mbnl knockdown myofibres. **a** Uniform manifold approximation and projection (UMAP) plot of nuclei from Ctrl and Mbnl KD TA muscles. Each point represents a single captured nucleus, whose position on the graph is determined by the ensemble of all detected genes. The colours identify different nuclear populations derived from clustering analysis. **b** Subclustering of all myonuclei from Ctrl and Mbnl KD TA muscles. The enriched novel populations in the KD muscle are highlighted in teal and purple, respectively. **c** Pathway analysis for the genes enriched (upregulated) in the *Meg3*^+^ cluster. **d** Pathway analysis for the genes enriched (upregulated) in the *Ankrd1*^+^ cluster (*n* = 2 TA muscles per condition).

To refine our understanding of the cellular dynamics in Mbnl KD TA muscles, we performed single-nucleus RNA-sequencing (snRNA-seq) 10 weeks after AAV-mediated Mbnl knockdown (Fig. 2a; Supplementary Fig. 3; Supplementary Data 3). Analysis of transcriptional profiles revealed a global reduction in *Mbnl1* expression across myonuclear populations, without evidence for a distinct subpopulation retaining control-like expression levels—in contrast to other cell types—and increased expression of canonical myoblast activation markers, including *Myog* and *Myod1*, in Mbnl KD myonuclei. Consistently, GO term analysis of genes upregulated in Mbnl KD myonuclei showed enrichment for pathways associated with muscle activation and remodelling (Supplementary Fig. 3a–d; Supplementary Data 4 and 5). Further CellChat analysis revealed an overall increase in ligand–receptor interactions toward MuSCs in the Mbnl KD condition, originating from type IIx and IIb myofibres and FAPs, and involving growth factor signalling (*Fgf*, *Egf*, *Bmp/Tgf-β*), Notch pathway activity, and extracellular matrix-related interactions (collagen/laminin, CD44/integrins) supporting MuSC activation upon Mbnl depletion (Supplementary Fig. 3e). This analysis also revealed the emergence of a distinct population of myonuclei in Mbnl KD TA muscles that was absent from control muscles (Fig. 2a). To better understand this new population, we subclustered all myonuclei (Type IIx and IIb clusters), resulting in two transcriptionally distinct subgroups. One cluster, enriched in *Mbnl* KD muscles, was marked by high expression of *Ankrd1*, a gene associated with muscle stress response, developmental reprogramming and response to muscle overload^44,45^. The second subgroup, uniquely found in *Mbnl* KD muscles, was defined by the expression of *Meg3* and multiple transcripts from the *Dlk1*-*Dio3* imprinted locus (Fig. 2b), a genomic region implicated in embryonic development and stem cell maintenance^46^.

Pathway enrichment analysis of the *Meg3*^+^ myonuclear population indicated a strong association with muscle contraction, actin dynamics, and mechanical stimulus-response pathways, including upregulation of genes such as *Srgap3* and *Rp1* (Fig. 2c; Supplementary Data 6). In contrast, the *Ankrd1*^+^ population was enriched for pathways involved in myogenic development and expressed canonical myogenic regulatory factors (Fig. 2d; Supplementary Data 7), including *Myod1* and *Myog*, suggesting an intermediate or activated myogenic state.

To contextualise these transcriptional states within normal muscle development, we compared our snRNA-seq data with publicly available datasets from postnatal day 10 (P10) and day 21 (P21) TA muscles^47^. This comparison revealed that *Meg3*^+^ myonuclei peaked early during development (P10) and declined at later stages, while *Ankrd1*^+^ myonuclei emerged at P21 and were absent in fully mature adult muscle (Supplementary Fig. 4a, b). These findings suggest that both *Meg3*^+^ and *Ankrd1*^+^ populations represent immature and transitional myonuclear states during normal muscle maturation.

The reappearance of both *Meg3*^+^ and *Ankrd1*^+^ myonuclear populations in adult *Mbnl* KD muscles, where they are normally absent under physiological conditions, suggests that these nuclei correspond to immature or transitional states that are reactivated in the context of MBNL depletion. Their presence may reflect the incorporation of activated MuSC nuclei into existing fibres through myonuclear accretion, rather than classical regeneration following degeneration. To assess whether these transcriptional states are specific to MBNL depletion or reflect a more general response to muscle perturbation, we compared our data with published snRNA-seq datasets from muscle 14 days after sciatic nerve transection^48^, a well-established model of myofibre stress and transcriptional remodelling. Despite expected differences in the myonuclei composition, we observed a similar population of *Ankrd1*^+^ myonuclei in the denervated animals that was absent in the controls. Finally, we re-analysed data from sorted myonuclei after overload^49^ and observed a marked increase in *Ankrd1* expression and the appearance of the *Meg3*^+^ population, similar to what was observed in Mbnl KD muscle. Collectively, this suggests that these populations represent a common myonuclear response to muscle perturbation and remodelling, rather than a process specific to MBNL depletion, and do not imply that denervation or injury contributes to the Mbnl-KD phenotype (Supplementary Fig. 4c, d).

### *Mbnl* knockdown in myofibres activates MuSCs

**Fig. 3:**
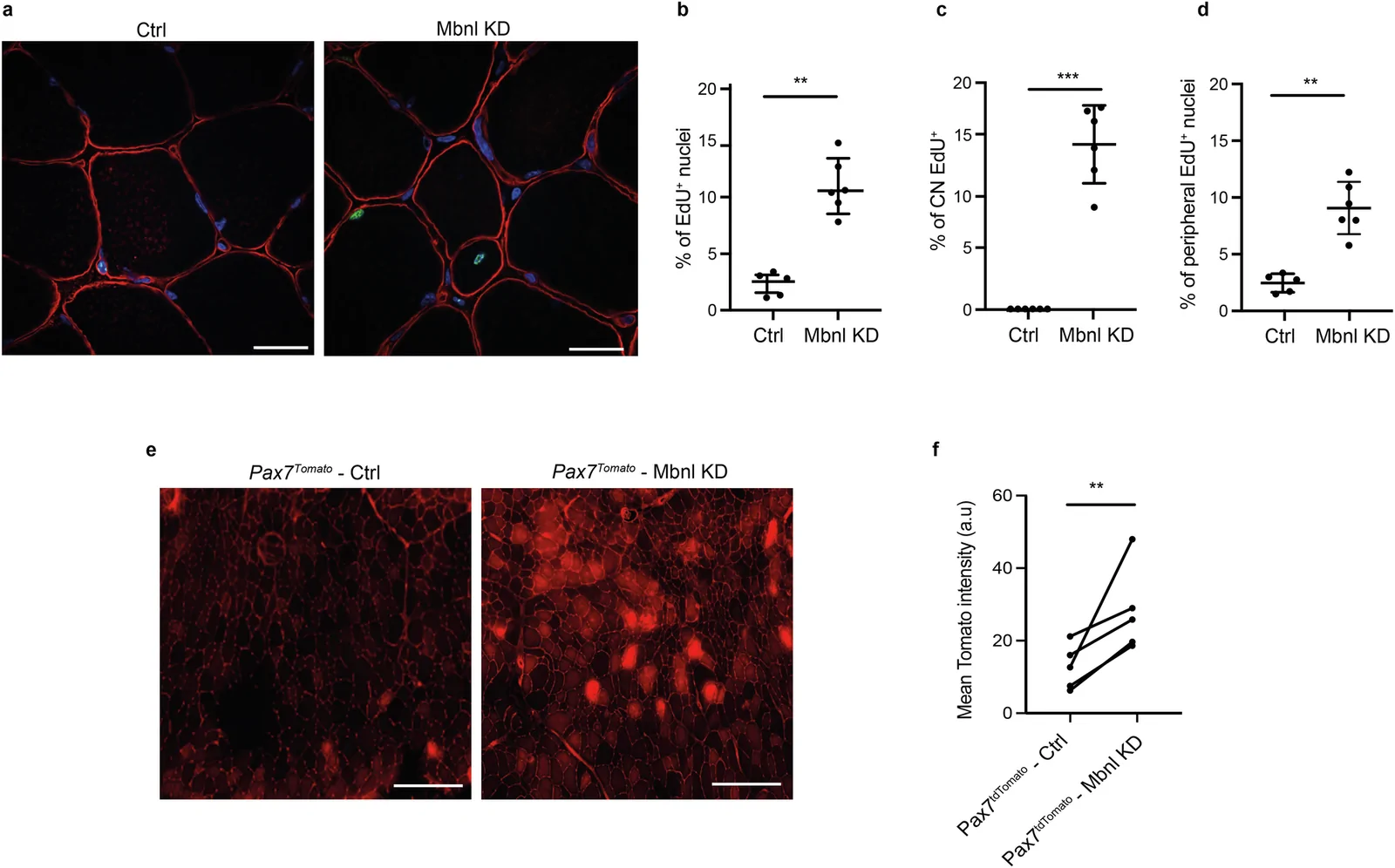
Validation of MuSC activation and fusion following Mbnl knockdown in adult skeletal myofibres. **a** Transverse TA muscle sections showing EdU signal (green), laminin (red), and Hoechst (blue). Scale bars: 20 μm. **b** Percentage of myonuclei positive for EdU in transverse sections (*n* = 5 Ctrl and *n* = 6 Mbnl KD muscles). **c** Percentage of centronucleated myofibres containing at least one EdU-positive central nucleus (*n* = 6 muscles per group). **d** Percentage of peripheral myonuclei positive for EdU in transverse sections (*n* = 5 Ctrl and *n* = 6 Mbnl KD muscles). **e** Transverse muscle sections showing the localisation of the tdTomato signal. Scale bar: 50 μm. **f** Quantification of mean tdTomato signal intensity in muscle sections (*n* = 5 muscles per group). Ctrl and Mbnl KD muscles were contralateral muscles from the same animals, and individual mice represent biological replicates. For quantification on transverse muscle sections, at least 1500 fibres were analysed. Data represent mean ± SD; *p < 0.05, **p < 0.01, ***p < 0.001; two-sided paired *t*-tests. Exact *p* values are provided in the Source data file.

Bulk and snRNA-seq analyses suggested that *Mbnl* KD promotes MuSCs activation in adult mouse TA muscles. To directly evaluate this observation, we performed EdU (5-ethynyl-2′-deoxyuridine) incorporation assays over a 5-week period following intramuscular injection of AAV-*Mbnl*-shmiR starting 5 weeks after intramuscular injection of AAV-*Mbnl*-shmiR (Fig. 3a). At the endpoint, Mbnl KD TA muscles exhibited 11% EdU-positive nuclei, including 10% peripheral and 15% centrally located EdU-positive myonuclei, compared to only 2.5% EdU incorporation in control TA muscles (Fig. 3b–d). These results confirm that MBNL depletion stimulated cell division in a subset of nuclei that have fused with existing post-mitotic fibres, a process consistent with activation and cell cycle re-entry of MuSCs before their fusion.

To further validate the involvement of MuSCs, we performed Mbnl KD in *Pax7*^*CreERT2*^; *R26R*^*tdTomato*^ reporter mice, where MuSCs and their progeny are labelled with tdTomato following tamoxifen administration. In this model, *Mbnl* KD resulted in an increased number of tdTomato-positive myofibres as well as a robust increase in total tdTomato fluorescence intensity (Fig. 3e, f), indicating that activated MuSCs had fused with existing myofibres.

**Fig. 4:**
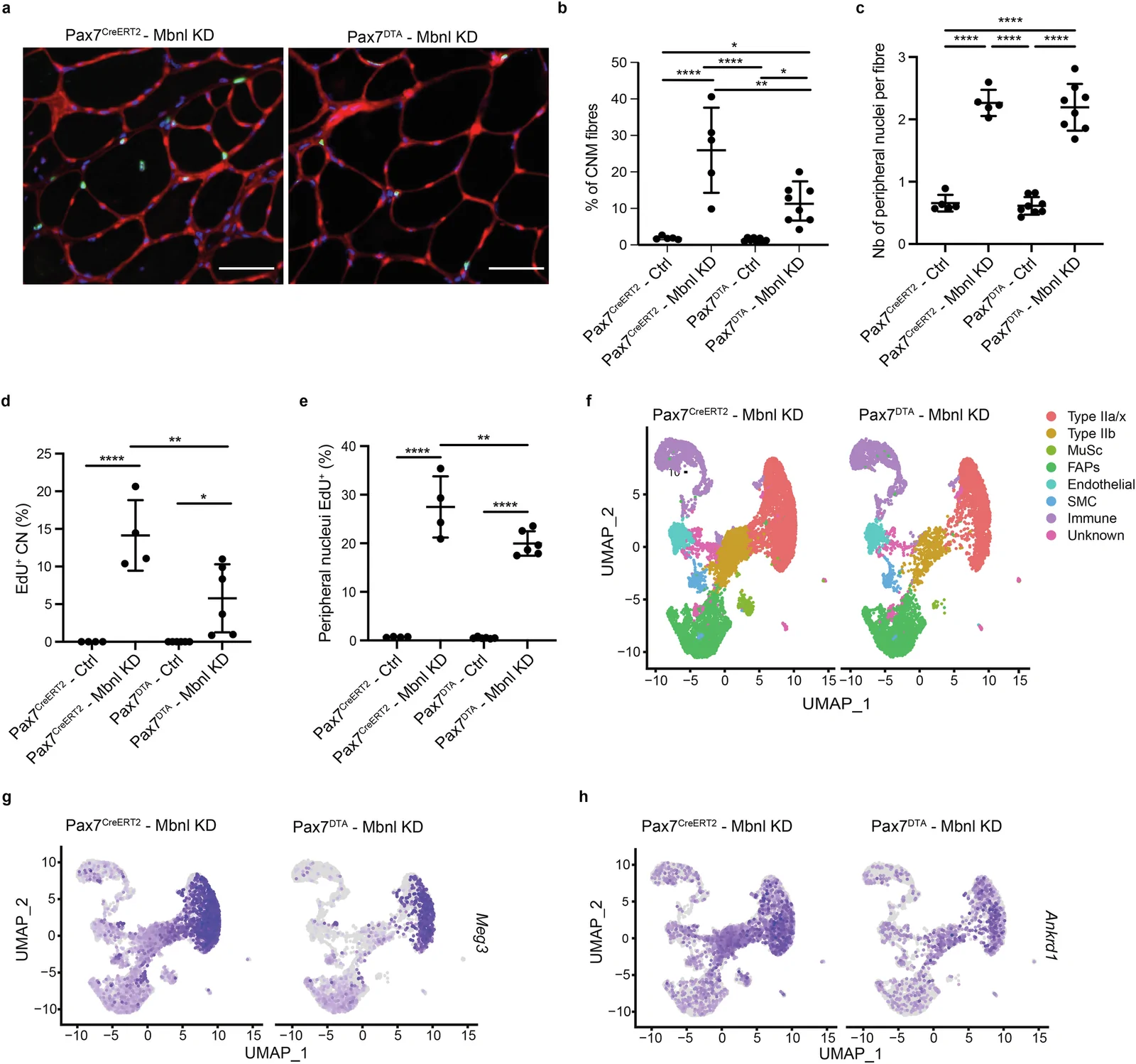
Pax7⁺ MuSC contribution to myonuclear accretion following Mbnl knockdown in adult skeletal muscle fibres. **a** Transverse TA muscle sections from Pax7^CreERT2^ and Pax7^DTA^ mice showing EdU signal (green), laminin (red) and Hoechst (blue). Scale bars: 50 μm. **b** Percentage of muscle fibres with central nuclei in transverse muscle sections. **c** Number of peripheral nuclei per muscle fibre in transverse sections. **d** Percentage of centronucleated muscle fibres containing at least one EdU-positive central nucleus. **e** Percentage of EdU-positive peripheral nuclei. **f** snRNA-seq UMAP plot of nuclear transcripts derived from Mbnl knockdown in Pax7^CreERT2^ and Pax7^DTA^ TA muscles. The colours identify different nuclear populations. **g** UMAP plot for *Meg3* transcript and **h** *Ankrd1* transcript derived from Mbnl knockdown in Pax7^CreERT2^ and Pax7^DTA^ TA muscles. Statistical analysis: For panels b and c, *n* = 5 Pax7^CreERT2^ Ctrl, *n* = 5 Pax7^CreERT2^ Mbnl KD, *n* = 8 Pax7^DTA^ Ctrl and *n* = 8 Pax7^DTA^ Mbnl KD muscles. For (**d**, **e**), *n* = 4 Pax7^CreERT2^ Ctrl, *n* = 4 Pax7^CreERT2^ Mbnl KD, *n* = 6 Pax7^DTA^ Ctrl and *n* = 6 Pax7^DTA^ Mbnl KD muscles. For quantification on transverse muscle sections, at least 1500 fibres were analysed. Data represent mean ± SD; *p < 0.05, **p < 0.01, ***p < 0.001, ****p < 0.0001; one-way ANOVA followed by Tukey’s multiple-comparisons test. Exact *p* values are provided in the Source data file.

Next, we used *Pax*^*7CreERT2*^ *R26R*^*DTA*^ mice, in which Cre-mediated activation of the diphtheria toxin A (DTA) chain in Pax7⁺ cells results in the cell-autonomous ablation of muscle stem cells following tamoxifen treatment (Fig. 4; Supplementary Fig. 5a–c). In this context, Mbnl KD resulted in a significant reduction in the proportion of centrally nucleated fibres compared to Mbnl KD in *Pax7*^*CreERT2*^ control mice, while the number of total and peripheral nuclei remained the same. However, no significant changes in functional readouts, including myotonia and specific force, were observed following MuSC deletion and reduced central nucleation (Fig. 4a–c; Supplementary Fig. 5d–g). At the same time, control AAV injections had no effect. These results indicate that the emergence of centralised nuclei in *Mbnl*-deficient muscles is MuSC-dependent. However, the preservation of peripheral myonuclear number in Pax7^DTA^ Mbnl KD TA compared to Pax7^*CreERT2*^ Mbnl KD TA raises the possibility that peripheral nuclei may derive from non-Pax7 muscle progenitor populations or other unidentified sources. To directly assess the contribution of Pax7⁺ MuSCs to newly incorporated myonuclei, we performed EdU incorporation experiments in Pax7^DTA^ mice. EdU labelling revealed a significant decrease in both EdU⁺ centralised nuclei, EdU⁺ peripheral myonuclei and subsequent total nuclei in Pax7^DTA^ Mbnl KD muscles compared to Mbnl KD muscles from Pax7^*CreERT2*^ control mice (Fig. 4d, e; Supplementary Fig. 5h). These findings indicate that newly incorporated nuclei, particularly centralised nuclei, are largely derived from Pax7⁺ MuSCs, and that a subset of newly added peripheral myonuclei also originates from Pax7⁺ MuSCs. The persistence of total peripheral myonuclear number despite reduced EdU incorporation may likely reflect the long-term stability of pre-existing myonuclei. To determine whether *Meg3*^+^ and *Ankrd1*^+^ nuclear populations in Mbnl KD TA depend on MuSC-derived nuclei, we performed snRNA-seq on muscles from Pax7^DTA^ mice 10 weeks after Mbnl KD (Pax7^DTA^ Mbnl KD) and compared them to Mbnl KD muscles from Pax7^*CreERT2*^ control mice (Pax7^*CreERT2*^ Mbnl KD) (Fig.4f; Supplementary Fig. 6a). Using this approach, we first confirmed efficient depletion of Pax7^+^ cells, with an ~85% reduction following DTA expression (Supplementary Fig. 6b). Notably, Pax7 depletion resulted in a partial reduction of the *Meg3*⁺/*Ankrd*1⁺ nuclear subpopulation including myogenesis markers (e.g. *Myod1*, *MyoG*) (Fig. 4g, h; Supplementary Fig. 6c, d), indicating that while Pax7⁺ MuSCs contribute to the emergence of these nuclei, they are not their sole source. Together, these data support a model in which Pax7⁺ MuSCs are the principal source of centralised nuclei and contribute partially to peripheral myonuclear accretion following Mbnl knockdown, while additional MuSC-independent mechanisms likely underlie the full emergence of *Meg3*^+^ and *Ankrd1*^+^ myonuclear populations.

Together, these in vivo mouse experiments demonstrate that dual knockdown of *Mbnl1* and *Mbnl2* in adult post-mitotic myofibres promotes MuSCs activation, proliferation, and fusion into myofibres, leading to increased myonuclear content and centronucleation.

### Human DM1 muscle biopsies show PAX7^+^ activation

We next asked whether similar cellular dynamics occur in human DM1 muscle. Specifically, we sought to determine (i) whether MuSCs are increased and/or activated in DM1 patient muscle in situ, and (ii) whether human DM1 muscle contains transcriptionally distinct myonuclear populations consistent with fusion-associated transcriptional transition states identified in the mouse model. For this purpose, muscle biopsy specimens from three healthy controls and three DM1 patients were selected and histologically characterised. To reflect the variability of the disease, muscle specimens from patients carrying different repeat expansion sizes were selected: 100, 481 (±64), and 1171 (±334) CTG-repeats, respectively (blood repeat length; Table 1). All three patients were affected by myotonia (hand grip and percussion): patient DM1_1 was walking supported by a cane, patient DM1_2 was wheelchair-bound, and patient DM1_3 was still ambulatory.

**Table 1:** Information about controls and DM1 patients donating muscle biopsy specimens

ID	Age at biopsy (decade)	Sex	Age of onset (decade)	Muscle biopsied	Blood cell Repeat length
Ctrl_1	3rd	M		Musculus biceps brachii	n.d.
Ctrl_2	4th	F		Musculus biceps brachii	n.d.
Ctrl_3	4th	F		Musculus biceps brachii	n.d.
DM1_1	7th	M	5th	Musculus biceps brachii	100
DM1_2	4th	M	2nd	Musculus quadratus femoris	481 ± 64
DM1_3	4th	F	1st	Musculus biceps brachii	1171 ± 334

**Fig. 5:**
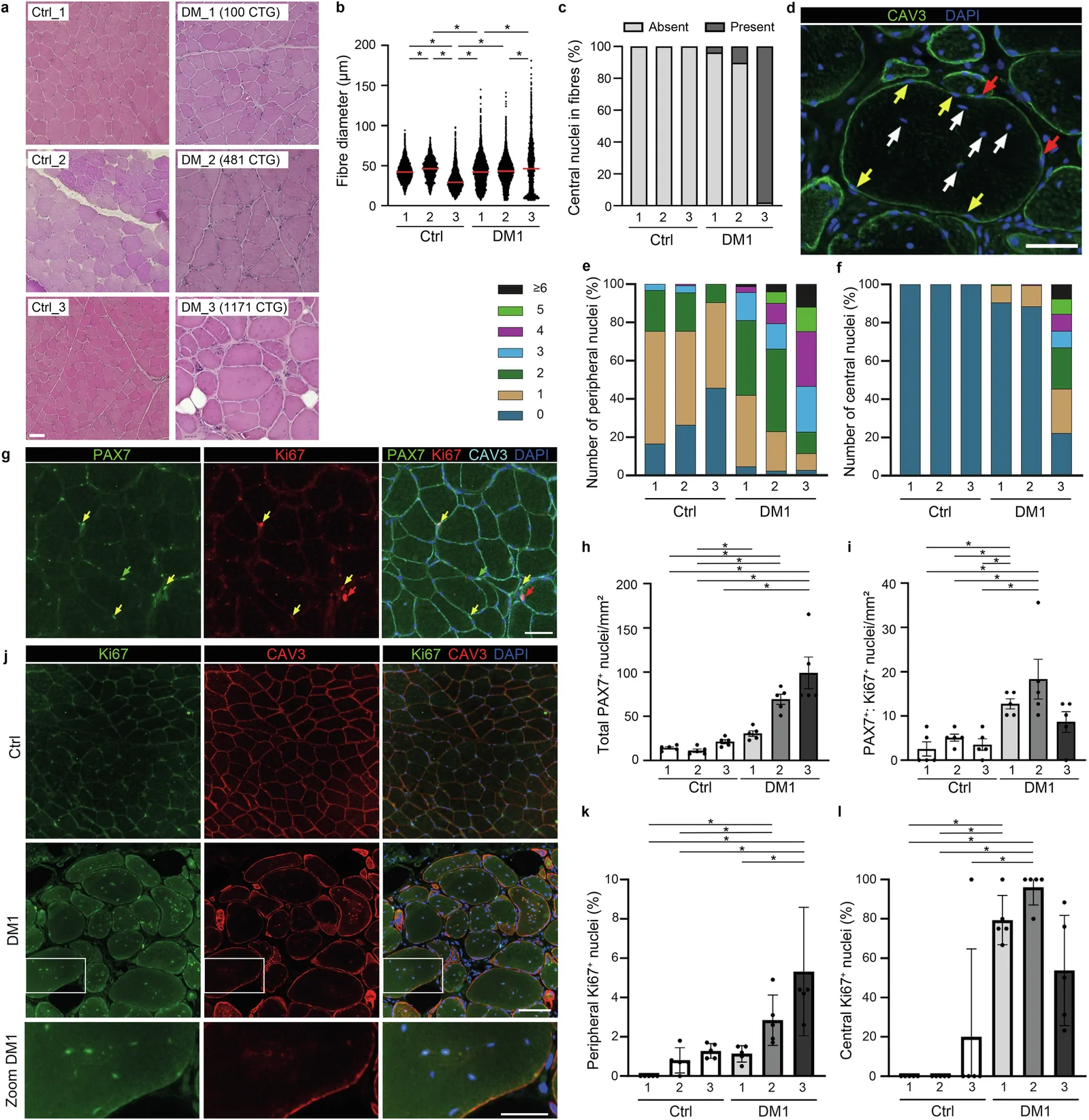
Human muscle biopsies of DM1 patients display characteristic histopathological changes and activated MuSC. **a** Hematoxylin and eosin (H&E) staining of skeletal muscle biopsies of all patients. CTG repeat expansions (measured in blood cells) are indicated for each DM1 patient in brackets. Scale bars: 50 µm. **b** Fibre diameter quantification in patients and controls. **c** Percentage of fibres with central nuclei assessed by H&E staining. **d** Representative image illustrating nuclear positioning: peripheral (yellow arrow), central (white arrow), and external (red arrow). Scale bar: 50 µm. **e** Quantification of peripheral nuclei per fibre; pattern codes for nuclei counts are indicated on the right. **f** Central nuclei quantification for DM1 and control muscle specimens. **g** Representative immunofluorescence image of DM1 muscle tissue for PAX7 and Ki67. Scale bar: 50 µm. **h** Quantification of PAX7⁺ nuclei. **i** Quantification of PAX7^+^/Ki67^+^ nuclei. **j** Representative immunofluorescence image of DM1 (DM1_3) and control (Ctrl_2) muscle tissue showing Ki67-positive myonuclei. Scale bar: 100 µm. **k**, **l** Proportion of Ki67-positive myonuclei by nuclear position. For (**b**, **h**, **i**, **k**, **l**): *n* = 3 control donors and *n* = 3 DM1 patients; individual donors or patients represent biological replicates. Five fields were quantified per individuum to calculate donor or patient values. Data are mean values ± SD. Statistical significance was assessed using Kruskal–Wallis tests with two-sided Dunn’s rank-based pairwise comparisons. False discovery rate was controlled using the two-stage step-up procedure of Benjamini, Krieger and Yekutieli. FDR-adjusted *p* values, reported as *q* values, were used to determine significance. Where indicated, *q < 0.05. Exact *q* values are provided in the Source data file.

The fibre diameter showed an increased range in all three DM1 muscle specimens (Fig. 5a, b), with controls ranging from 8 to 98 µm and DM1 muscle specimens ranging from 7 to 181 µm (Fig. 5b). While there were almost no myofibres containing central nuclei present in the healthy control muscle specimens, all three DM1 muscle specimens had an increased number of fibres containing central nuclei, which increased with repeat length. In patient DM1_3, the highest number of central nuclei was detected, with almost all fibres presenting with central nuclei (Fig. 5c, d; raw counts corresponding to Fig. 5c are provided in Supplementary Fig. 7).

Quantification of peripheral nuclei per myofibre (based on 10 µm cross-sections; Fig. 5d) revealed that most control fibres contained between zero and two subsarcolemmal nuclei. In contrast, DM1 muscle specimens exhibited an increased number of peripheral nuclei, which correlated with CTG repeat length. In DM1_1, up to five nuclei per fibre were observed, with approximately 5% of fibres containing four or five nuclei. This proportion increased in DM1_2 and DM1_3, where up to six or more peripheral nuclei per fibre were detected. Notably, approximately 25% of fibres in DM1_2 and 55% in DM1_3 harboured four or more peripheral nuclei (Fig. 5e). Muscle biopsies from DM1_1 and DM1_2 exhibited approximately 10% of fibres containing a single central nucleus. In contrast, fibres from DM1_3 displayed a greater number of central nuclei, with up to six nuclei per fibre observed (Fig. 5f; nuclear counts normalised to tissue area are shown in Supplementary Fig. 8).

All three DM1 muscle samples exhibited increased numbers of PAX7-positive nuclei relative to controls (Fig. 5g, h). The number of PAX7-positive nuclei (per mm^2^) increased with repeat length (Fig. 5h). Co-staining of Ki67 and PAX7 showed an increased number of double-positive nuclei across all DM1 muscle specimens (Fig. 5i). Strikingly, there were also Ki67-positive nuclei detected within myofibres (Fig. 5j), and the majority of these were centralised (Fig. 5k, l).

### Single-nucleus RNA-sequencing of human DM1 muscle biopsies reveals transcriptionally distinct myonuclear populations

**Fig. 6:**
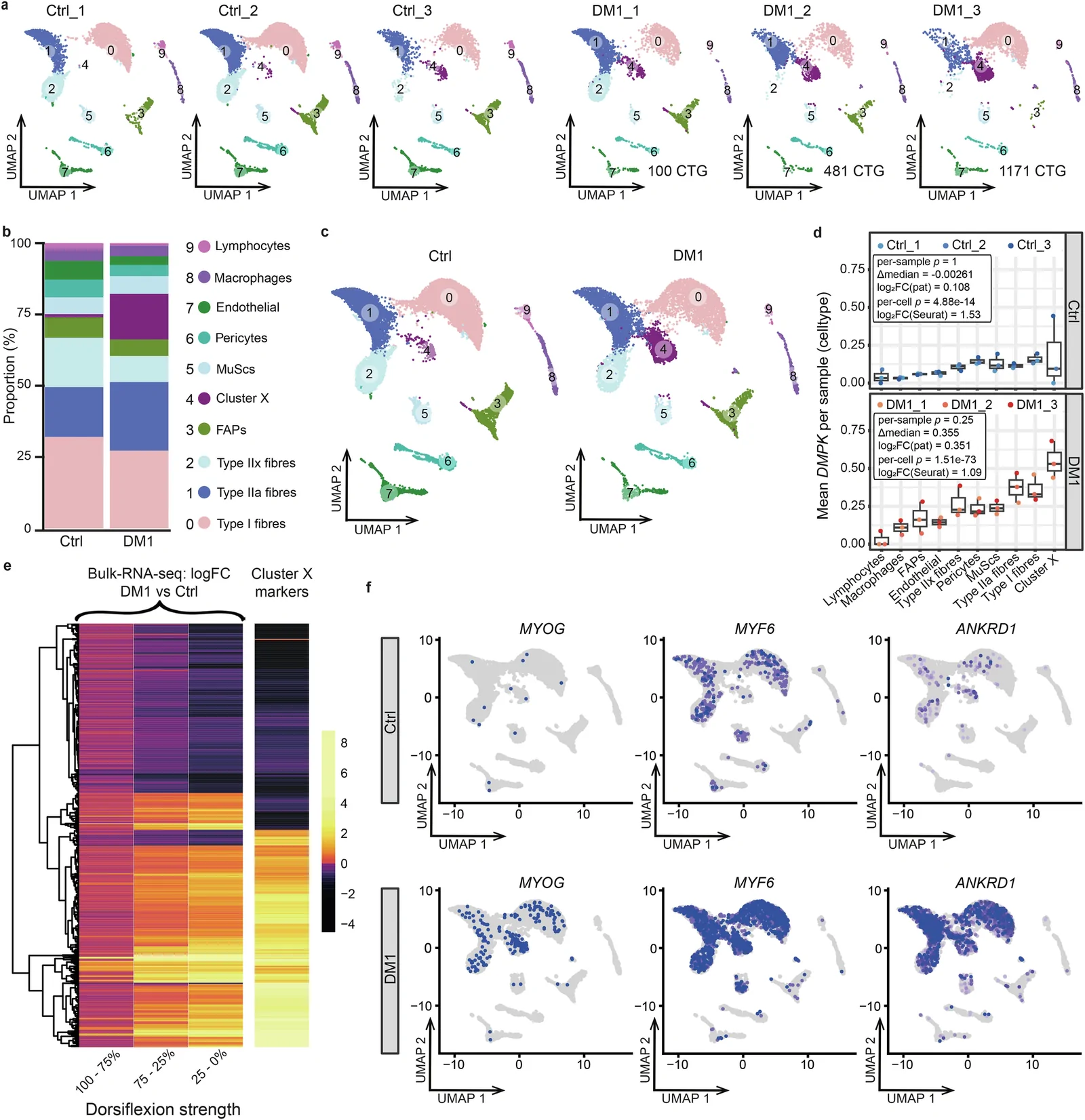
snRNA-seq of human muscle biopsies reveals an immature myonuclear expression profile in DM1 compared to controls. **a** UMAP clustering of all analysed nuclei from control and DM1 muscle specimens (shown per individual) revealed 10 distinct clusters. **b** Cell type distribution across control and DM1 specimens. **c** UMAP clustering of all analysed nuclei from control and DM1 muscle specimens (pooled). **d** *DMPK* expression in all cell-type clusters in DM1 and controls, representing the combined signal from both the wild-type and mutant alleles. **e** Expression of 933 Cluster X marker genes analysed in a bulk RNA-seq dataset from unrelated DM1 patients (11 controls, 43 patients). Cluster X marker genes were identified using Seurat’s FindConservedMarkers function by comparing Cluster X nuclei with all other myonuclei. Differential expression was assessed using a Wilcoxon rank-sum test with Benjamini–Hochberg correction for multiple testing. Genes with adjusted *p* < 0.05 and absolute log₂FC > 0.5 in DM1 samples were retained for downstream analysis. The heatmap displays log₂FC values relative to controls (left three panels, bulk RNA-seq) and to other myonuclei (right panel, snRNA-seq). Quantile-based colour scaling was used to enhance contrast for genes with moderate fold changes. **f** Expression of *MYOG* and *MYF6*, and *ANKRD1* per nucleus. For (**d**), *n* = 3 control donors and *n* = 3 DM1 patients were analysed; individual donors or patients represent biological replicates. Individual points represent patient- or donor-level mean *DMPK* expression within the indicated cell-type cluster. Box plots show the median centre line, the 25th and 75th percentiles as box limits, and whiskers extending to the most extreme data points. Statistical significance was assessed using a two-sided paired Wilcoxon signed-rank test.

Single-nucleus RNA-sequencing of DM1 and control human muscle specimens revealed shifts in the proportion of nuclei across several clusters, which were assigned to specific cell types based on marker gene expression (Supplementary Fig. 9). In DM1 muscle specimens, a reduced proportion of nuclei compared to controls was observed in clusters 0 (type I fibres), 2 (type IIx fibres), 3 (fibro/adipogenic progenitors, FAPs), 6 (pericytes), 7 (endothelial cells), and 9 (lymphocytes), with type IIx nuclei showing the most marked depletion (Fig. 6a, b; Supplementary Fig. 10). Type IIx fibre-associated nuclei showed marked inter-individual variability across DM1 muscle biopsies. Although some samples showed patterns consistent with immunostaining-based fibre typing (Supplementary Fig. 11), no statistically supported conclusion regarding IIx fibre abundance can be drawn from this dataset. The proportions of nuclei in clusters 5 (muscle stem cells, MuSCs) and 8 (macrophages) remained unchanged. By contrast, clusters 1 (type IIa fibres) and 4 were expanded in DM1, with cluster 4 displaying the largest relative increase (Fig. 6a, b; combined UMAP plots shown in Fig. 6c).

Cluster 4 expressed markers of mature myonuclei such as *MYH2*, but also showed high expression of the MuSC marker *NCAM1* (Supplementary Fig. 9). Although *NCAM1* is typically associated with immature or denervated fibres, canonical regeneration markers (*MYH3*, *MYH8*) were detected in <10% of nuclei—higher than in other clusters but still limited. We therefore refer to this transcriptionally mixed population as Cluster X.

As *DMPK* encodes the CTG repeat expansion responsible for DM1, we next mapped its expression across clusters. *DMPK* was consistently enriched in slow-twitch myonuclei across all three DM1 patients. Fast-twitch nuclei (types IIa and IIx) showed increased *DMPK* only in patients 2 and 3, coinciding with fibre-type-specific abnormalities, including fibre size variability. Outside of slow fibres, *DMPK* was notably enriched only in Cluster X, but not in canonical MuSCs (Fig. 6d). Importantly, the abundance of Cluster X nuclei correlated with CTG repeat length (Fig. 6a). Across samples, MBNL1 expression showed a tendency to be reduced in most myonuclear populations in DM1 muscle (normalised value ~3.0) compared with controls (normalised value ~4.0, *p* value 0.1); however, this trend was not observed in Cluster X. Notably, Cluster X was rare or absent in two of the three control samples but was more prominent in one control sample (CTRL_3), which also exhibited higher MBNL1 expression within this cluster (~4.0 in CTRL_3 and ~3.0 in DM1s). Given the limited cohort size and inter-individual variability, we did not observe statistically significant differences and therefore interpret these findings as descriptive rather than disease-specific (Supplementary Fig. 12).

Given the expansion of Cluster X in DM1 muscle and its specific *DMPK* accumulation, we evaluated the broader disease relevance of its gene signature. To this end, we reanalysed a publicly available bulk RNA-seq dataset from the tibialis anterior muscles of 43 DM1 patients and 11 controls^50^. Patients were stratified into three functional groups based on dorsiflexion strength (100–75%, 75–25%, and 25–0%) as defined in the original study. We identified 933 genes (Supplementary Data 8) that were significantly differentially expressed in Cluster X compared to other myonuclear clusters (Fig. 6e). While most of these genes were unchanged in patients with preserved dorsiflexion strength, a subset—including *TENM2*, *MYH8*, *CHRND*, and *FHOD1*—was consistently altered across all clinical groups. Approximately 90% of Cluster X marker genes showed concordant regulation in patients with moderate weakness (75–25% strength), and this pattern was further accentuated in the most severely affected individuals.

We next asked whether DM1 myonuclei—specifically Cluster X—exhibit features of transcriptional immaturity. Expression of the long non-coding RNA *MEG3* and the myogenic factor *ANKRD1*, previously identified as markers of a distinct myonuclear transcriptional state in our mouse model, revealed cell-type-specific patterns: *MEG3* remained restricted to MuSCs and FAPs in both control and DM1 muscle specimens, whereas *ANKRD1*—low in control fibres—was broadly upregulated across all myonuclear fibre types in all 3 DM1 patients. Furthermore, the expression of canonical myogenic regulatory factors revealed elevated levels of late differentiation markers *MYOG* and *MYF6* in DM1 myonuclei (Fig. 6f; Supplementary Fig. 13). In bulk RNA-seq data from 43 patients^50^, both *ANKRD1* and *MYF6* were significantly upregulated and correlated with clinical measures of muscle weakness (Supplementary Fig. 14).

### Cluster X shares features with single-nucleated cell types and regenerating myonuclei

**Fig. 7:**
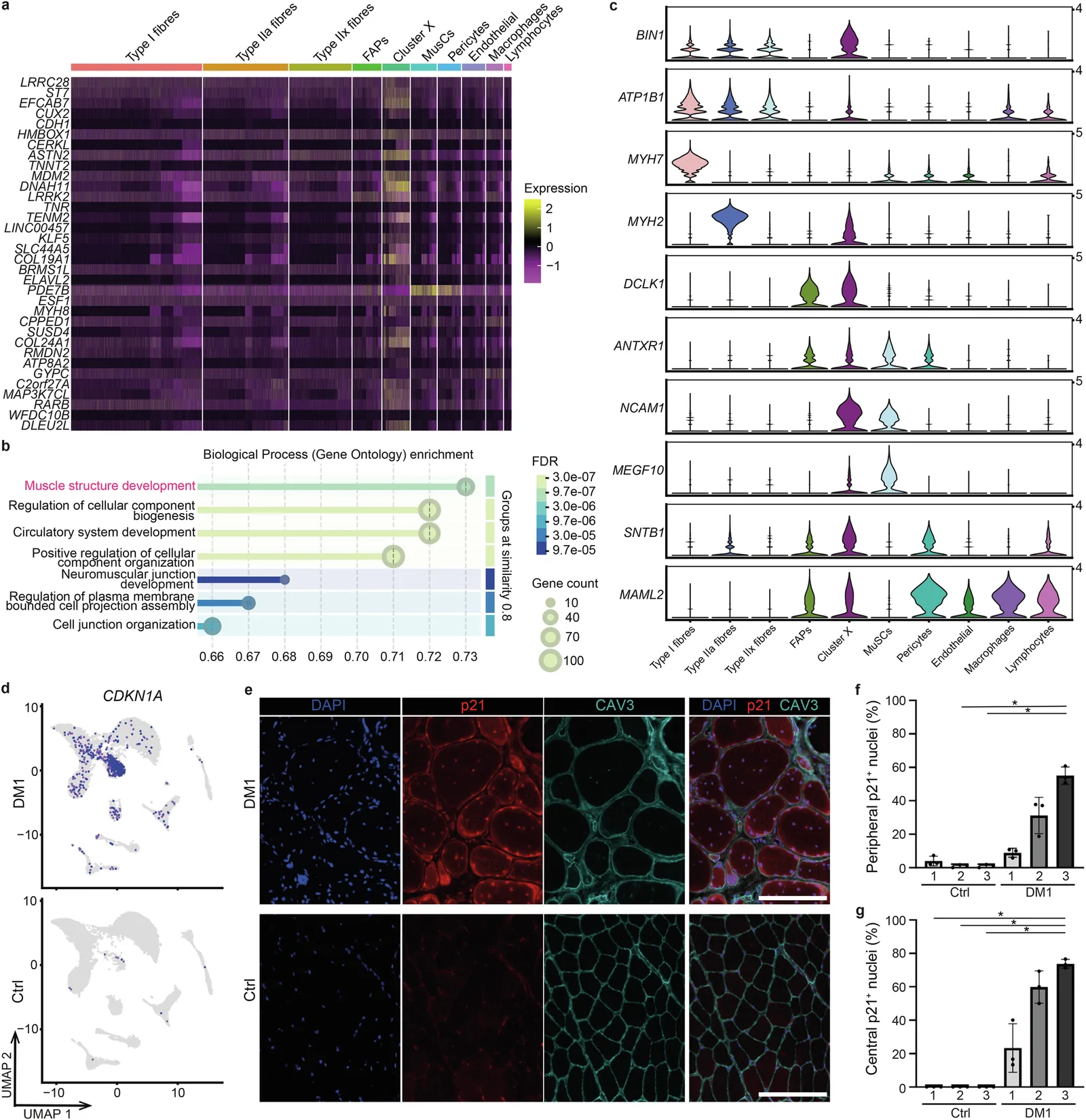
Cluster X shares gene expression features with muscle progenitor cells and expresses the cell cycle exit gene *CDKN1A.* **a** Heatmap showing the top 35 Cluster X marker genes (log₂FC > 0.5 or <−0.5, adjusted *p* < 0.05 vs. all other myonuclei) across annotated clusters. Differential expression was performed on pseudobulk RNA-seq counts using DESeq2; *p* values were calculated with the DESeq2 Wald test and adjusted using the Benjamini–Hochberg procedure. **b** Gene Ontology (GO) enrichment analysis of Cluster X marker genes (log₂FC > 0.5, *p* < 0.05). Dot size indicates gene ratio and colour indicates adjusted *p* value. **c** Violin plots illustrating the hybrid expression profile of Cluster X. **d** Feature plot showing expression of the cell cycle inhibitor *CDKN1A* (p21), a prominent Cluster X marker. **e** Representative immunofluorescence staining for p21 in muscle tissue from patient DM1_3 and control Ctrl_2 confirming protein-level expression in myonuclei. Scale bars: 200 µm. **f**, **g** Quantification of p21-positive peripheral and central myonuclei. For (**f**, **g**): *n* = 3 control donors and *n* = 3 DM1 patients were analysed; individual donors or patients represent biological replicates. Three fields were quantified per donor or patient and used to calculate the corresponding donor or patient value. Data are presented as mean values ± SD. Differences among independent groups were assessed using Kruskal–Wallis tests followed by two-sided Dunn’s rank-based pairwise comparisons. False discovery rate was controlled separately for each outcome at *Q* = 5% using the two-stage step-up procedure of Benjamini, Krieger and Yekutieli. FDR-adjusted *p* values, reported as *q* values, were used to determine significance. Where indicated, *q < 0.05. Exact *q* values are provided in the Source data file.

Among the genes significantly enriched in Cluster X relative to both fast- and slow-twitch myonuclei (Fig. 6e), the top marker transcripts included several associated with embryonic myogenesis and muscle regeneration, such as *MYH8*, *COL19A1*, and *COL24A1* (Fig. 7a). Notably, the transcription factor KLF5, which acts in cooperation with MyoD to drive myoblast differentiation^51^, was also enriched in Cluster X. Consistent with the identity of these marker genes, GO term enrichment analysis identified ‘muscle structure development’ as the most significantly enriched term among Cluster X marker genes (Fig. 7b). These results indicate that Cluster X is characterised by a transcriptional programme enriched for genes associated with developmental and remodelling-related annotations and shows strong similarity to the transcriptional patterns of the *Ankrd1*⁺ myonuclear cluster identified in the mouse model.

We next investigated the possible cellular origins of Cluster X nuclei. To test this, we examined the expression of canonical myonuclear genes alongside markers typically restricted to other mononucleated, muscle-resident cell types. While Cluster X expressed typical muscle-specific genes such as *ATP1B1* and *MYH2*, their levels were lower than in mature myonuclei. Conversely, *BIN1* appeared upregulated, and *MYH7* was notably absent (Fig. 7c). We also detected expression of *DCLK1* (doublecortin-like kinase 1), a gene previously implicated in myoblast activity and regeneration. Another Cluster X enriched gene, *ANTXR1* (also known as TEM8, encoding a mechanosensitive ECM-interacting adhesion molecule) was also expressed in MuSCs, pericytes, and endothelial cells, but not in canonical myonuclei. Similarly, *NCAM1* and *MEGF10* were enriched in MuSCs, while *SNTB1* and *MAML2* were broadly expressed across mononucleated muscle-resident populations yet largely excluded from mature myonuclei (Fig. 7c).

Among the Cluster X markers, which were also significantly upregulated in bulk RNA-seq data from 43 DM1 patients, we identified *CDKN1A*, encoding p21—a cyclin-dependent kinase inhibitor typically induced upon cell cycle exit. Although *CDKN1A* expression is highly enriched in Cluster X, it is not exclusive to this population, as moderate expression was also observed in other myonuclei (Fig. 7d). Given that p21 localises to the nucleus when active, we selected *CDKN1A* as a candidate marker to facilitate the identification of Cluster X in situ. Immunostaining confirmed an increased number of p21-positive nuclei in all three DM1 specimens, which were found almost exclusively within myofibres. Notably, mostly centrally located nuclei were positive for p21 (Fig. 7e–g).

Finally, to assess the extent to which these myonuclear states are conserved between Mbnl KD in mouse TA muscle and DM1 patient biopsies, we compared the snRNA-seq datasets generated from Mbnl KD mouse muscles with human DM1 muscle snRNA-seq data. Cross-species integration revealed that *Ankrd1*⁺ myonuclei in mice broadly overlapped with the overall population of DM1 myonuclei in human samples, encompassing but not restricted to Cluster X (Supplementary Fig. 15). In addition, comparison of marker genes distinguishing *Meg3*⁺ myonuclei, *Ankrd1*⁺ myonuclei, and human Cluster X nuclei revealed comparable degrees of overlap between these populations. Specifically, 32 of 236 marker genes (~13%) overlapped between *Meg3*⁺ and Cluster X nuclei, while 205 of 1666 marker genes (~12%) overlapped between *Ankrd1*⁺ and Cluster X nuclei (Supplementary Data 9). Collectively, the genes shared between mouse and human myonuclear populations are associated with biological processes central to muscle pathology, myogenic differentiation and developmental signalling (*NFIB, LTBP1, PDZRN3*), muscle structural integrity (*KY, FRAS1*), stress-activated and adaptive signalling pathways (*MAP3K14, KCNN3, SLC24A2*), supporting the involvement of conserved programmes of muscle remodelling and stress response in DM1 muscle upon MBNL loss of function.

## Discussion

One of the striking and unexplained features of muscle pathology in DM1 is the unusually high frequency of centrally located nuclei in small and large fibres^5,34^. Central nuclei are typically considered hallmarks of canonical muscle regeneration following necrotic degeneration. However, in DM1, they appear in the absence of overt necrosis or inflammation and are detectable even at early stages of the disease, including in minimally symptomatic individuals^5^. While incomplete regeneration has been proposed as one mechanism^52^, defects in myonuclear migration linked to altered nucleo-cytoskeletal integrity^53,54^ as well as persistent mechanical stress—particularly in the context of myotonia—could also be potential contributors.

Here, we provide evidence that MuSCs directly fuse with existing fibres and contribute to the accumulation of central nuclei in DM1 skeletal muscle. Fusion of MuSC-derived cells with damaged but surviving fibres has been proposed as a repair mechanism^55^; however, this process has not been directly demonstrated in vivo. Instead, repair of sublethal myofibre damage has been shown to occur through MuSC-independent mechanisms involving redistribution of resident myonuclei, while MuSC fusion to existing fibres is most clearly supported in the context of exercise-induced hypertrophy and myonuclear accretion^56^. Our findings, therefore, provide direct in vivo evidence in a mammalian model of DM1, supported by observations in human DM1 muscle, that MuSC fusion contributes to myonuclear accretion under pathological conditions in DM1 muscle. Whether this fusion reflects an adaptive repair response, is induced by chronic mechanical stress (for example, due to myotonia), or represents a combination of both, and whether it is ultimately beneficial, remains unclear. Centrally located nuclei were confirmed in skeletal muscle biopsies from DM1 patients, included and carrying different CTG repeat lengths, along with a higher number of myonuclei per fibre compared to unaffected individuals. Concomitantly, an increased number of PAX7^+^ and Ki67^+^ nuclei, including Ki67^+^ nuclei located within myofibres, was detected in DM1 muscle specimens, suggesting that fusion of activated MuSCs contributes to myonuclear accretion. These findings are consistent with prior in situ observations of increased MuSC abundance in DM1 muscle biopsies^34^ as well as increased numbers of central nuclei and myonuclei per fibre in the HSA-LR transgenic mouse model^57^.

To dissect the specific contribution of MBNL loss-of-function, we developed an inducible, myofibre-specific knockdown model targeting Mbnl1 and Mbnl2 in adult mice, thereby avoiding developmental confounders. We demonstrate that MBNL depletion in post-mitotic myofibre is sufficient to recapitulate molecular and histological changes characteristic of DM1, such as alternative splicing and myotonia, but also an increased number of myonuclei per fibre and centralised nuclei. Importantly, Mbnl-KD in myofibres triggered MuSC activation, proliferation and myofibre fusion in the absence of major muscle degeneration or necrosis. MBNL proteins are key regulators of RNA metabolism, and their depletion in skeletal muscle alters splicing and expression of key fibre-derived factors, such as extracellular matrix proteins, cytokines, or cell adhesion molecules, which are known to modulate MuSC fate^15,27,28^. Alternatively, metabolic or mechanical changes in MBNL-deficient fibres may also contribute to MuSC activation indirectly through signalling.

However, muscles displaying myofibre-specific MBNL knockdown did not exhibit atrophy, a typical feature of late-stage DM1. Instead, muscle mass was increased, potentially reflecting a compensatory mechanism in which activated MuSCs, still expressing MBNL, contribute to myonuclear accretion and maintenance of muscle homeostasis. Critically, genetic ablation of Pax7^+^ MuSCs in vivo prevented the increase of centralised nuclei in Mbnl-KD muscles, confirming activated MuSC fusion as their origin. However, this intervention did not result in measurable improvements in muscle function, including myotonia or force generation, indicating that MuSC-mediated fusion is not a primary driver of the functional phenotype.

To further refine the interpretation of these findings, particularly with respect to EdU-based lineage tracing, it is important to consider potential alternative sources of EdU incorporation. While EdU incorporation is commonly interpreted as evidence of newly fused MuSC-derived nuclei, it is important to note that EdU labels DNA synthesis rather than cell division. In the absence of MuSCs, EdU-positive myonuclei could reflect alternative processes such as DNA repair, endoreduplication, or contribution from non-Pax7-derived progenitor populations. Given that mature myonuclei are considered post-mitotic, direct division of pre-existing myonuclei is unlikely, but cannot be excluded.

Single-nucleus RNA-seq analysis of Mbnl-KD muscles further supported this mechanism. We identified distinct myonuclear subpopulations that expressed transcriptional signatures reminiscent of early developmental stages and cellular stress, despite residing in mature muscle^47,58,59^. One subpopulation retained expression of genes contained in the *Dlk1*-*Dio3* locus (including *Meg3*), which is typically silenced during myogenic maturation^60^. *Meg3*, a long non-coding RNA, has been implicated in the maintenance of myogenic cell identity during myoblast progression into differentiation and regeneration^46,61^. A second subpopulation was characterised by a high expression of *Ankrd1*, a mechanosensitive gene associated with regenerating or stressed myonuclei, suggesting a role in sarcomere remodelling and repair during regeneration and muscle physiological changes^48,49,58,59^. Together, these data indicate reactivation of developmental programmes in myofibres of Mbnl-KD adult mice, implicating MuSC-derived nuclei as the source. Taken together, these findings suggest that MuSC-mediated fusion represents a prominent histopathological feature contributing to myonuclear remodelling and heterogeneity, but has a limited direct impact on overt muscle dysfunction in this model, which is likely primarily driven by intrinsic defects in MBNL-deficient myofibres. Although MuSC activation is clearly observed in response to MBNL depletion, the underlying trigger remains unresolved. Our findings support a model in which MBNL-deficient myofibres alter the muscle microenvironment, thereby promoting MuSC activation and fusion in the absence of overt degeneration. Potential contributing factors may include altered mechanical signalling, metabolic stress, or changes in paracrine signalling; however, the precise molecular drivers remain to be identified.

To gain a more comprehensive understanding of DM1 muscle pathology, we performed snRNA-seq on human DM1 muscle specimens, providing the first transcriptional profiling of individual muscle nuclei in this context. This analysis identified a transcriptionally distinct myonuclear population, termed Cluster X, characterised by a hybrid transcriptional identity. Cluster X nuclei co-expressed markers typically associated with mononucleated muscle progenitors (*NCAM1*, *DCLK1*, *ANTXR1*) alongside mature myonuclear genes such as *MYH2*. These nuclei were also enriched for *DMPK* transcripts and expressed *CDKN1A*, encoding the cell cycle inhibitor p21. Immunostaining for p21 confirmed widespread nuclear localisation in central nuclei, consistent with a state of recent or ongoing cell cycle exit. Together with elevated numbers of Ki67^+^ central nuclei, these findings suggest that Cluster X represents a transitional state in which proliferating cells have fused with myofibres but have not yet completed terminal differentiation. Ki67 and p21/CDKN1A stainings were performed on separate sections and were not co-localised in the current study; therefore, we cannot determine marker overlap at single-nucleus resolution. We interpret Ki67⁺ nuclei as reflecting proliferative activity (primarily pre-fusion), whereas p21⁺ central myonuclei likely represent a post-fusion, cell-cycle-exit/arrest state associated with the immature/transitional myonuclear programme. Although our data do not establish a causal mechanism, the persistence of transcriptionally immature myonuclei in DM1 muscle is consistent with a model in which RNA-mediated toxicity and MBNL loss of function interfere with the normal transcriptional maturation of newly incorporated nuclei.

Hybrid or transitional states of myonuclear subpopulations have already been described during postnatal myonuclear maturation and regeneration^47^. However, under normal physiological conditions, these states are transient and do not persist in mature, healthy muscle. In DM1, by contrast, the accumulation of such nuclei may reflect a stalled or delayed differentiation programme, triggered by the expression of toxic *DMPK* transcripts bearing expanded CUG repeats and the chronically stressed myofibre environment. Supporting this, as we previously reported, an independent bulk RNA-seq dataset revealed downregulation of muscle-specific nuclear envelope transmembrane proteins, normally upregulated during differentiation and involved in genome organisation, suggesting impaired nuclear maturation may be a broader feature of DM1 muscle pathology^62^. Because bulk RNA-seq reflects the combined transcriptional output of all cell types present in the biopsy, the increased abundance of Cluster X markers in the independent cohort may partly reflect differences in tissue composition (e.g. fibre-type proportions or non-myogenic content) rather than changes within a specific myonuclear population. Thus, while the bulk data provide supportive external validation, they should be interpreted as indicative rather than definitive evidence for Cluster X expansion.

Although this analysis was limited to a small number of DM1 muscle biopsies, Cluster X marker gene expression tended to increase with clinical disease severity in the independent cohort of 43 DM1 patients. While this trend is consistent with a potential link between immature myonuclear states and disease progression, formal statistical validation will require larger sample sizes and uniform clinical data. This association suggests that incompletely differentiated or immature myonuclei may contribute to DM1 muscle pathogenesis, although their functional contribution to pathology remains to be defined.

Many Cluster X-enriched genes are typically absent from canonical myonuclear populations and are instead associated with muscle progenitor cells, supporting the notion that these nuclei reflect either an immature post-fusion state or an incomplete maturation trajectory. However, it cannot be excluded that Cluster X represents a de-differentiated state re-entering a progenitor-like programme, or that it includes nuclei derived from other cell types, such as fibro-adipogenic progenitors or pericytes. Indeed, pericytes, which are recognised for their capacity to fuse with myofibres under stress^63^, may also contribute to Cluster X, particularly given their co-expression of *ANTXR1* and *MAML2*. Further investigation is required to elucidate the specific roles and fates of different muscle progenitor populations in DM1 pathology.

Beyond central nucleation, we also observed elevated numbers of peripheral myonuclei in DM1 and MBNL-depleted mouse myofibres, along with other hallmark features of DM1, including myotonia and a shift toward oxidative fibre types. It remains to be determined whether mechanical stress and/or metabolic changes of the fibres are responsible for this increase in the number of peripheral myonuclei, especially given that oxidative fibres are known to harbour more nuclei than their glycolytic counterparts^52,64–66^. Moreover, our snRNA-seq analysis also revealed that *DMPK* mRNA expression is higher in nuclei associated with oxidative fibre clusters. Importantly, markers expressed during myonuclear maturation or under stress, such as *MYF6* and *ANKRD1*, were elevated across all fibre type clusters, including Cluster X. This broad dysregulation suggests that altered transcriptional dynamics are not restricted to a specific myonuclear subtype, but rather affect most or all myonuclei in DM1 muscle.

Our snRNA-seq analysis also revealed differences between human and mouse datasets. These discrepancies may partially reflect the distinct muscle origins analysed in each species, with mouse data derived from TA muscles and human samples obtained from biceps brachii biopsies. Variations in fibre-type composition and muscle-specific transcriptional programmes could contribute to these species-specific patterns and should be considered when interpreting cross-species comparisons.

Although our findings support the idea of impaired or delayed acquisition of a mature myonuclear programme following fusion, we cannot completely rule out an alternative possibility that the observed signature reflects a sustained influx of recently fused MuSC-derived nuclei that have not yet fully completed maturation.

In conclusion, our study demonstrates that MBNL loss in post-mitotic DM1 myofibres triggers a regenerative response through MuSC activation and fusion, and leads to impaired subsequent myonuclear maturation. This study reframes central nuclei not as static remnants of prior injury, but as dynamic markers of an incomplete regenerative process. Future studies should determine whether these immature myonuclei functionally integrate into the contractile apparatus and whether promoting their maturation or limiting maladaptive fusion can restore muscle integrity.

## Methods

All research complied with all relevant ethical regulations. Animal experiments were performed in accordance with protocols APAFIS#25244-2020042716304109v6 and APAFIS#37309-202205101648455v5, approved by the Ethical Committee on Animal Resources at the Centre d’Expérimentation Fonctionnelle of the Pitié-Salpêtrière animal facility. Human muscle biopsy studies were performed with written informed consent from all participants under protocols approved by the Ethics Review Board of the National Centre of Neurology and Psychiatry, approval number B2024-130.

### Animal procedures

All mouse procedures were carried out at the Centre d’Experimentation Fonctionnelle of Pitié-Salpétrière animal facility and under appropriate biological containment. Briefly, mice were housed in a specific-pathogen-free facility with a standard 12 h:12 h light–dark cycle at 22 °C and 55% humidity with access to standard diet and water *ad libitum*. Animal experiments were performed in adult Mus musculus mice of both sexes. The mouse strains and genotypes used for each experiment are described below and in the corresponding figure legends. Unless otherwise stated, mice were 2 months of age at the time of tamoxifen treatment or AAV injection. The number of animals used in each experiment is provided in the corresponding figure legends and Source Data files. Animal experiments were reported in accordance with the ARRIVE guidelines. Sex was not used as an experimental variable because the study was designed to assess the effect of Mbnl knockdown and muscle stem cell ablation rather than sex-specific differences; both sexes were included where possible. Mice were monitored regularly for general health, behaviour, body condition and signs of distress throughout the experimental period. Animals were euthanised before tissue collection in accordance with the approved animal study protocols.

### Mouse strains

Mouse lines used in this study have been described and provided by the corresponding laboratories: FVB mice (Janvier Labs^®^), *Pax7*^*creERT2/+*^ mice^67^ *Rosa26*^*flox(stop)flox-Tomato*^ (*R26*^*stop-Tomato*^)^68^, *Rosa26*^*flox(stop)/flox-DTA*^*(Gt(ROSA)26Sor*^*tm1(DTA)Jpmb*^*)*^69^. Animal crossing allowed the generation of *Pax7*^*creERT2/+*^; *Rosa26*^*flox(stop)flox-Tomato*^ and *Pax7*^*creERT2/+*^; *Rosa26*^*flox(stop)/flox-DTA*^.

### Construct

Short hairpin microRNA (shmiR) expression constructs were generated using the BLOCK-iT™ Pol II miR RNAi Expression Vector Kit (Invitrogen), following the manufacturer’s instructions. The shRNA sequences targeting murine Mbnl1 and Mbnl2 were based on previously published designs^14^ (Mbnl1 shRNA sequence: GAAGTATGTAGAGAGTTTCAA; Mbnl2 shRNA sequence: CGGCTATTAGCTTTGCTCCTT), while a control shRNA sequence targeting lacZ was used as a negative control (LacZ shRNA sequence: AAATCGCTGATTTGTGTAGTC).

### Tamoxifen treatment

To induce recombination, 2-month-old adult *Pax7*^*creERT2/+*^; *Rosa26*^*flox(stop)flox-Tomato*^ and *Pax7*^*creERT2/+*^; Rosa26^*flox(stop)/flox-DTA*^ mice received intraperitoneal injections of tamoxifen (Sigma-Aldrich, CAS # 10540-29-1) at a dose of 75 mg/kg body weight once daily for 5 consecutive days.

### In vivo gene transfer

AAV2/9 vectors were generated using a three-plasmid production system. Two-month-old adult FVB WT, *Pax7*^*creERT2/+*^; *Rosa26*^*flox(stop)flox-Tomato*^ and *Pax7*^*creERT2/+*^; *Rosa26*^*flox(stop)/flox-DTA*^ mice were injected in the left TA with 40 μl of AAV2/9-shmiR-Mbnl1-Mbnl2 (5 × 10^10^ viral genomes). The contralateral TA received AAV2/9-shmiR-lacZ (5 × 10^10^ viral genomes). Mice were euthanised 10 weeks post-injection; muscles were collected, snap-frozen in liquid nitrogen-cooled isopentane, and stored at −80 °C. AAV injections in Pax7 reporter lines were performed 7 days post-tamoxifen induction.

### EdU incorporation

Five weeks after AAV injection, mice received intraperitoneal injections of 5-ethynyl-2′-deoxyuridine (EdU) (40 mg/kg body weight) twice weekly for 5 weeks. Tissues were collected and processed using the Click-iT™ EdU Imaging Kit (Invitrogen), per the manufacturer’s protocol.

### In situ isometric force analysis

Ten weeks after AAV injection, in situ isometric contraction of the TA muscle was assessed as previously described^70^. Mice were anaesthetised with pentobarbital sodium (60 mg/kg, intraperitoneally) and kept on a temperature-controlled warm plate to ensure stable physiological conditions during the experiments. The TA tendon was attached to a force transducer (305B, Aurora Scientific); sciatic nerves were stimulated using supramaximal square-wave pulses (0.1 ms). Maximal tetanus-induced force (100 Hz, 500 ms stimulation) was recorded. Force data were collected via PowerLab and LabChart v8 (ADInstruments). Myotonia was derived from the area under the relaxation curve (AUC), normalised by tetanic force to account for variations in maximal contractile strength. Blinded analyses were performed throughout the assessment. Mice were euthanised via cervical dislocation following the procedure, and muscles were harvested for further analysis.

### Muscle histopathology

Transverse TA muscle sections (5 µm) were stained with Hoechst (Invitrogen) and rabbit anti-Laminin (Abcam) or Wheat Germ Agglutinin (Thermo Fisher Scientific) for plasma membrane visualisation. Images were captured using an Olympus BX60 microscope and Metamorph software (Molecular Devices). Confocal images were captured using a Nikon Ti2 microscope equipped with a motorised stage and a Yokogawa CSU-W1 spinning disk head coupled with a Prime 95 sCMOS camera (Photometrics). Images were processed using Zen software (Zeiss), and quantitative analysis was performed with ImageJ (Fiji) and the MuscleJ plugin^71^. Analyses were performed blinded to genotype or treatment group to ensure unbiased measurements. Fibre cross-sectional areas were quantified on all fibres from a single transverse section of the TA muscle. For quantification of myonuclei per fibre, nuclei were counted in single transverse sections; thus, values represent the number of nuclei intersected in the section rather than the total nuclear content of each myofibre.

### Bulk RNA-sequencing

TA muscles were homogenised in TriReagent (Sigma-Aldrich) using the FastPrep system with Lysing Matrix D tubes (MP Biomedicals). RNA was extracted per the manufacturer’s protocol and quality-checked with the TapeStation 2200 (Agilent). Libraries were prepared using the KAPA mRNA HyperPrep kit (Roche) from 250 ng input RNA, and sequenced on an Illumina NovaSeq 6000 with 150 bp paired-end reads (140M reads/sample). Reads were processed with FastQC, Cutadapt (v2.10), and aligned using STAR (v2.7.5a) to the GRCm38/mm10 mouse genome. Transcript quantification used HTSeq (v0.12.4). All steps were integrated into a Nextflow (v.20.04.1) pipeline. Differential gene expression analysis was performed with DESeq2 [false discovery rate (FDR) < 0.05; log_2_ fold-change > 1]; alternative splicing was analysed via rMATS (v4.1.2; FDR corrected *P* < 0.05, delta per cent spliced in (ΔPSI) ≥ 20%). Analysis of GO terms and biological pathways enrichment was performed using Enrichr^72,73^ (GO Biological process^74,75^).

### Single-nucleus RNA-sequencing: mouse tissue

Ten to fifteen cuts of 50 µm muscle tissue were cut using a cryostat and maintained frozen before isolation. Nuclei were isolated as previously described^76^, with some modifications optimised for the muscle. Notably, 2% BSA was added to the same-day homogenisation buffer. RNAase inhibitor (RNasin Plus Ribonuclease Inhibitor – Promega) was added to all solutions before use, at 200 Units per mL. Samples were homogenised using a Dounce homogeniser (12× pestle A, 16× pestle B). Samples were filtered through 100, 70, and 30 µm strainers. Nuclei were counted and loaded for library prep using the Chromium Next GEM Single Cell 3′ Kit v3.1 (10x Genomics) (20,000 nuclei were loaded per reaction to target 10,000 nuclei). Libraries were sequenced on a NextSeq2000.

### Bioinformatics: mouse snRNA-seq

Fastq files were aligned to mm10 using CellRanger v6 (10x Genomics). Downstream analysis in Seurat v5^77^ included ambient RNA correction (SoupX)^78^, doublet removal (DoubletFinder)^79^, and quality filtering (>5% mitochondrial reads excluded). Dimensionality reduction, clustering, and differential expression (using the DeSeq2 method) were implemented using Seurat v5. Clustering and UMAP analyses were performed using default parameters, as implemented in the analysis pipeline. Integration between the different datasets was done using the SCT transform method. To compare our data to other studies, the R objects were downloaded from the respective papers^47,59^. For the Petrany et al. data, we used Seurat v5 to integrate the data from P10, P21, and 5 months with our data. For the Kim et al. data, as it was not generated using a 10x Genomics platform, we integrated the data within the different manipulations. Cell-to-cell signalling predictions were done with CellChat^80^. Briefly, wildtype and knockdown snRNA-seq datasets were analysed with the CellChat programme using the default parameters and then merged to compare the signalling intensities between the two genotypes.

### Reverse transcription qPCR

Two hundred nanograms of RNA was reverse transcribed using SuperScriptIV™ first-strand synthesis system (Life Technologies) in a total of 20 μL. To quantify the mRNA expression, real-time PCR was performed using a Lightcycler 480 (Roche). Reactions were performed with SYBR Green kit (Roche) according to the manufacturer’s instructions. PCR cycles were a 15-min denaturation step followed by 50 cycles with a 94 °C denaturation for 15 s, 58 °C annealing for 20 s and 72 °C extension for 20 s. Mouse *Rplp0* mRNA was used as standard. Data were analysed with the Lightcycler 480 analysis software. Primers sequences: mouse Pax7-FW: AGGACGACGAGGAAGGAGACA; mouse Pax7-rev: TCATCCAGACGGTTCCCTTT; mouse Mbnl1-FW: CGGGACACAAAATGGCTAAC; mouse Mbnl1 rev: TTGCAGTTCTCTCTGGAGCA; mouse Mbnl2-FW: TTTTCCCACATCCTCCAAAG and mouse Mbnl2-rev: GAATGTGTCAGCAAGCAGGA.

### Human subjects and sample collection

Muscle biopsies were obtained from three genetically confirmed DM1 patients and three histopathologically normal control subjects. DM1 diagnoses were established via Southern blot analysis for CTG repeat expansions in the *DMPK* gene. Control samples were confirmed to be negative for neuromuscular pathology and known genetic mutations at the time of analysis.

### DNA library preparation for Nanopore CRISPR/Cas9-targeted resequencing

To analyse CTG repeat length in *DMPK* (Patients 2 and 3), DNA libraries were constructed using the Ligation Sequencing Kit (Oxford Nanopore Technologies, SQK-LSK109). Cas9 ribonucleoprotein (RNP) complexes were assembled by incubating annealed tracrRNA-crRNA (1 μM) with HiFi Cas9 protein (0.5 μM; IDT, #1081061) for 30 min at room temperature. Guide RNA pairs targeting regions flanking the *DMPK* repeat were designed using the CRISPR Custom Design Tool from Integrated DNA Technologies (IDT) (https://sg.idtdna.com/site/order/designtool/index/CRISPR_CUSTOM) and synthesised by IDT (Supplementary Data 10).

Genomic DNA (2 μg) was dephosphorylated with Quick Calf Intestinal Phosphatase (NEB, #M0525S) at 37 °C for 10 min and heat-inactivated at 80 °C for 2 min. The treated DNA was then incubated with Cas9 RNPs, Taq polymerase (NEB, #M0273S), and dATP (NEB, #N0440S) at 37 °C for 30 min to allow for targeted cleavage, followed by dA-tailing at 72 °C for 5 min.

For barcoding, the cleaved and dA-tailed DNA was ligated to barcodes using the Native Barcoding Expansion 1–12 kit (Oxford Nanopore Technologies, EXP-NBD104) and Blunt/TA Ligase Master Mix (NEB, #M0367L) at room temperature for 10 min. The ligated products were purified using AMPure XP magnetic beads (Beckman Coulter, #A63880). Subsequently, AMII adaptors were added using Quick T4 DNA Ligase (NEB, #E6056S) under the same conditions, and the reaction was again purified using AMPure XP beads.

The final DNA library was loaded onto a MinION Flow Cell (FLO-MIN106D) and sequenced using the MinION Mk1C platform for 20–21 h. Basecalling was performed using Guppy to generate Fastq files. Reads were aligned to the reference sequence (GRCh38) using Minimap2 and then visualised using Integrative Genomics Viewer^81,82^.

### Histopathological analysis of human specimens

Cryosections (10 μm thick) were prepared from frozen muscle tissue of DM1 patients and controls. Standard haematoxylin and eosin (H&E) staining and immunohistochemistry were performed. Primary antibodies are listed in Supplementary Data 11. Alexa Fluor-conjugated secondary antibodies (Invitrogen; 1:600) were used for detection. Imaging was performed on a BZ-X700 microscope (KEYENCE). Fibre size was quantified in full transverse sections (1900–8700 fibres). The nuclear positioning was quantified from five random area (260-958 fibres per patient muscles). MuSC quantification was performed by counting PAX7-positive nuclei across five randomly selected fields per sample.

### Nuclei isolation from human frozen muscle tissue

The isolation of nuclei from human subjects was described previously^83^. Briefly, tissues stored at −80 °C were sectioned and placed into ice-cold lysis buffer (Tris-HCl pH 7.4, NaCl, MgCl₂, and NP-40). Following the addition of bovine serum albumin wash buffer with RNase Inhibitor (TOYOBO, #SIN-201), tissue homogenisation was performed using a Dounce homogeniser attached to a Wheaton Overhead Stirrer with 10 strokes while on ice. The homogenate was sequentially filtered through 100 µm and 40 µm cell strainers. Nuclei were stained with 7-AAD and incubated on ice for 15 min before fluorescence-activated nuclei sorting using a BD FACSAria Fusion flow cytometer.

### Single-nucleus RNA-sequencing (snRNA-seq) library preparation—human

Single-nucleus RNA-seq libraries were generated using the Chromium Next GEM Single Cell 3′ Reagent Kit v3.1 (Dual Index) from 10x Genomics (Pleasanton, CA, USA), in accordance with the manufacturer’s protocol. The procedure included cDNA synthesis, amplification, and library construction. Library quality was verified using the Agilent 2100 Bioanalyzer with a High Sensitivity DNA Kit (Agilent Technologies). Paired-end, dual-index sequencing was performed using NovaSeq 6000 (Illumina) with a target depth of 50,000–100,000 reads per nucleus.

### Bioinformatic analysis of human snRNA-seq data

Sequencing reads were aligned to the GRCh38 human reference genome using CellRanger v6 (10x Genomics). Count matrices were analysed using Seurat v5 in R. Nuclei with >5% mitochondrial RNA content were excluded. DoubletFinder was used to eliminate predicted doublets. Normalisation and integration of datasets were carried out using the SCTransform workflow with reciprocal PCA. Clustering and differential expression analyses were performed within Seurat v5 using DESeq2.

To evaluate the disease relevance of Cluster X, we re-analysed a publicly available bulk RNA-seq dataset of tibialis anterior muscle from 43 DM1 patients and 11 controls^50^. FASTQ files were mapped to the GRCh38 human reference genome, and differential expression analysis was performed using DESeq2. Patients were stratified by dorsiflexion strength into three clinical groups (proto-DM1, classic DM1, and severe DM1), as described in the original publication. Cluster X marker genes were defined using Seurat’s FindConservedMarkers function, with thresholds of adjusted *p* < 0.05 and log₂ fold-change > 0.5 or <−0.5. These were then used to subset and compare gene expression profiles in the bulk dataset.

### Statistical analysis

All statistical analyses were conducted using GraphPad Prism (versions 8 and 9) and R (version 4.2.0). Normality of data was assessed using the Shapiro–Wilk test. Depending on distribution and variance, group comparisons were performed using paired *t*-tests for two-group comparisons, and the Wilcoxon matched-pairs signed rank test for proportions comparison. For three or more groups, one-way ANOVA or two-way ANOVA were applied, followed by appropriate post-hoc tests. Correlations between repeat length and clinical parameters were assessed using Pearson’s correlation coefficient. A *p* value of <0.05 was considered statistically significant.

### Reporting summary

Further information on research design is available in the Nature Portfolio Reporting Summary linked to this article.

## Supplementary information

Supplementary Information

Description of Additional Supplementary Files

Supplementary Data 1–11

Reporting Summary

Transparent Peer Review file

## Source data

Source data

## Data Availability

The single-nucleus RNA-sequencing and bulk messenger RNA-sequencing data generated in this study have been deposited in the Gene Expression Omnibus (GEO) database under accession codes GSE262646 (mouse single-nucleus RNA-sequencing after MBNL knockdown), GSE335902 (mouse single-nucleus RNA-sequencing after Pax7-DTA-mediated muscle stem cell ablation), and GSE335961 (mouse bulk messenger RNA-sequencing after MBNL knockdown). Processed sequencing data generated in this study are provided in the Supplementary Information, Supplementary Data and Source data files. Source data are provided with this paper. Human clinical metadata are not publicly available at the individual level because of participant privacy and ethical restrictions; only aggregated or pseudonymised information required to interpret the study is provided in the manuscript and associated files. Source data are provided with this paper.

## References

[1] Mahadevan, M. et al. Myotonic dystrophy mutation: an unstable CTG repeat in the 3’ untranslated region of the gene. *Science* **255**, 1253–1255 (1992). 1546325 10.1126/science.1546325

[2] Brook, J. D. et al. Molecular basis of myotonic dystrophy: expansion of a trinucleotide (CTG) repeat at the 3’ end of a transcript encoding a protein kinase family member. *Cell* **68**, 799–808 (1992). 1310900 10.1016/0092-8674(92)90154-5

[3] Fu, Y. H. et al. An unstable triplet repeat in a gene related to myotonic muscular dystrophy. *Science* **255**, 1256–1258 (1992). 1546326 10.1126/science.1546326

[4] Wenninger, S., Montagnese, F. & Schoser, B. Core clinical phenotypes in myotonic dystrophies. *Front. Neurol* **9**, 303 (2018). 29770119 10.3389/fneur.2018.00303 PMC5941986

[5] Harper, P. *Myotonic Dystrophy* (OUP Oxford, 2009).

[6] De Antonio, M. et al. Unravelling the myotonic dystrophy type 1 clinical spectrum: a systematic registry-based study with implications for disease classification. *Rev. Neurol.* **172**, 572–580 (2016). 27665240 10.1016/j.neurol.2016.08.003

[7] Ho, G., Cardamone, M. & Farrar, M. Congenital and childhood myotonic dystrophy: current aspects of disease and future directions. *World J. Clin. Pediatr.* **4**, 66–80 (2015). 26566479 10.5409/wjcp.v4.i4.66 PMC4637811

[8] Meola, G. & Cardani, R. Myotonic dystrophies: an update on clinical aspects, genetic, pathology, and molecular pathomechanisms. *Biochim. Biophys. Acta* **1852**, 594–606 (2015). 24882752 10.1016/j.bbadis.2014.05.019

[9] Cumming, S. A. et al. Genetic determinants of disease severity in the myotonic dystrophy type 1 OPTIMISTIC cohort. *Neurology* **93**, e995–e1009 (2019). 31395669 10.1212/WNL.0000000000008056 PMC6745735

[10] Overend, G. et al. Allele length of the DMPK CTG repeat is a predictor of progressive myotonic dystrophy type 1 phenotypes. *Hum. Mol. Genet.* **28**, 2245–2254 (2019). 31220271 10.1093/hmg/ddz055 PMC6586140

[11] Davis, B. M., McCurrach, M. E., Taneja, K. L., Singer, R. H. & Housman, D. E. Expansion of a CUG trinucleotide repeat in the 3’ untranslated region of myotonic dystrophy protein kinase transcripts results in nuclear retention of transcripts. *Proc. Natl. Acad. Sci. USA* **94**, 7388–7393 (1997). 9207101 10.1073/pnas.94.14.7388 PMC23831

[12] Taneja, K. L., McCurrach, M., Schalling, M., Housman, D. & Singer, R. H. Foci of trinucleotide repeat transcripts in nuclei of myotonic dystrophy cells and tissues. *J. Cell Biol.* **128**, 995–1002 (1995). 7896884 10.1083/jcb.128.6.995 PMC2120416

[13] Rau, F. et al. Misregulation of miR-1 processing is associated with heart defects in myotonic dystrophy. *Nat. Struct. Mol. Biol.* **18**, 840–845 (2011). 21685920 10.1038/nsmb.2067

[14] Wang, E. T. et al. Transcriptome-wide regulation of pre-mRNA splicing and mRNA localization by muscleblind proteins. *Cell* **150**, 710–724 (2012). 22901804 10.1016/j.cell.2012.06.041 PMC3428802

[15] Batra, R. et al. Loss of MBNL leads to disruption of developmentally regulated alternative polyadenylation in RNA-mediated disease. *Mol. Cell* **56**, 311–322 (2014). 25263597 10.1016/j.molcel.2014.08.027 PMC4224598

[16] Konieczny, P., Stepniak-Konieczna, E. & Sobczak, K. MBNL proteins and their target RNAs, interaction and splicing regulation. *Nucleic Acids Res.* **42**, 10873–10887 (2014). 25183524 10.1093/nar/gku767 PMC4176163

[17] Miller, J. W. et al. Recruitment of human muscleblind proteins to (CUG)(n) expansions associated with myotonic dystrophy. *EMBO J.* **19**, 4439–4448 (2000). 10970838 10.1093/emboj/19.17.4439 PMC302046

[18] Nakamori, M. et al. Splicing biomarkers of disease severity in myotonic dystrophy. *Ann. Neurol.* **74**, 862–872 (2013). 23929620 10.1002/ana.23992 PMC4099006

[19] André, L. M., van Cruchten, R. T. P., Willemse, M. & Wansink, D. G. CTG)n repeat-mediated dysregulation of MBNL1 and MBNL2 expression during myogenesis in DM1 occurs already at the myoblast stage. *PLoS ONE* **14**, e0217317 (2019). 31116797 10.1371/journal.pone.0217317 PMC6530876

[20] Cerro-Herreros, E. et al. AntimiR treatment corrects myotonic dystrophy primary cell defects across several CTG repeat expansions with a dual mechanism of action. *Sci. Adv.* **10**, eadn6525 (2024). 39383229 10.1126/sciadv.adn6525 PMC11463307

[21] Wheeler, T. M., Lueck, J. D., Swanson, M. S., Dirksen, R. T. & Thornton, C. A. Correction of ClC-1 splicing eliminates chloride channelopathy and myotonia in mouse models of myotonic dystrophy. *J. Clin. Investig.* **117**, 3952–3957 (2007). 18008009 10.1172/JCI33355 PMC2075481

[22] Fugier, C. et al. Misregulated alternative splicing of BIN1 is associated with T tubule alterations and muscle weakness in myotonic dystrophy. *Nat. Med.* **17**, 720–725 (2011). 21623381 10.1038/nm.2374

[23] Tang, Z. Z. et al. Muscle weakness in myotonic dystrophy associated with misregulated splicing and altered gating of Ca(V)1.1 calcium channel. *Hum. Mol. Genet.* **21**, 1312–1324 (2012). 22140091 10.1093/hmg/ddr568 PMC3284119

[24] Rau, F. et al. Abnormal splicing switch of DMD’s penultimate exon compromises muscle fibre maintenance in myotonic dystrophy. *Nat. Commun.* **6**, 7205 (2015). 26018658 10.1038/ncomms8205 PMC4458869

[25] Freyermuth, F. et al. Splicing misregulation of SCN5A contributes to cardiac-conduction delay and heart arrhythmia in myotonic dystrophy. *Nat. Commun.* **7**, 11067 (2016). 27063795 10.1038/ncomms11067 PMC4831019

[26] Kanadia, R. N. et al. A muscleblind knockout model for myotonic dystrophy. *Science* **302**, 1978–1980 (2003). 14671308 10.1126/science.1088583

[27] Lee, K. Y. et al. Compound loss of muscleblind-like function in myotonic dystrophy. *EMBO Mol. Med.* **5**, 1887–1900 (2013). 24293317 10.1002/emmm.201303275 PMC3914532

[28] Thomas, J. D. et al. Disrupted prenatal RNA processing and myogenesis in congenital myotonic dystrophy. *Genes Dev.* **31**, 1122–1133 (2017). 28698297 10.1101/gad.300590.117 PMC5538435

[29] Yanay, N., Rabie, M. & Nevo, Y. Impaired regeneration in dystrophic muscle—new target for therapy. *Front. Mol. Neurosci.* **13**, 69 (2020). 32523512 10.3389/fnmol.2020.00069 PMC7261890

[30] Bazgir, B., Fathi, R., Rezazadeh Valojerdi, M., Mozdziak, P. & Asgari, A. Satellite cells contribution to exercise mediated muscle hypertrophy and repair. *Cell J.* **18**, 473–484 (2017). 28042532 10.22074/cellj.2016.4714 PMC5086326

[31] Careccia, G., Mangiavini, L. & Cirillo, F. Regulation of satellite cells functions during skeletal muscle regeneration: a critical step in physiological and pathological conditions. *Int. J. Mol. Sci.* **25**, 10.3390/ijms25010512 (2023). PMC10778731 38203683

[32] Furling, D. et al. Defective satellite cells in congenital myotonic dystrophy. *Hum. Mol. Genet.* **10**, 2079–2087 (2001). 11590125 10.1093/hmg/10.19.2079

[33] Bigot, A. et al. Large CTG repeats trigger p16-dependent premature senescence in myotonic dystrophy type 1 muscle precursor cells. *Am. J. Pathol.* **174**, 1435–1442 (2009). 19246640 10.2353/ajpath.2009.080560 PMC2671374

[34] Thornell, L. E. et al. Satellite cell dysfunction contributes to the progressive muscle atrophy in myotonic dystrophy type 1. *Neuropathol. Appl. Neurobiol.* **35**, 603–613 (2009). 19207265 10.1111/j.1365-2990.2009.01014.x

[35] Meinke, P., Hintze, S., Limmer, S. & Schoser, B. Myotonic dystrophy—a progeroid disease? *Front. Neurol.* **9**, 601 (2018). 30140252 10.3389/fneur.2018.00601 PMC6095001

[36] Furling, D., Lemieux, D., Taneja, K. & Puymirat, J. Decreased levels of myotonic dystrophy protein kinase (DMPK) and delayed differentiation in human myotonic dystrophy myoblasts. *Neuromuscul. Disord.* **11**, 728–735 (2001). 11595515 10.1016/s0960-8966(01)00226-7

[37] Todorow, V. et al. Transcriptome analysis in a primary human muscle cell differentiation model for myotonic dystrophy type 1. *Int. J. Mol. Sci.* **22**, 10.3390/ijms22168607 (2021). PMC8395314 34445314

[38] Conte, T. C. et al. Clearance of defective muscle stem cells by senolytics restores myogenesis in myotonic dystrophy type 1. *Nat. Commun.* **14**, 4033 (2023). 37468473 10.1038/s41467-023-39663-3 PMC10356779

[39] Li, X., Eastman, E. M., Schwartz, R. J. & Draghia-Akli, R. Synthetic muscle promoters: activities exceeding naturally occurring regulatory sequences. *Nat. Biotechnol.* **17**, 241–245 (1999). 10096290 10.1038/6981

[40] Wang, B. et al. Construction and analysis of compact muscle-specific promoters for AAV vectors. *Gene Ther.* **15**, 1489–1499 (2008). 18563184 10.1038/gt.2008.104

[41] Rincon, M. Y. et al. Genome-wide computational analysis reveals cardiomyocyte-specific transcriptional Cis-regulatory motifs that enable efficient cardiac gene therapy. *Mol. Ther.* **23**, 43–52 (2015). 25195597 10.1038/mt.2014.178 PMC4426801

[42] Skopenkova, V. V., Egorova, T. V. & Bardina, M. V. Muscle-specific promoters for gene therapy. *Acta Nat.* **13**, 47–58 (2021). 10.32607/actanaturae.11063 PMC8084301 33959386

[43] Nitschke, L., Hu, R. C., Miller, A. N., Lucas, L. & Cooper, T. A. Alternative splicing mediates the compensatory upregulation of MBNL2 upon MBNL1 loss-of-function. *Nucleic Acids Res.* **51**, 1245–1259 (2023). 36617982 10.1093/nar/gkac1219 PMC9943662

[44] Boskovic, S. et al. The stress responsive gene ankrd1a is dynamically regulated during skeletal muscle development and upregulated following cardiac injury in border zone cardiomyocytes in adult zebrafish. *Gene* **792**, 145725 (2021). 10.1016/j.gene.2021.145725 34010705

[45] Sun, Z. et al. Single-nucleus transcriptomics reveals subsets of degenerative myonuclei after rotator cuff tear-induced muscle atrophy. *Cell Prolif.* **58**, e13763 (2025). 39435630 10.1111/cpr.13763 PMC11882757

[46] Farhadova, S. et al. The long non-coding RNA Meg3 mediates imprinted gene expression during stem cell differentiation. *Nucleic Acids Res.* **52**, 6183–6200 (2024). 38613389 10.1093/nar/gkae247 PMC11194098

[47] Petrany, M. J. et al. Single-nucleus RNA-seq identifies transcriptional heterogeneity in multinucleated skeletal myofibers. *Nat. Commun.* **11**, 6374 (2020). 33311464 10.1038/s41467-020-20063-w PMC7733460

[48] Lin, H. et al. Decoding the transcriptome of denervated muscle at single-nucleus resolution. *J. Cachexia Sarcopenia Muscle* **13**, 2102–2117 (2022). 35726356 10.1002/jcsm.13023 PMC9398230

[49] Sun, C. et al. Lineage tracing of nuclei in skeletal myofibers uncovers distinct transcripts and interplay between myonuclear populations. *Nat. Commun.* **15**, 9372 (2024). 39477931 10.1038/s41467-024-53510-z PMC11526147

[50] Wang, E. T. et al. Transcriptome alterations in myotonic dystrophy skeletal muscle and heart. *Hum. Mol. Genet.* **28**, 1312–1321 (2019). 30561649 10.1093/hmg/ddy432 PMC6452195

[51] Hayashi, S., Manabe, I., Suzuki, Y., Relaix, F. & Oishi, Y. Klf5 regulates muscle differentiation by directly targeting muscle-specific genes in cooperation with MyoD in mice. *eLife* **5**, 10.7554/eLife.17462 (2016). PMC5074804 27743478

[52] Vattemi, G. et al. Expression of late myogenic differentiation markers in sarcoplasmic masses of patients with myotonic dystrophy. *Neuropathol. Appl. Neurobiol.* **31**, 45–52 (2005). 15634230 10.1111/j.1365-2990.2004.00602.x

[53] Hintze, S., Knaier, L., Limmer, S., Schoser, B. & Meinke, P. Nuclear envelope transmembrane proteins in myotonic dystrophy type 1. *Front. Physiol.* **9**, 1532 (2018). 30425655 10.3389/fphys.2018.01532 PMC6218431

[54] Viegas, D. et al. Nuclear envelope alterations in myotonic dystrophy type 1 patient-derived fibroblasts. *Int. J. Mol. Sci.* **23**, 10.3390/ijms23010522 (2022). PMC8745202 35008948

[55] Mauro, A. Satellite cell of skeletal muscle fibers. *J. Biophys. Biochem. Cytol.* **9**, 493–495 (1961). 13768451 10.1083/jcb.9.2.493 PMC2225012

[56] Masschelein, E. et al. Exercise promotes satellite cell contribution to myofibers in a load-dependent manner. *Skelet. Muscle* **10**, 21 (2020). 32646489 10.1186/s13395-020-00237-2 PMC7346400

[57] Mankodi, A. et al. Myotonic dystrophy in transgenic mice expressing an expanded CUG repeat. *Science* **289**, 1769–1773 (2000). 10976074 10.1126/science.289.5485.1769

[58] Dos Santos, M. et al. Single-nucleus RNA-seq and FISH identify coordinated transcriptional activity in mammalian myofibers. *Nat. Commun.* **11**, 5102 (2020). 33037211 10.1038/s41467-020-18789-8 PMC7547110

[59] Kim, M. et al. Single-nucleus transcriptomics reveals functional compartmentalization in syncytial skeletal muscle cells. *Nat. Commun.* **11**, 6375 (2020). 33311457 10.1038/s41467-020-20064-9 PMC7732842

[60] Wüst, S. et al. Metabolic maturation during muscle stem cell differentiation is achieved by miR-1/133a-mediated inhibition of the Dlk1-Dio3 mega gene cluster. *Cell Metab.* **27**, 1026–1039.e6 (2018). 29606596 10.1016/j.cmet.2018.02.022

[61] Dill, T. L., Carroll, A., Pinheiro, A., Gao, J. & Naya, F. J. The long noncoding RNA Meg3 regulates myoblast plasticity and muscle regeneration through epithelial-mesenchymal transition. *Development* **148**, 10.1242/dev.194027 (2021). 33298462

[62] Todorow, V., Hintze, S., Schoser, B. & Meinke, P. Nuclear envelope transmembrane proteins involved in genome organization are misregulated in myotonic dystrophy type 1 muscle. *Front. Cell Dev. Biol.* **10**, 1007331 (2022). 36699009 10.3389/fcell.2022.1007331 PMC9868253

[63] Tatsukawa, T. et al. NG2-positive pericytes regulate homeostatic maintenance of slow-type skeletal muscle with rapid myonuclear turnover. *Stem Cell Res. Ther.* **14**, 205 (2023). 37592340 10.1186/s13287-023-03433-1 PMC10433572

[64] van Wessel, T., de Haan, A., van der Laarse, W. J. & Jaspers, R. T. The muscle fiber type-fiber size paradox: hypertrophy or oxidative metabolism? *Eur. J. Appl. Physiol.* **110**, 665–694 (2010). 20602111 10.1007/s00421-010-1545-0 PMC2957584

[65] Burleigh, I. G. Observations on the number of nuclei within the fibres of some red and white muscles. *J. Cell Sci.* **23**, 269–284 (1977). 893535 10.1242/jcs.23.1.269

[66] Schmalbruch, H. & Hellhammer, U. The number of nuclei in adult rat muscles with special reference to satellite cells. *Anat. Rec.* **189**, 169–175 (1977). 911042 10.1002/ar.1091890204

[67] Murphy, M. M., Lawson, J. A., Mathew, S. J., Hutcheson, D. A. & Kardon, G. Satellite cells, connective tissue fibroblasts and their interactions are crucial for muscle regeneration. *Development* **138**, 3625–3637 (2011). 21828091 10.1242/dev.064162 PMC3152921

[68] Madisen, L. et al. A robust and high-throughput Cre reporting and characterization system for the whole mouse brain. *Nat. Neurosci.* **13**, 133–140 (2010). 20023653 10.1038/nn.2467 PMC2840225

[69] Ivanova, A. et al. In vivo genetic ablation by Cre-mediated expression of diphtheria toxin fragment A. *Genesis* **43**, 129–135 (2005). 16267821 10.1002/gene.20162 PMC2233880

[70] Delacroix, C. et al. Improvement of dystrophic muscle fragility by short-term voluntary exercise through activation of calcineurin pathway in mdx mice. *Am. J. Pathol.* **188**, 2662–2673 (2018). 30142334 10.1016/j.ajpath.2018.07.015

[71] Mayeuf-Louchart, A. et al. MuscleJ: a high-content analysis method to study skeletal muscle with a new Fiji tool. *Skelet. Muscle* **8**, 25 (2018). 30081940 10.1186/s13395-018-0171-0 PMC6091189

[72] Xie, Z. et al. Gene set knowledge discovery with Enrichr. *Curr. Protoc.* **1**, e90 (2021). 33780170 10.1002/cpz1.90 PMC8152575

[73] Chen, E. Y. et al. Enrichr: interactive and collaborative HTML5 gene list enrichment analysis tool. *BMC Bioinform.* **14**, 128 (2013). 10.1186/1471-2105-14-128 PMC3637064 23586463

[74] Ashburner, M. et al. Gene ontology: tool for the unification of biology. The Gene Ontology Consortium. *Nat. Genet.* **25**, 25–29 (2000). 10.1038/75556 PMC3037419 10802651

[75] The Gene Ontology resource: enriching a GOld mine. *Nucleic Acids Res.* **49**, D325-D334 10.1093/nar/gkaa1113 (2021). PMC7779012 33290552

[76] Grandi, F. C., Modi, H., Kampman, L. & Corces, M. R. Chromatin accessibility profiling by ATAC-seq. *Nat. Protoc.* **17**, 1518–1552 (2022). 35478247 10.1038/s41596-022-00692-9 PMC9189070

[77] Hao, Y. et al. Dictionary learning for integrative, multimodal and scalable single-cell analysis. *Nat. Biotechnol.* **42**, 293–304 (2024). 37231261 10.1038/s41587-023-01767-y PMC10928517

[78] Young, M. D. & Behjati, S. SoupX removes ambient RNA contamination from droplet-based single-cell RNA sequencing data. *Gigascience* **9**, 10.1093/gigascience/giaa151 (2020). PMC7763177 33367645

[79] McGinnis, C. S., Murrow, L. M. & Gartner, Z. J. DoubletFinder: doublet detection in single-cell RNA sequencing data using artificial nearest neighbors. *Cell Syst.* **8**, 329–337.e4 (2019). 30954475 10.1016/j.cels.2019.03.003 PMC6853612

[80] Jin, S., Plikus, M. V. & Nie, Q. CellChat for systematic analysis of cell–cell communication from single-cell transcriptomics. *Nat. Protoc.* **20**, 180–219 (2025). 39289562 10.1038/s41596-024-01045-4

[81] Okonechnikov, K., Golosova, O. & Fursov, M. & team, U. Unipro UGENE: a unified bioinformatics toolkit. *Bioinformatics* **28**, 1166–1167 (2012). 22368248 10.1093/bioinformatics/bts091

[82] Robinson, J. T. et al. Integrative genomics viewer. *Nat. Biotechnol.* **29**, 24–26 (2011). 21221095 10.1038/nbt.1754 PMC3346182

[83] Funaguma, S. et al. Retrotrans-genomics identifies aberrant THE1B endogenous retrovirus fusion transcripts in the pathogenesis of sarcoidosis. *Nat. Commun.* **16**, 1318 (2025). 39920152 10.1038/s41467-025-56567-6 PMC11805910

