# Supplementary material for: MBNL depletion drives stem cell fusion and immature myonuclear states in myotonic dystrophy type 1
Source: Nat Commun. 2026 Aug 6;17:9476. doi: 10.1038/s41467-026-76476-6 (PMC13542076; doi:10.1038/s41467-026-76476-6)
Supplement: Supplementary file 1 [file 41467_2026_76476_MOESM1_ESM.pdf]

Supplementary Figure S1

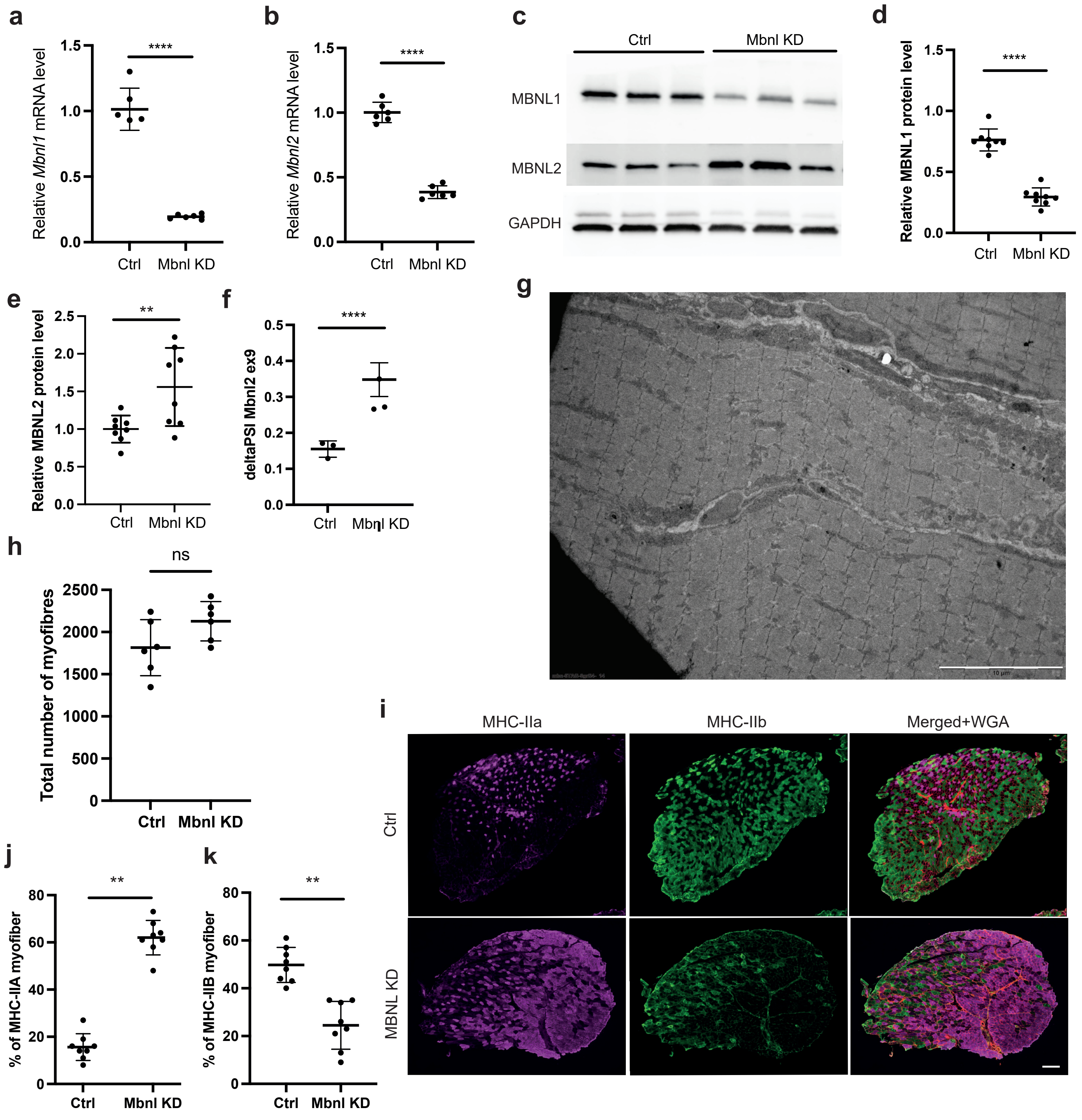

Supplementary Fig. S1: Validation of Mbnl1 and Mbnl2 knockdown and characterisation of tibialis anterior muscles following Mbnl knockdown in adult mice.

a, b Quantitative RT–PCR analysis of *Mbnl1* and *Mbnl2* mRNA levels normalized to *Rplp0*. **c–e** Analysis of MBNL1 and MBNL2 protein levels by western blot, normalized to GAPDH. **f** Percent Spliced In (PSI) of *Mbnl2* exon 9, determined by RNA-seq using rMATS analysis. **g** Transmission electron microscopy of Mbnl KD TA muscle showing fibre splitting. Scale bar: 10 µm. **h** Quantification of the total number of myofibres in control (Ctrl) and Mbnl KD TA muscles. **i** Representative immunofluorescence images of Ctrl and Mbnl KD TA transverse sections stained for myosin heavy chain (MyHC) type IIa (purple), type IIb (green), WGA (red), and Hoechst. Scale bar: 200 µm. **j, k** Quantitative RT–PCR analysis of MHC-IIa and MHC-IIb fibre proportions. For quantification on transverse muscle sections, at least 1,500 fibres were analysed. For panels a,b, and h: n = 6 mice per group; for panels d,e,j, and k: n = 8 mice per group; for panel f, n = 3 mice per group. Ctrl and Mbnl KD muscles were contralateral muscles from the same animals, and individual mice represent biological replicates.At least 6 mice were used; Ctrl and Mbnl KD muscles were contralateral muscles from the same animals. Data are presented as mean ± SD. Statistical analysis: paired t-test (a,b,d,e,h); Wilcoxon matched-pairs (j,k); for (e) see Source Data file; $^{**}p < 0.01$; $^{***}p < 0.001$; $^{****}p < 0.0001$. Exact *p* values and FDR values are provided in the Source Data file.

Supplementary Figure S2

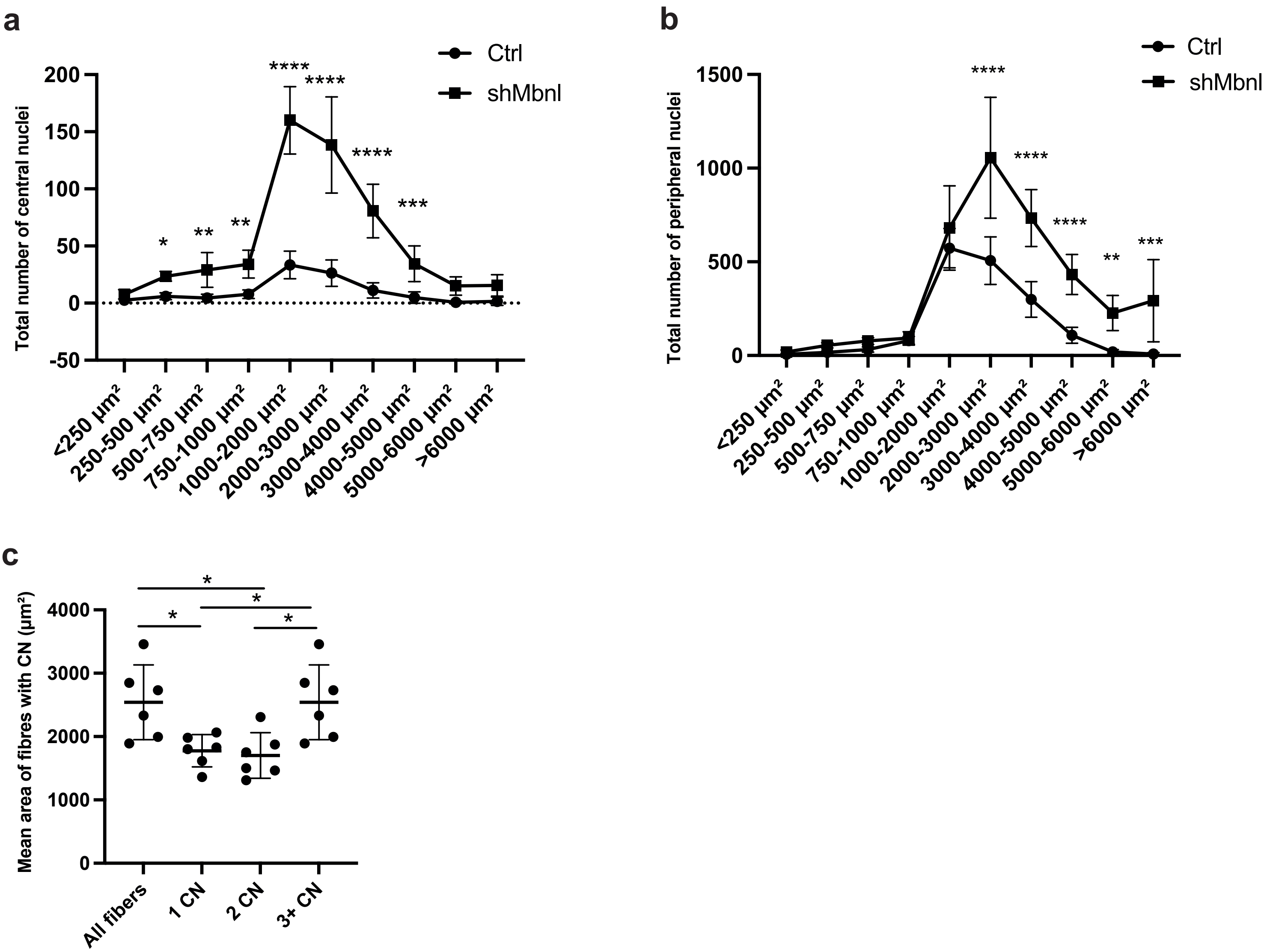

Supplementary Fig. S2: Histological analysis of tibialis anterior (TA) muscle following MBNL knockdown.
a Quantification of the total number and spatial distribution of centrally located nuclei across TA muscle cross-sections. **b** Quantification of the total number and spatial distribution of peripheral nuclei across TA muscle cross-sections. **c** Mean cross-sectional area of muscle fibres containing centrally located nuclei (μm²). For panels a–c, n = 6 mice per group; Ctrl and Mbnl KD muscles were contralateral muscles from the same animals, and individual mice represent biological replicates. For quantification on transverse muscle sections, at least 1,500 fibres were analysed. Data are presented as mean ± SD. Statistical significance was assessed using two-way ANOVA followed by Šídák's multiple-comparisons test for panels a and b, and one-way ANOVA followed by Tukey's multiple-comparisons test for panel c. Where indicated $*p < 0.05$, $**p < 0.01$, $***p < 0.001$ and $****p < 0.0001$. Exact adjusted p values are provided in the Source Data file.

Supplementary Figure S3

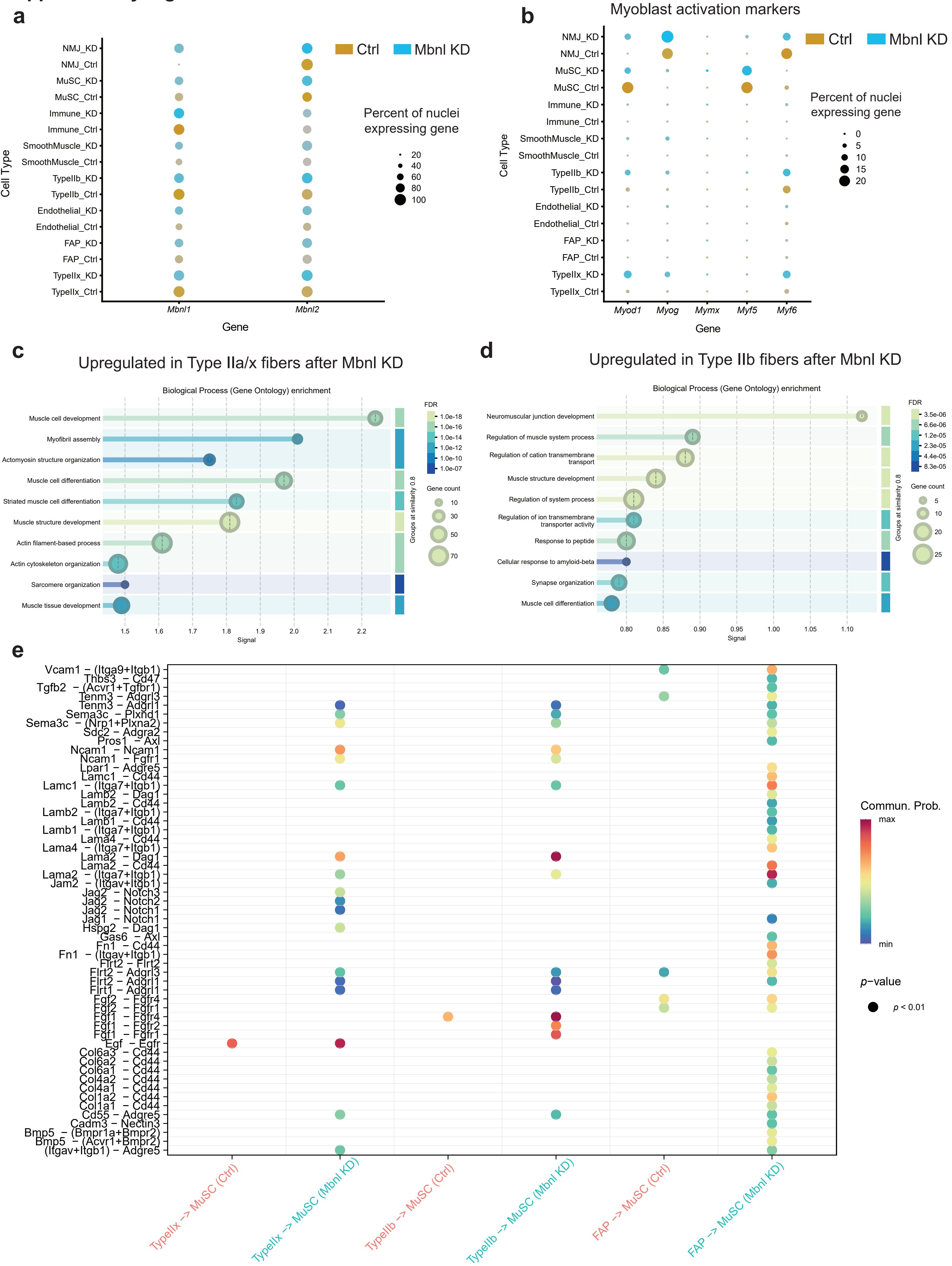

Supplementary Fig. S3: snRNA-seq characterisation of myonuclear transcriptional states following Mbnl knockdown.

a, b Dot plot for each cell type in Ctrl and Mbnl KD animals were the percentage of all nuclei in a given cell-type cluster expressing a specific gene is represented by the size of point and the intensity of coloring gives the average expression of those cells. Depicted are genes related to (A) Mbnl1/2, (B) myoblast activation. **c** Biological processes associated with upregulated genes in type IIa/x myonuclei after Mbnl KD. **d** Biological processes associated with upregulated genes in type IIb myonuclei after Mbnl KD. **e** CellChat analysis of ligand–receptor interactions directed toward muscle stem cells (MuSCs) from type IIx fibers, type IIb fibres, and fibro-adipogenic progenitors (FAPs) in Control (Ctrl) and MBNL knockdown (Mbnl KD) conditions. Dots represent communication probability and colour intensity indicates interaction strength. Only significant interactions ($p < 0.01$) are shown.

Supplementary Figure S4

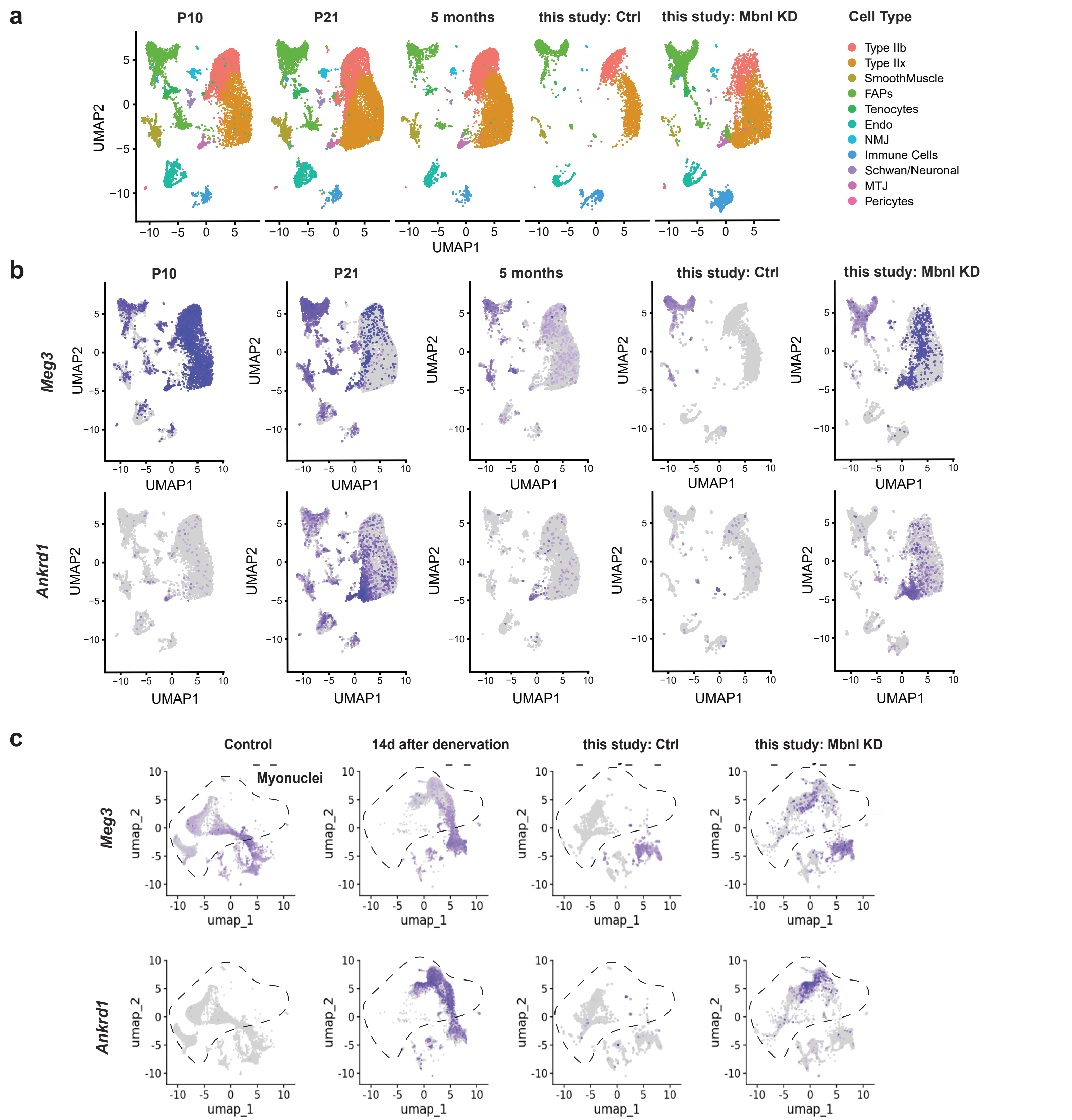

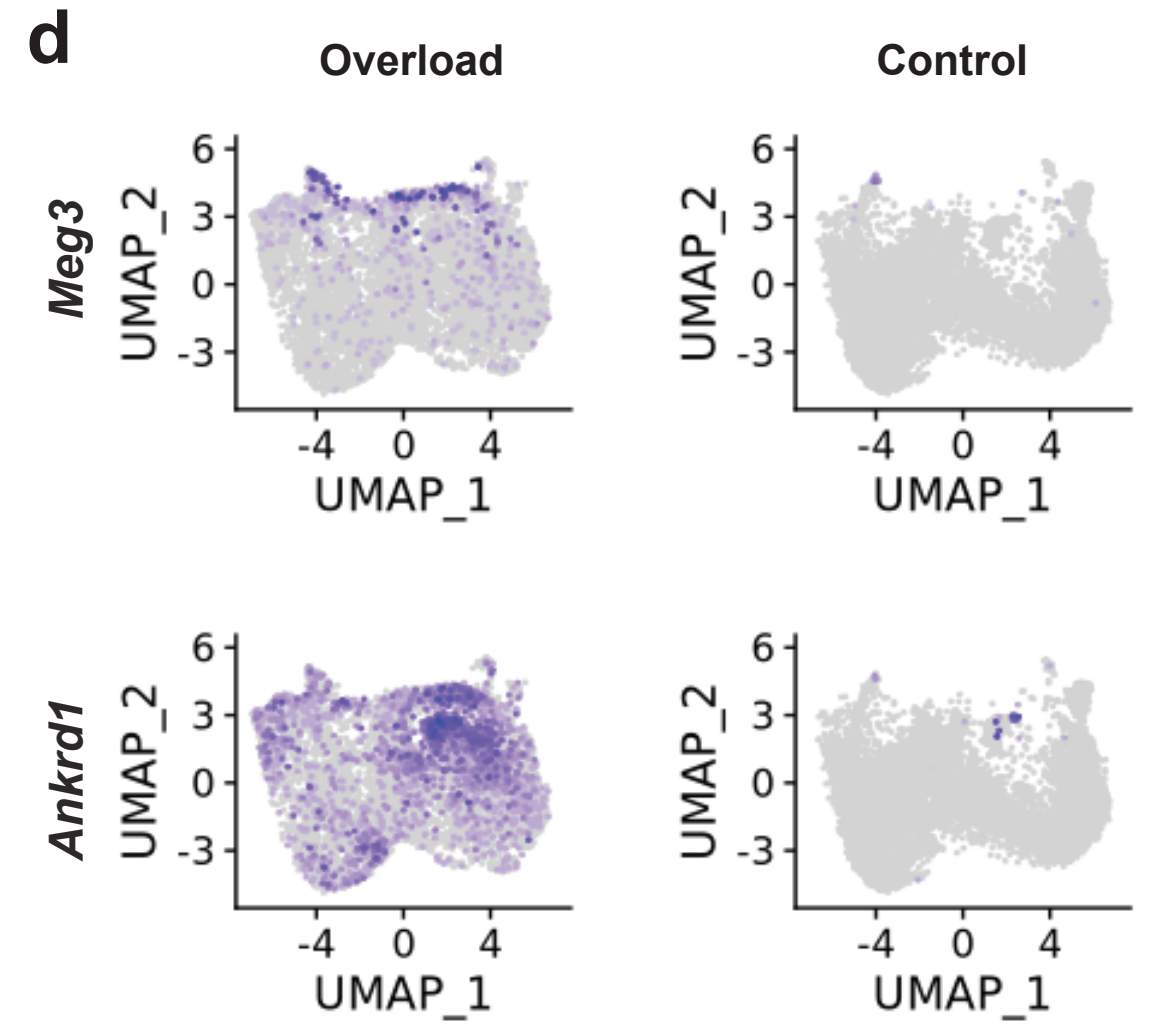

Supplementary Fig. S4: Comparison of *Meg3* and *Ankrd1* transcriptional populations with muscle developmental signatures, injury and overload.

a UMAP of the integrated data between the TA snRNA-seq from *Petrany et al.* (P10, P21 and 5-month) with Ctrl and Mbnl-KD TA muscles from this study. Each point represents a single-nucleus and is coloured according to cell-identity. **b** UMAPs as in (a) with nuclei coloured according to the expression level of *Ankrd1* and *Meg3*. Each point represents a single nucleus. **c** UMAP of the integrated dataset combining snRNA-seq from *Li et al.* (Control and 14 days post-denervation) with Ctrl and Mbnl-KD TA muscles from this study. **d** UMAP of integrated data from *Sun et al.*, comparing Control and muscle overload conditions. (c-d) In both panels, nuclei are coloured according to the expression level of *Ankrd1* and *Meg3*. Each point represents a single nucleus.

Supplementary Figure S5

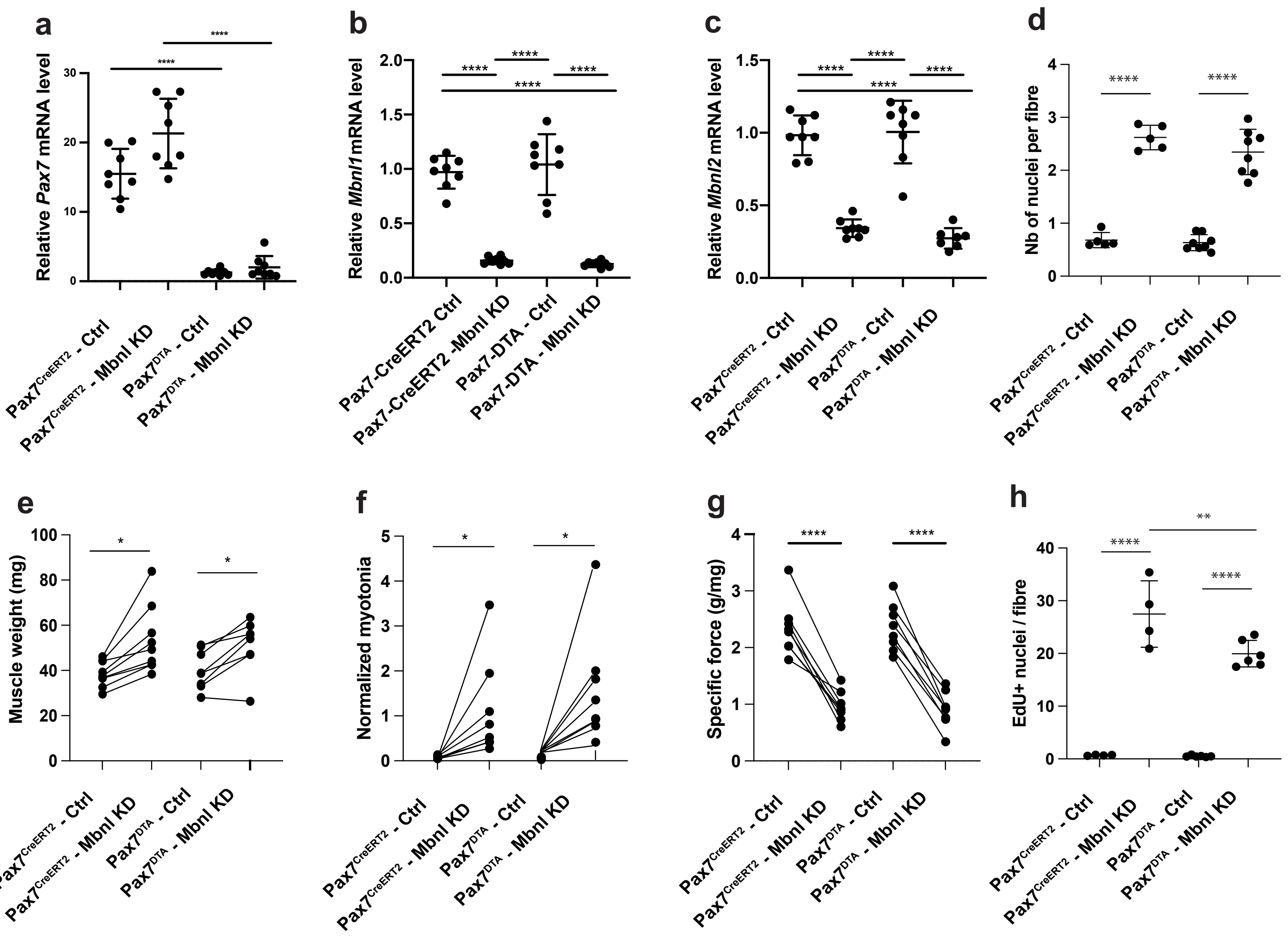

Supplementary Fig. S5: Muscle physiological parameters following Mbnl knockdown in Pax7^{DTA} mice.
a-c Quantitative RT–PCR analysis of *Pax7*, *Mbnl1* and *Mbnl2* mRNA levels normalized to *Rplp0*. **d** Number of nuclei per muscle fibre in transverse sections. **e** Comparison of TA muscle weight across groups. **f** Myotonia. **g** Specific force corresponding to maximal force normalized to muscle weight (g/mg). **h** Percentage of myonuclei positive for EdU in transverse sections. For panels, each dot represents one muscle (n). a,b,f,g: n = 8; c: n=8/8/8/7; d: n= 5/5/8/8; e: n=9/9/8/8; h: n=4/4/6/6. Pax7^{CreERT2} and Pax7^{DTA}, Ctrl and Mbnl KD muscles were contralateral muscles from the same animals where applicable, and individual mice represent biological replicates.Data are presented as mean ± SD. *p < 0.05, **p < 0.01, ***p < 0.001, ***p < 0.0001; one-way ANOVA followed by Tukey's multiple-comparisons test. Exact adjusted p values are provided in the Source Data file.

Supplementary Figure S6

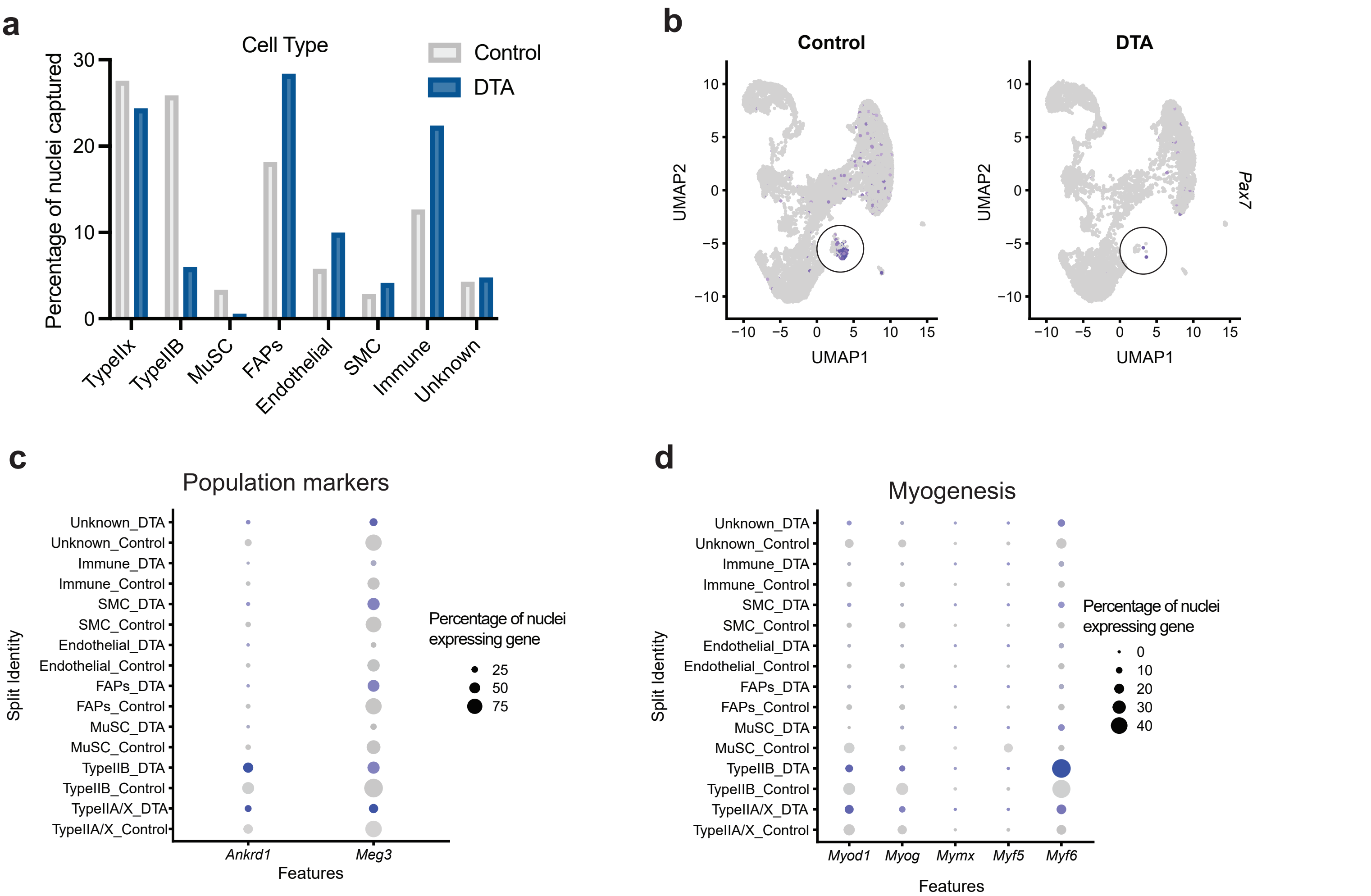

Supplementary Fig. S6: snRNA-seq characterization of myonuclear transcriptional states following Mbnl knockdown in Pax7^{DTA} mice.

a Percentage of captured nuclei that belong to each cell type. The targeted cell type, MuSCs are 3.3% in Pax7^{CreERT2} Mbnl KD TA and 0.5% in Pax7^{DTA} Mbnl KD TA. **b** Expression of *Pax7* transcript in Pax7^{CreERT2} TA and Pax7^{DTA} TA after Mbnl KD. **c, d** Dot plot for each cell type in Ctrl and Mbnl KD animals were the percentage of all nuclei in a given cell-type cluster expressing a specific gene is represented by the size of point and the intensity of coloring gives the average expression of those cells. Depicted are trancripts related to *Meg3* and *Ankrd1* transcripts (c) and myogenesis markers (d).

Supplementary Figure S7

	Total fiber number	Present	Absent	% (Present)	% (Absent)
**Ctrl_1**	3681			0	100
**Ctrl_2**	1325			0	100
**Ctrl_3**	6221			0	100
**DM1_1**	5933	224	5709	3.8	96.2
**DM1_2**	8778	898	7880	10.2	89.8
**DM1_3**	1911	1869	42	97.8	2.2

Supplementary Fig. S7: Numbers of fibres with central nuclei assessed by H&E staining.

Supplementary Figure S8

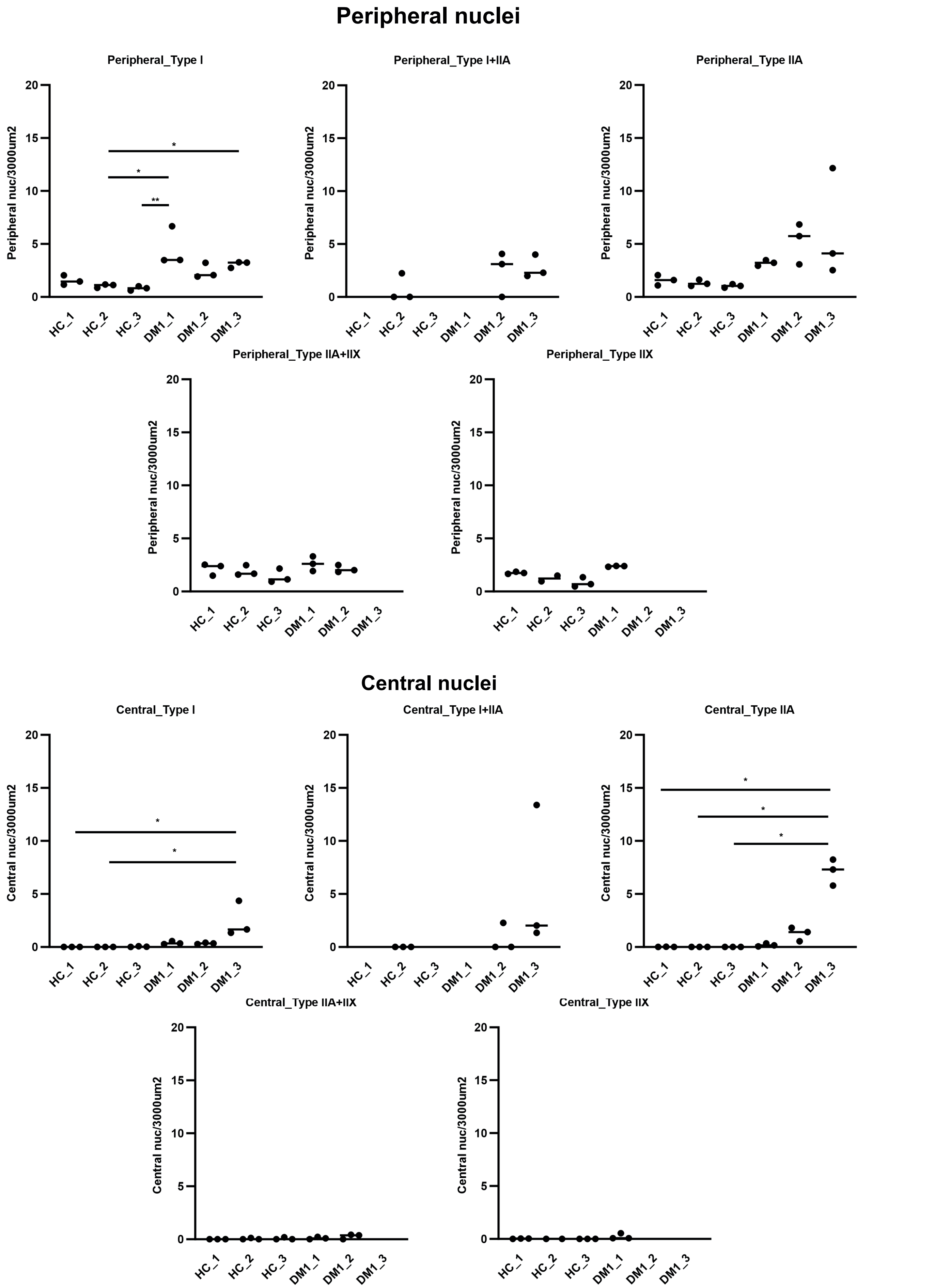

Supplementary Fig. S8: Nuclear counts normalised to tissue area with patient level statistics.
For the three independent fascicles of muscle fibres, all nuclei (central and peripheral) were counted for each individual muscle fibre type. For statistical analysis, the Kruskal–Wallis test was used. All pairwise group comparisons were based on unadjusted *P* values calculated using Dunn's rank-based test, and the false discovery rate was controlled separately for each outcome at $Q = 5\%$ using the two-stage linear step-up procedure of Benjamini, Krieger, and Yekutieli. *q* values are reported; comparisons with $q < 0.05$ were considered discoveries. *$q < 0.05$, **$q < 0.01$. Exact adjusted *p* values are provided in the Source Data file.

Supplementary Figure S9

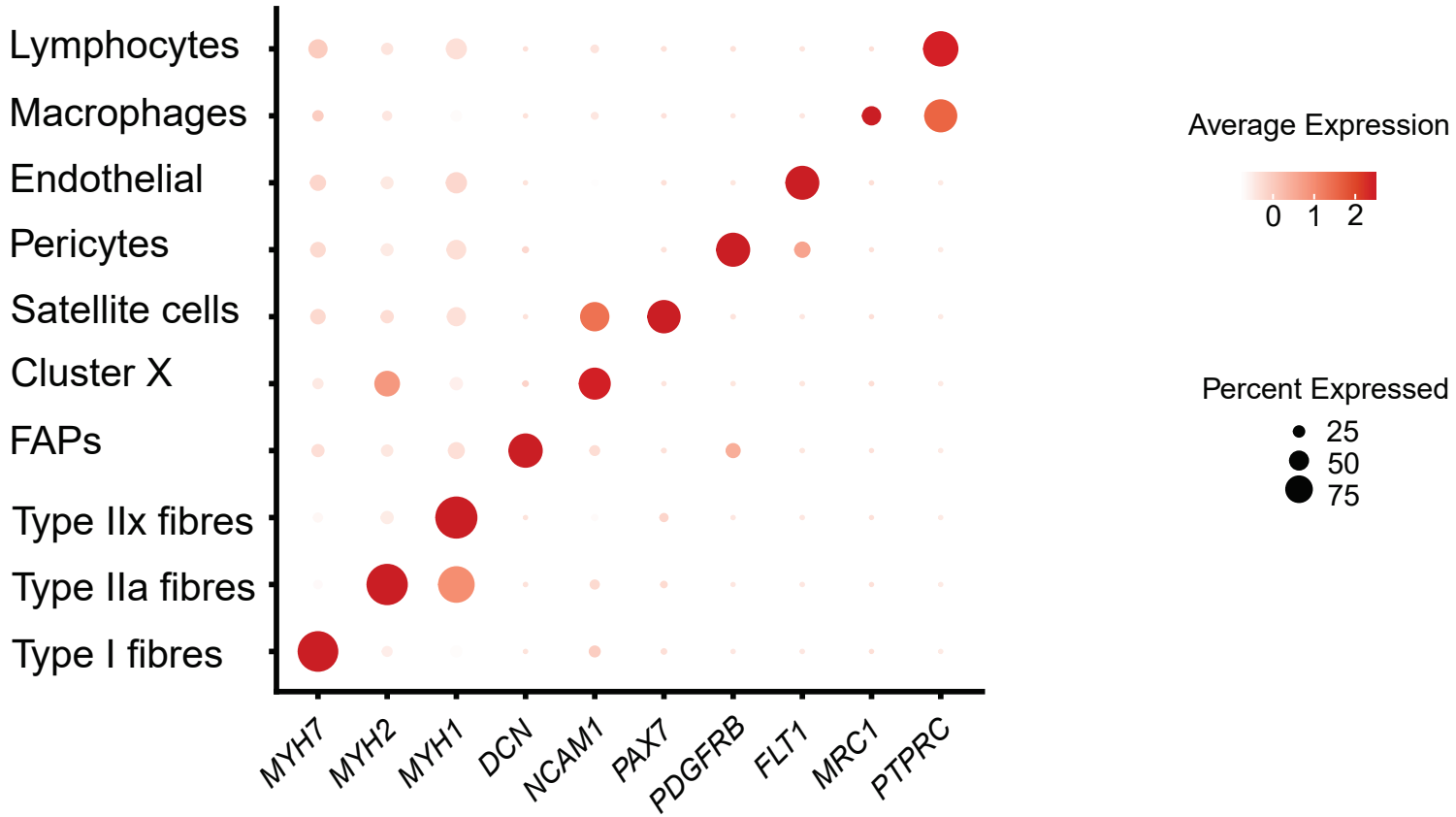

Supplementary Fig. S9: Cell-type marker gene expression. Dot plot showing the expression of canonical marker genes used for cell type annotation in the human snRNA-seq analysis. Dot size indicates the percentage of nuclei expressing the indicated gene, and colour intensity indicates average expression. n = 3 control donors and n = 3 DM1 patients were analysed; individual donors or patients represent biological replicates. This panel shows descriptive marker-gene expression used for cluster annotation; no statistical testing was performed for this visualisation. Values in the Source Data file.

Supplementary Figure S10

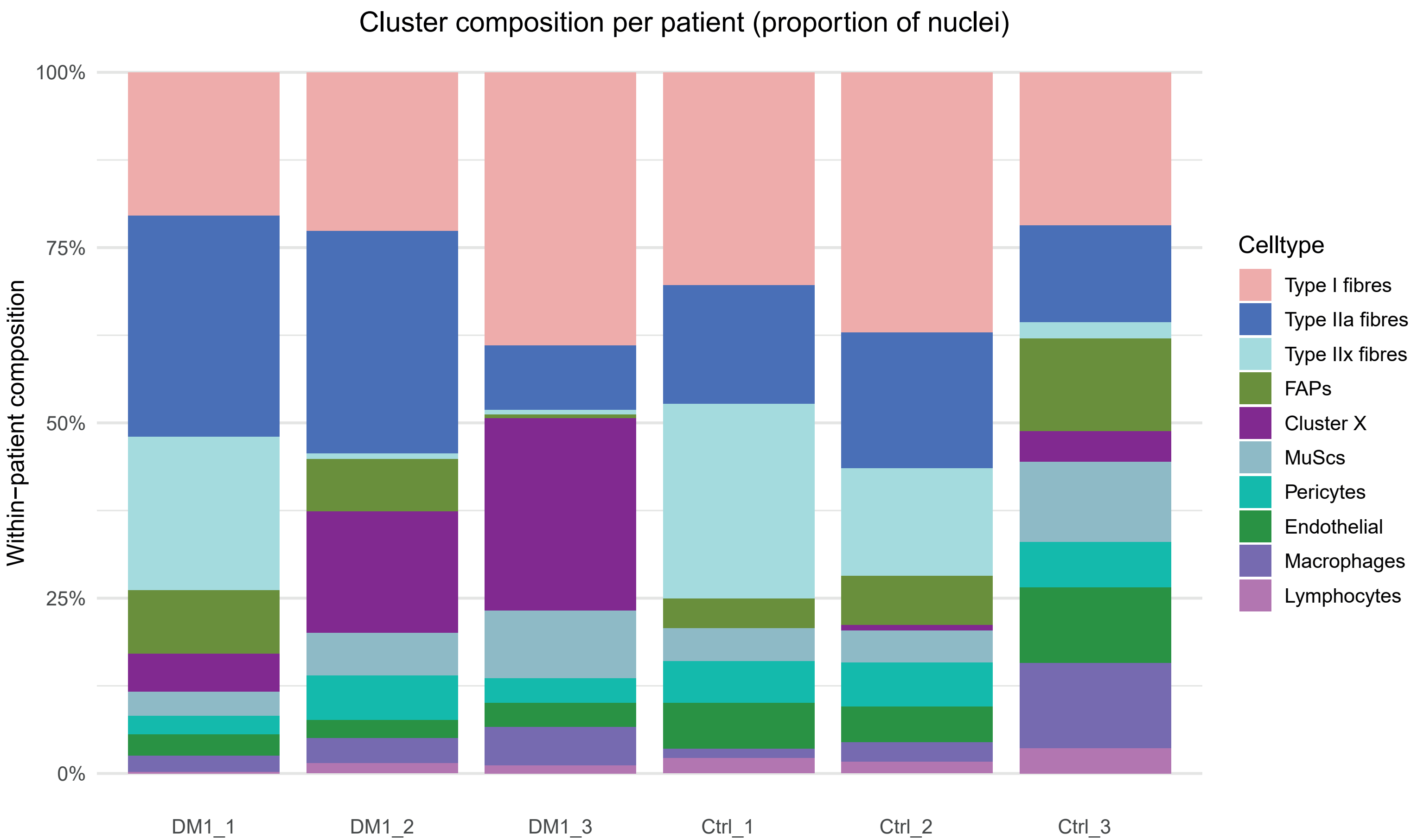

Supplementary Fig. S10: Donor-level proportions of nuclear clusters.
Barplots show, for each donor, the proportion of myonuclei assigned to each cluster. Colors correspond to the nuclear clusters defined in Fig. 5a–c. n = 3 control donors and n = 3 DM1 patients were analysed; individual donors or patients represent biological replicates. This panel shows donor-level cluster composition for descriptive comparison; no statistical testing was performed for this visualisation. Values in the Source Data file.

Supplementary Figure S11

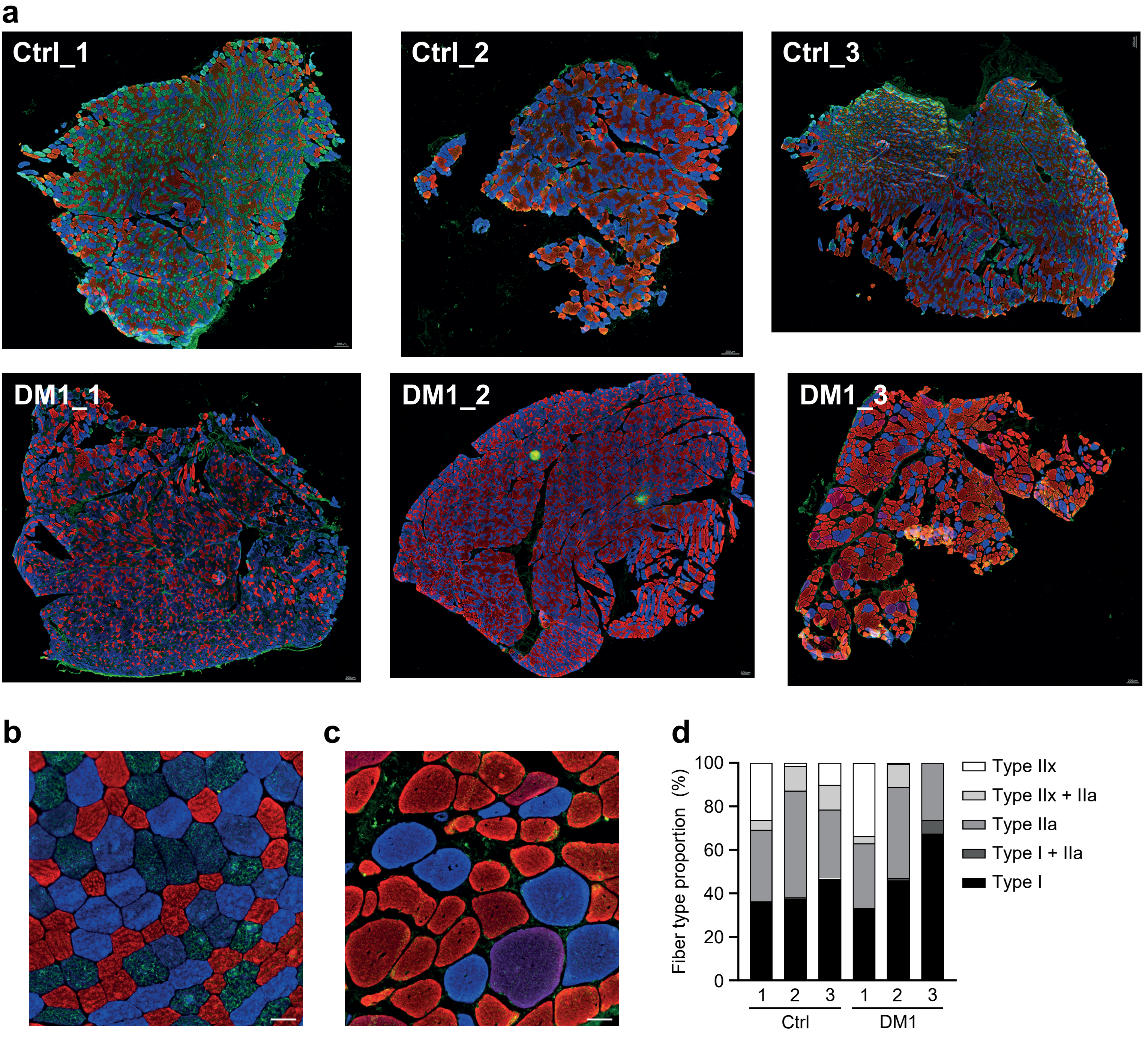

Supplementary Fig. S11: Fiber type distribution in Control and DM1 patient muscle specimens.
a Representative immunofluorescence images of control and DM1 patient muscles sections stained for myosin heavy chain (MyHC) Type I (red), Type IIa (blue), and Type IIx (green). Scale bar: 200 µm. **b** Higher magnification image of control (Ctrl_1) muscle section. Scale bar: 50 µm. **c** Higher magnification of DM1 (DM1_3) muscle section. Scale bar: 50 µm. **d** Quantification of fibre type proportion of control and DM1 patient muscle biopsies.

Supplementary Figure S12

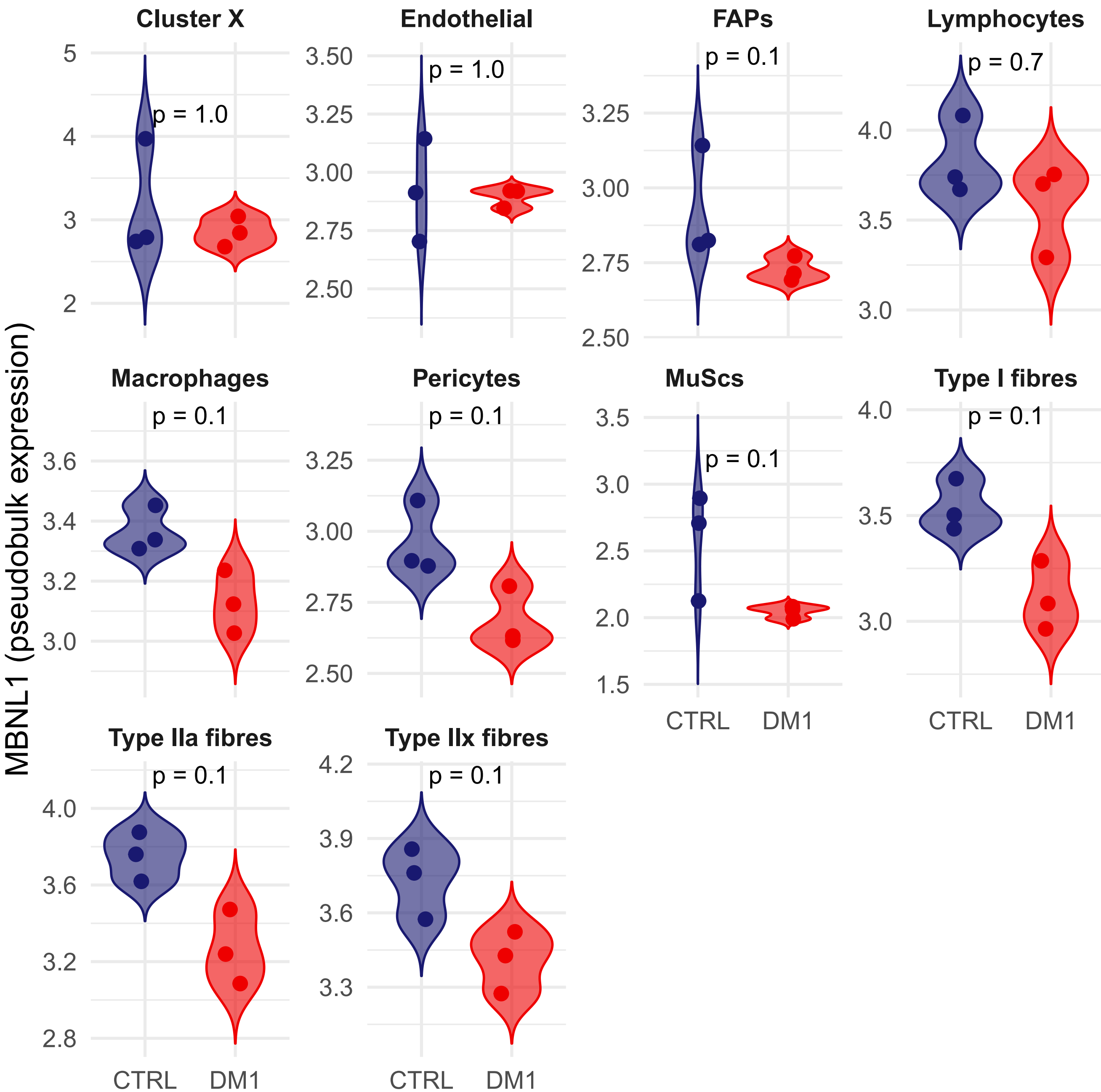

Supplementary Fig. S12: Pseudobulk expression of *MBNL1*. For each cluster, n = 3 control donors and n = 3 DM1 patients were analysed; individual donors or patients represent biological replicates. Individual points represent donor- or patient-level pseudobulk expression values within the indicated cluster. Violin plots show the distribution of donor- or patient-level values. Statistical significance was assessed using two-sided Wilcoxon rank-sum tests.

Supplementary Figure S13

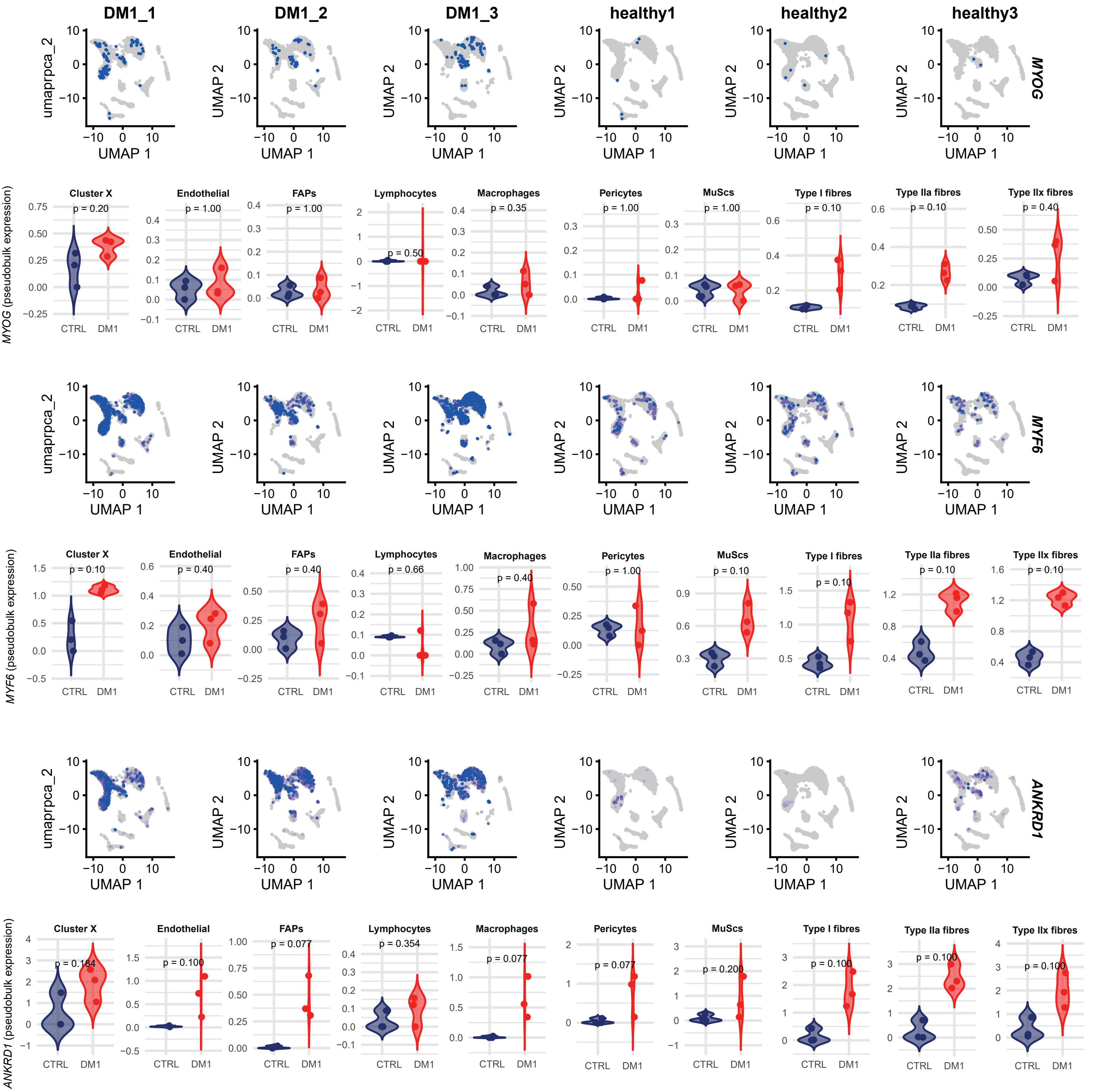

Supplementary Fig. S13: *MYOG*, *MYF6*, and *ANKRD1* expression across each individual human muscle specimen per cluster and as violin plot (pseudobulk expression). For each cluster, n = 3 control donors and n = 3 DM1 patients were analysed; individual donors or patients represent biological replicates. Individual points represent donor- or patient-level pseudobulk expression values within the indicated cluster. Violin plots show the distribution of donor- or patient-level values. Statistical significance was assessed using two-sided Wilcoxon rank-sum tests.

Supplementary Figure S14

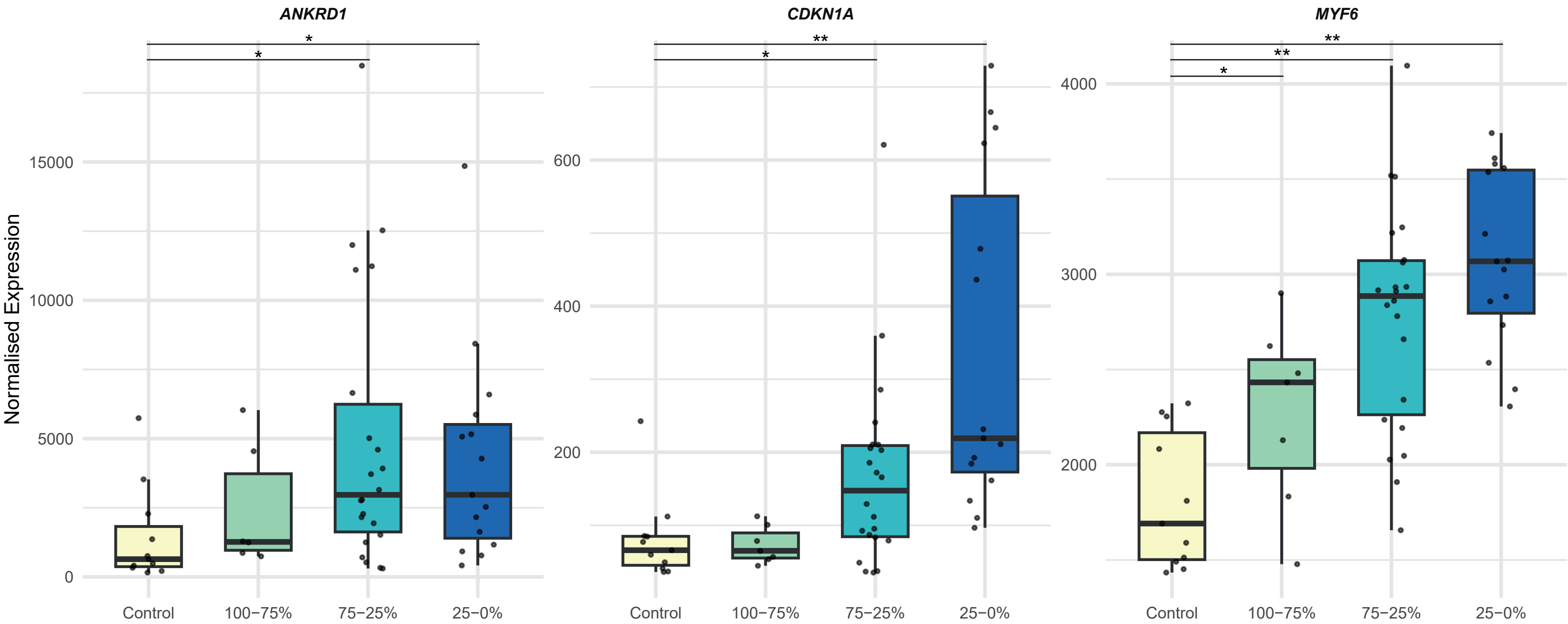

Supplementary Fig. S14: Expression of *ANKRD1*, *CDKN1A*, and *MYF6* genes analysed in a bulk RNA-seq dataset from unrelated DM1 patients (n = 9 controls, 43 patients). Patients were stratified by dorsiflexion strength into three clinical groups: 100–75% (mild/proto-DM1), 75–25% (classic DM1), and 25–0% (severe DM1). Individual donors or patients represent biological replicates. Box plots show the median centre line, the 25th and 75th percentiles as box limits, and whiskers extending to the most extreme data points within 1.5× the interquartile range from the box hinges. Differential expression was assessed using DESeq2. *P* values were calculated using the Wald test and adjusted for multiple testing using the Benjamini–Hochberg procedure. Where indicated, adjusted *$p < 0.05$, **$p < 0.01$.

Supplementary Figure S15

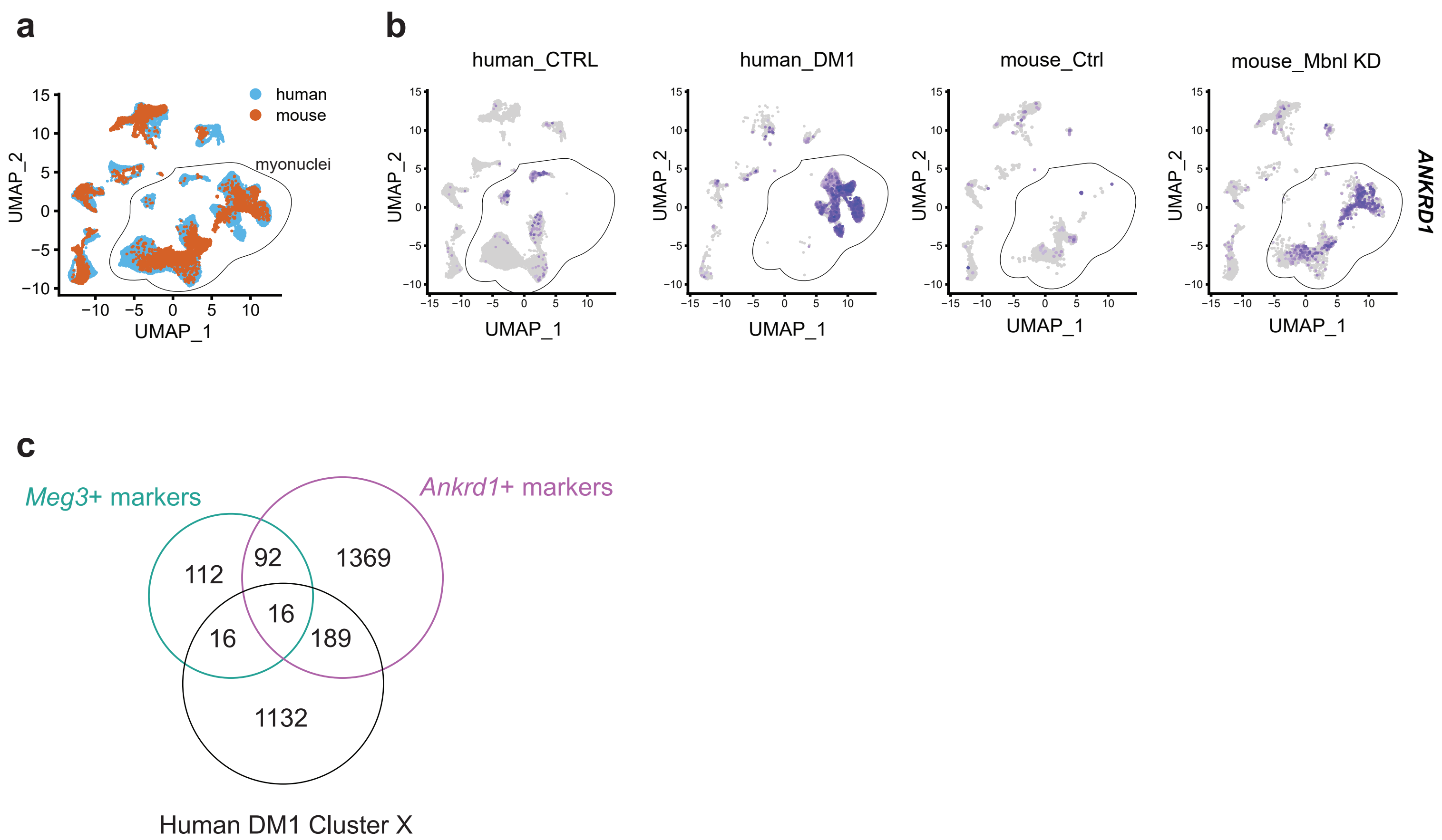

Supplementary Fig. S15: Overlap of human and mouse snRNA-seq data.

Supplementary Figure S16

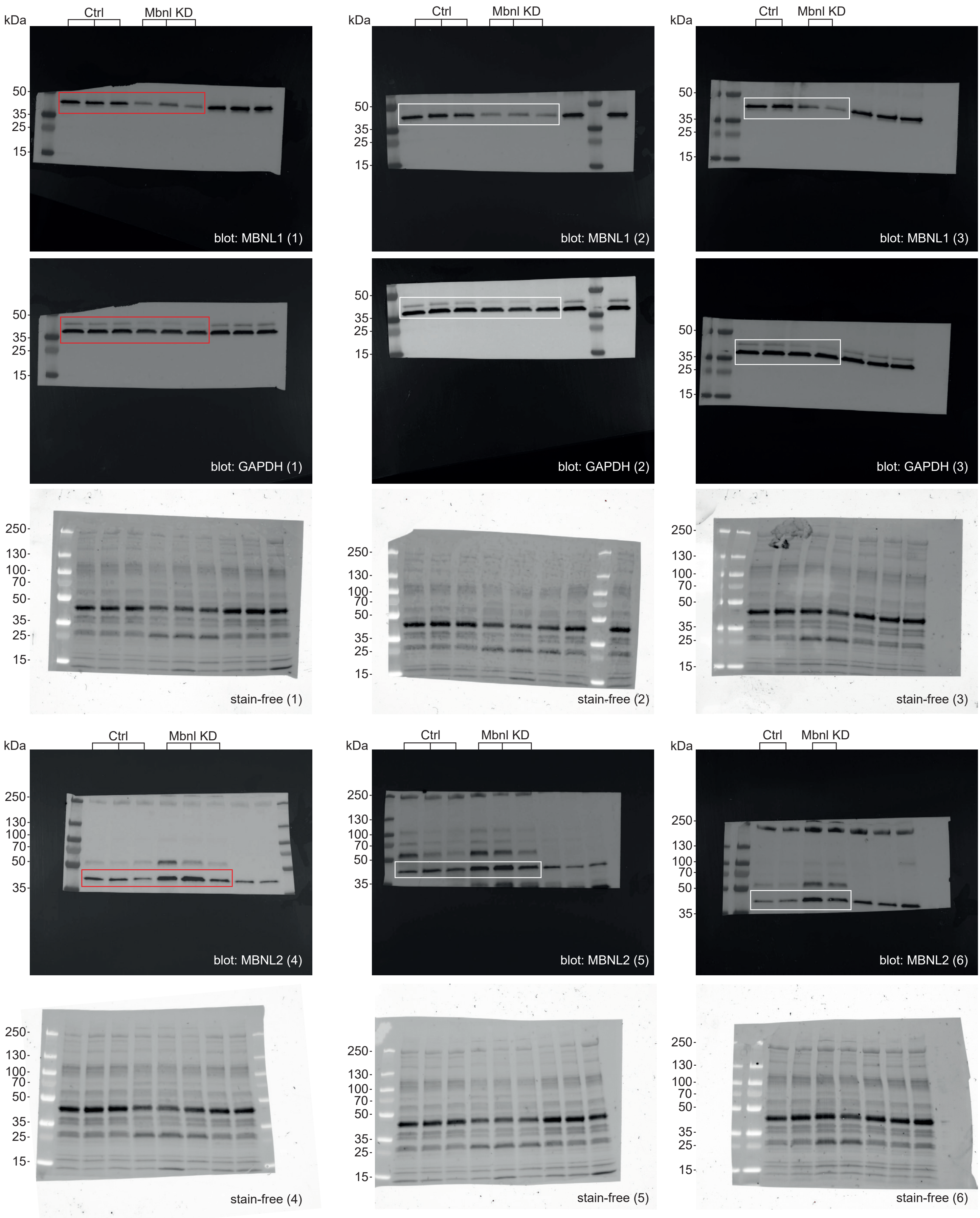

Supplementary Fig. S16: Uncropped scans of all blots in Supplementary Fig. S1c. Uncropped Western blot membranes corresponding to Supplementary Fig. S1c are shown. Red rectangles indicate the regions presented in Supplementary Fig. S1c. The regions used for quantification in the Source Data corresponding to Supplementary Figs. S1d and S1e are indicated by red and white rectangles. Molecular-weight markers are indicated in kDa, and sample identities are provided above the respective lanes. Samples from each independent muscle were loaded in duplicate on two separate stain-free gels (Bio-Rad). After transfer, equal protein loading was verified by stain-free imaging. Membrane 1 was first incubated with rabbit anti-MBNL1 antibody (1:1,000). Following detection, the membrane was stripped, washed and subsequently incubated with rabbit anti-GAPDH antibody (1:5,000). Membrane 2 was incubated with mouse anti-MBNL2 antibody (1:200). Immunoreactive bands were detected using Immobilon Western HRP substrate and imaged using a ChemiDoc Touch MP Imaging System (Bio-Rad).
