# Supplementary material for: MBNL depletion drives stem cell fusion and immature myonuclear states in myotonic dystrophy type 1
Source: Nat Commun. 2026 Aug 6;17:9476. doi: 10.1038/s41467-026-76476-6 (PMC13542076; doi:10.1038/s41467-026-76476-6)
Supplement: Supplementary file 2 [file 41467_2026_76476_MOESM2_ESM.pdf]

Description of Additional Supplementary Files

Supplementary Data 1 : RNAseq splicing analysis on Mbnl KD and Ctrl TA muscles (rmats analysis)

Supplementary Data 2 : RNAseq gene expression analysis on Mbnl KD and Ctrl TA muscles (DeqSeq2 analysis)

Supplementary Data 3 : snRNA-seq–defined nuclei population percentages in Pax7^{DTA} TA muscles

Supplementary Data 4 : snRNAseq profiling of type IIx myonuclear transcripts expression in Ctrl and Mbnl KD TA muscles

Supplementary Data 5 : snRNAseq profiling of type IIb myonuclear transcripts expression in Ctrl and Mbnl KD TA muscles

Supplementary Data 6 : Marker genes of the *Meg3*$^+$ myonuclear population in Mbnl KD TA muscles

Supplementary Data 7 : Marker genes of the *Ankrd1*$^+$ myonuclear population in Mbnl KD TA muscles

Supplementary Data 8: 933 genes significantly differentially expressed in Cluster X compared to other myonuclear clusters

Supplementary Data 9 : Marker genes shared between mouse *Meg3*$^+$ and *Ankrd1*$^+$ myonuclear populations and human cluster X identified by snRNAseq

Supplementary Data 10: Guide RNA sequences for *DMPK*

Supplementary Data 11: Antibodies used in this study
