# Supplementary material for: MBNL depletion drives stem cell fusion and immature myonuclear states in myotonic dystrophy type 1
Source: Nat Commun. 2026 Aug 6;17:9476. doi: 10.1038/s41467-026-76476-6 (PMC13542076; doi:10.1038/s41467-026-76476-6)
Supplement: Supplementary file 3 [file 41467_2026_76476_MOESM4_ESM.pdf]

nature portfolio

Corresponding author(s): Prof. Dr. Benedikt Schoser
Prof. Dr. Denis Furling
Dr. Shinichiro Hayashi

Last updated by author(s): Jun 24, 2026

Reporting Summary

Nature Portfolio wishes to improve the reproducibility of the work that we publish. This form provides structure for consistency and transparency in reporting. For further information on Nature Portfolio policies, see our Editorial Policies and the Editorial Policy Checklist.

Statistics

For all statistical analyses, confirm that the following items are present in the figure legend, table legend, main text, or Methods section.

n/a	Confirmed	
☐	☒	The exact sample size (*n*) for each experimental group/condition, given as a discrete number and unit of measurement
☐	☒	A statement on whether measurements were taken from distinct samples or whether the same sample was measured repeatedly
☐	☒	The statistical test(s) used AND whether they are one- or two-sided<br>*Only common tests should be described solely by name; describe more complex techniques in the Methods section.*
☒	☐	A description of all covariates tested
☐	☒	A description of any assumptions or corrections, such as tests of normality and adjustment for multiple comparisons
☐	☒	A full description of the statistical parameters including central tendency (e.g. means) or other basic estimates (e.g. regression coefficient) AND variation (e.g. standard deviation) or associated estimates of uncertainty (e.g. confidence intervals)
☐	☒	For null hypothesis testing, the test statistic (e.g. *F*, *t*, *r*) with confidence intervals, effect sizes, degrees of freedom and *P* value noted<br>*Give P values as exact values whenever suitable.*
☒	☐	For Bayesian analysis, information on the choice of priors and Markov chain Monte Carlo settings
☐	☒	For hierarchical and complex designs, identification of the appropriate level for tests and full reporting of outcomes
☒	☐	Estimates of effect sizes (e.g. Cohen's *d*, Pearson's *r*), indicating how they were calculated

Our web collection on statistics for biologists contains articles on many of the points above.

Software and code

Policy information about availability of computer code

Data collection	Data collection was performed using standard commercial and open-source software provided by instrument manufacturers. Single-nucleus RNA sequencing libraries were generated using the Chromium platform (10x Genomics) and sequenced on an Illumina sequencing system using Illumina control software. Raw sequencing data were processed using Cell Ranger (10x Genomics, v7.1.0) for demultiplexing, alignment to the reference genome, and generation of gene–barcode count matrices. Microscopy images were acquired using Zeiss Zen software (Zeiss, v3.6), and muscle physiology measurements were collected using Aurora Scientific Dynamic Muscle Control software (Aurora Scientific). No custom software was used for data collection.
Data analysis	Data analysis was performed using established open-source and commercial software. Single-nucleus RNA sequencing data were analysed using R (v4.3.1) with Seurat (v4.3.0) for quality control, normalization, dimensionality reduction, clustering, and differential expression analysis. Gene ontology enrichment analysis was performed using Enrichr and clusterProfiler (v4.6.2). Statistical analyses were performed using GraphPad Prism (v9.5.1) and R (v4.3.1). Image quantification was performed using Fiji/ImageJ (v2.9.0). Figure preparation was performed using Adobe Illustrator (v28.0). No custom code was developed for this study.

For manuscripts utilizing custom algorithms or software that are central to the research but not yet described in published literature, software must be made available to editors and reviewers. We strongly encourage code deposition in a community repository (e.g. GitHub). See the Nature Portfolio guidelines for submitting code & software for further information.

Data

Policy information about availability of data

All manuscripts must include a data availability statement. This statement should provide the following information, where applicable:

- Accession codes, unique identifiers, or web links for publicly available datasets
- A description of any restrictions on data availability
- For clinical datasets or third party data, please ensure that the statement adheres to our policy

The single-nucleus RNA-sequencing and bulk messenger RNA-sequencing data generated in this study have been deposited in the Gene Expression Omnibus (GEO) database under accession codes GSE262646 (mouse single-nucleus RNA-sequencing after MBNL knockdown), GSE335902 (mouse single-nucleus RNA-sequencing after Pax7-DTA-mediated muscle stem cell ablation), and GSE335961 (mouse bulk messenger RNA-sequencing after MBNL knockdown). Processed sequencing data generated in this study are provided in the Supplementary Information, Supplementary Tables and Source Data files. Source data are provided with this paper. Human clinical metadata are not publicly available at individual level because of participant privacy and ethical restrictions; only aggregated or pseudonymised information required to interpret the study is provided in the manuscript and associated files. No custom code requiring separate deposition was generated for this study.

Research involving human participants, their data, or biological material

Policy information about studies with human participants or human data. See also policy information about sex, gender (identity/presentation), and sexual orientation and race, ethnicity and racism.

Reporting on sex and gender	Sex was considered in the study design. Human muscle biopsy samples were obtained from adult donors of both sexes, and mouse experiments included both male and female animals. Sex was determined based on clinical records for human participants and anatomical characteristics for mice. No sex-specific exclusion criteria were applied. The study was not designed or powered to detect sex-dependent differences, and analyses were therefore not stratified by sex. The findings are expected to apply to both sexes, although sex-specific effects cannot be formally excluded.
Reporting on race, ethnicity, or other socially relevant groupings	Race, ethnicity, and other socially relevant groupings were not considered in the study design or analysis. Human muscle biopsy samples were obtained based on clinical diagnosis and availability through established biobank and clinical protocols. Information on race and ethnicity was not collected or used, and no analyses were performed based on these variables. Therefore, the findings are not intended to address differences related to race, ethnicity, or other socially defined groupings.
Population characteristics	Human skeletal muscle biopsy samples were obtained from adult patients with genetically confirmed myotonic dystrophy type 1 (DM1) and from control donors without neuromuscular disease. Clinical information, including age, sex, muscle biopsy site, CTG repeat size, and clinical severity measures, was recorded. Both male and female participants were included. Biopsies were obtained from clinically relevant skeletal muscles, including biceps brachii and quadriceps femoris. Control samples were obtained from individuals without neuromuscular pathology. No participants were excluded based on sex.
Recruitment	DM1 muscle biopsy samples were obtained from adult patients with genetically confirmed myotonic dystrophy type 1 recruited through neuromuscular clinics at participating institutions. Control samples were obtained from individuals without neuromuscular disease through diagnostic procedures or institutional biobanks. All samples were collected with informed consent under protocols approved by the appropriate institutional ethics committees.
Ethics oversight	Written informed consent was obtained from all participants prior to inclusion in the study. Human muscle biopsy collection and use were approved by the Ethics Review Board of the National Center of Neurology and Psychiatry (NCNP), Japan (approval number B2024-130), and conducted in accordance with the Declaration of Helsinki and applicable institutional and national guidelines.

Note that full information on the approval of the study protocol must also be provided in the manuscript.

Field-specific reporting

Please select the one below that is the best fit for your research. If you are not sure, read the appropriate sections before making your selection.

☒ Life sciences ☐ Behavioural & social sciences ☐ Ecological, evolutionary & environmental sciences

For a reference copy of the document with all sections, see nature.com/documents/nr-reporting-summary-flat.pdf

Life sciences study design

All studies must disclose on these points even when the disclosure is negative.

Sample size	Sample sizes were determined based on established standards in the neuromuscular field and on prior experience with the experimental models and readouts used in this study. Sample sizes were sufficient to detect biologically meaningful differences and were consistent with those reported in previous studies using comparable AAV-mediated knockdown models, muscle histology, physiological measurements, and single-nucleus RNA sequencing analyses. For mouse experiments, group sizes typically ranged from 3 to 8 animals per condition, depending on the assay. For human muscle biopsies, sample availability is inherently limited due to the rarity of the disease and ethical considerations; all available and suitable samples meeting inclusion criteria were included. No formal statistical method was used to predetermine sample size.
Data exclusions	No data were excluded from the analyses unless predefined technical quality control criteria were not met. For single-nucleus RNA

sequencing, nuclei with low transcript counts, high mitochondrial RNA content, or other indicators of poor quality were excluded according to standard filtering procedures described in the Methods section. For histological, physiological, and molecular analyses, all samples that met predefined experimental and technical quality criteria were included. No exclusions were made based on experimental outcome.

Replication	All key findings were reproduced in independent biological samples. Mouse experiments were performed in multiple independent animals per condition, and results were consistent across biological replicates. Human single-nucleus RNA sequencing analyses were conducted across independent donors, and observed transcriptional patterns were reproducible across individuals. Histological, molecular, and functional analyses were independently repeated with similar results. No attempt at replication failed.
Randomization	Animals were randomly assigned to experimental groups where applicable. For AAV-mediated knockdown experiments, one limb was injected with control AAV and the contralateral limb with Mbnl knockdown AAV, allowing paired comparisons within the same animal and minimizing inter-individual variability. Human samples were obtained from independent donors, and randomization was not applicable.
Blinding	Investigators were blinded to genotype and experimental condition during histological and imaging quantifications. Analyses were performed using coded samples to ensure unbiased assessment. Human samples were analyzed using anonymized identifiers.

Reporting for specific materials, systems and methods

We require information from authors about some types of materials, experimental systems and methods used in many studies. Here, indicate whether each material, system or method listed is relevant to your study. If you are not sure if a list item applies to your research, read the appropriate section before selecting a response.

Materials & experimental systems

n/a	Involved in the study
☐	☒ Antibodies
☒	☐ Eukaryotic cell lines
☒	☐ Palaeontology and archaeology
☐	☒ Animals and other organisms
☒	☐ Clinical data
☒	☐ Dual use research of concern
☒	☐ Plants

Methods

n/a	Involved in the study
☒	☐ ChIP-seq
☒	☐ Flow cytometry
☒	☐ MRI-based neuroimaging

Antibodies

Antibodies used	The following primary antibodies were used in this study:<br><br>Laminin (supplier Abcam; AB11575; lot-no.106 3229-1)<br>Cav3 (supplier Santa Cruz Biothechnology; catalog no. sc-7665; lot-no H290)<br>MBNL1 (supplier gift from Pr. Thornton; clone A2764)<br>MBNL2 (supplier gift from Pr. Morris; clone 3B4)<br>PAX7 (supplier Santa Cruz Biothechnology; catalog no. sc-81648; clone PAX7)<br>Ki67 (supplier abcam; catalog no. ab15580; lot-no GR3285096)<br>p21 (supplier Santa Cruz Biothechnology; catalog no. sc-397; clone XX; lot-no L2013)<br>MHC-IIa (supplier Developmental Studies Hybridoma Bank; catalog no. SC-71; lot-no 04-27-23)<br>MHC-IIb (supplier Developmental Studies Hybridoma Bank; catalog no. BF-F3; lot-no 7-13-23)<br>GAPDH (supplier Santa-Cruz; catalog no. sc-25778; lot-no C2103)<br>MHC-I (supplier Developmental Studies Hybridoma Bank; catalog no. BA-D5; clone BA-D5)<br>MHC-IIa (supplier Developmental Studies Hybridoma Bank; catalog no. SC-71; clone SC-71)<br>MHC-IIx (supplier Developmental Studies Hybridoma Bank; catalog no. 6H1; clone 6H1)
Validation	A2764 and MB2a were validated and described in Lin et al (HMG, 2006) and Holt et al (Am. J. Pathol., 2009), respectively. For commercial antibodies, validation statements are on manufacturer's website.

Animals and other research organisms

Policy information about studies involving animals; ARRIVE guidelines recommended for reporting animal research, and Sex and Gender in Research

Laboratory animals	All animal experiments were performed in mice (Mus musculus). Wild-type FVB mice were used for AAV-mediated Mbnl knockdown experiments. For muscle stem cell lineage tracing and ablation experiments, Pax7CreERT2 and Pax7CreERT2; Rosa26DTA transgenic mice on a C57BL/6J background were used. Adult mice aged 8–10 weeks at the time of AAV injection were used in all experiments. Both male and female mice were included.
Wild animals	The study did not involve wild animals.

Reporting on sex	Both male and female mice were included in this study. The study was not designed or powered to detect sex-specific differences, and data were therefore analyzed with sexes combined. The findings are expected to apply to both male and female animals.
Field-collected samples	The study did not involve samples collected from the field.
Ethics oversight	All mouse procedures were conducted in accordance with institutional and national ethical guidelines and were approved under protocols APAFIS#25244-2020042716304109v6 and APAFIS#37309-202205101648455v5 by the Ethical Committee on Animal Resources at the Centre d'Expérimentation Fonctionnelle, Pitié-Salpêtrière animal facility (Paris, France).

Note that full information on the approval of the study protocol must also be provided in the manuscript.

Plants

Seed stocks	n/a
Novel plant genotypes	n/a
Authentication	n/a
