# Supplementary material for: MBNL depletion drives stem cell fusion and immature myonuclear states in myotonic dystrophy type 1
Source: Nat Commun. 2026 Aug 6;17:9476. doi: 10.1038/s41467-026-76476-6 (PMC13542076; doi:10.1038/s41467-026-76476-6)
Supplement: Supplementary file 4 [file 41467_2026_76476_MOESM5_ESM.pdf]

nature portfolio

Peer Review File

MBNL Depletion Drives Stem Cell Fusion and Immature Myonuclear States in Myotonic Dystrophy Type 1

Corresponding Author: Professor Benedikt Schoser

This file contains all reviewer reports in order by version, followed by all author rebuttals in order by version.

Version 0:

Reviewer comments:

Reviewer #1

(Remarks to the Author)
In this study, the authors integrated Mbnl knockdown mouse models, including MuSC-depleted mice, together with human samples to elucidate the mechanisms underlying the increase in total and central myonuclei, a hallmark of DM1 pathology. Moreover, they identified a CUG-repeat–dependent Cluster X in DM1 which is characterized by an immature transcriptional profile, elevated DMPK expression, and increased p21 expression. Overall, this work is highly interesting; however, the conclusions drawn from the Pax7-DTA model remain limited, and more detailed analyses are required to clarify the contribution of MuSCs to the pathophysiological features of DM1. A critical issue is whether MuSC activation, proliferation, and fusion occur independently of muscle degeneration and regeneration.

Major points
1. In the abstract and discussion, the authors state that 'central myonuclei accumulation independent of degeneration'. However, even if overt signs of degeneration are absent in the analyzed samples, degeneration may have occurred at an earlier stage. In Figure 1, it is essential to clarify whether central nuclei arise primarily from MuSC–myofiber fusion, from regeneration following degeneration, or through both mechanisms.
Compared to other muscular dystrophies characterized by active cycles of regeneration and degeneration, the absence of marked variation in myofiber size (Fig. 1E) supports the notion of limited regenerative and degenerative activity in this model. Analyzing the relationship between myonuclear number and myofiber size would further strengthen the authors' interpretation. If the authors intend to make this claim, it is essential to clearly explain that the increased frequency of centrally located nuclei is primarily attributable to MuSC-mediated fusion with pre-existing myofibers, supported by evidence including human studies. Additionally, the results of myofiber number should be included in Figure 1.

2. In this study, the authors used the Spc5.12 promoter instead of Mhck7. The direct effect of AAV-mediated silencing on MuSCs needs to be clarified, even though the expression level of Mbnl1 may be very low. Alternatively, based on past reports, etc., please state that there is no direct effect of Mbnl silencing by AAV on MuSC.

3. It remains unclear whether the reactivation of Meg3 and Ankrd1 is restricted to MuSC-derived nuclei or also occurs in pre-existing myonuclei. This issue could be addressed by analyzing the Pax7-DTA model.

4. Figure 3: The Pax7-DTA results clearly demonstrate that majority of central nuclei in DM1 originate from MuSCs. Were the other central myonuclei in Pax7-DTA mice originated from pre-existing myonuclei or from remained MuSCs? Additionally, the authors need to analyze the total number of myonuclei and skeletal muscle mass in Pax7-DTA model as shown in Figure 1A and 1G. If a significant increase in peripheral myonuclei is observed in Pax7-DTA, this would strongly suggest division of pre-existing myonuclei. By investigating the number of myofibers and muscle mass, it will become clear whether these are due to myofiber splitting or to new myofiber formed via MuSC.

5. Figure 4J: The detection of Ki67+ myonuclei is intriguing. Are Ki67+ central and peripheral myonuclei observed in the mouse model as observed in human samples? If so, the authors can address whether these nuclei originated from MuSCs or not using the Pax7-DTA model. The authors need to clarify the source of Ki67+ myonuclei and also examine the contribution of pre-existing myonuclei. EdU-injection experiment also should be performed whether pre-existing myonuclei uptake EdU by the loss of Mbn1.

6. Figure 4: In relation to Comment 1, it is essential to confirm that this MuSC proliferation is not simply a consequence of degeneration, but rather results from the disruption of quiescence signaling in MuSCs, caused directly or indirectly by MBNL deficiency in myofibers. Based on the snRNA-seq data, the authors should discuss potential candidate factors that may impair MuSC quiescence or promote their activation.

Minor comments:
1. Since both muscle fiber and myofiber are used, the terminology should be standardized as myofiber throughout the manuscript.

2. Line 34; The term “repair” generally implies the restoration process following deterioration or damage. If the authors intend to argue that MuSC activation and proliferation occur independently of degeneration, this reviewer believes that the term “repair” should be avoided, as it may confuse readers.

3. Lines 59-61; ‘Indeed, MBNL proteins bind with high affinity to YGCY motifs and, therefore, to expanded CUG repeats in proportion to the size of this expansion.’

It would aid reader comprehension to add the clarification: “Y = pyrimidine.”

4. Lines 81-83; MuSC can also fuse to existing fibres to increase peripheral myonuclear abundance in response to hypertrophic stimuli through accretion or in response to mechanical load during exercise.

Since exercise itself can act as a hypertrophic stimulus, the distinction between these two examples is unclear. Please provide additional explanation or rephrase this sentence.

5. Lines 127 and 130; The authors state that ‘no signs of necrosis were detected. However, they subsequently describe the central myonuclei as ‘a hallmark of ongoing or recent regeneration’. Necrosis occurs, followed by regeneration, so this description may cause confusion for readers. It would improve clarity if the authors provided an example demonstrating generation of central myonuclei in the absence of regeneration.

6. Lines 190-192; The authors state that ‘Their presence (Meg3+ or Ankrd1+ myonuclei) may reflect the incorporation of activated MuSC nuclei into existing myofibres following a process of regeneration and/or myonuclear accretion.’

As noted above, an alternative explanation is that pre-existing myonuclei re-express these proteins. Pax7-DTA experiments would be useful to clarify this point.

7. Lines 205-206; ‘Bulk and snRNA-seq analyses indicated that Mbnl KD promotes MuSCs activation in adult mouse TA muscles.’

Please specify which dataset(s) directly demonstrate MuSC activation in Mbnl KD muscles.

8. Line 331; Fig 5D should be corrected to Fig 5E.

9. Lines 365-368: The authors should provide appropriate references concerning myogenesis and Klf5.

10. Lines 489-491; Considering the additional experiment proposed above, the authors should discuss whether these myonuclei derive from pre-existing myonuclei or not.

Reviewer #2

(Remarks to the Author)
This paper by Todorow et al is a well-conceived, multi-layered study that links MBNL depletion to defective myonuclear maturation. The mouse AAV knockdown model is characterized functionally and histologically, MuSC involvement is probed with Pax7–DTA, and single-nucleus RNA-seq defines a transcriptional state (“Cluster X”) that is traced to human DM1 and supported by additional readouts (IF, fibre typing, and re-analysis of bulk RNA-seq). The in vivo approach and the focus on myonuclear states are timely and potentially impactful for Nat Comm. Although the comments may appear numerous, most can be addressed with clarifications, additional analyses of the current data, or improved presentation. Only a small subset may require new experiments. We are confident that these revisions are feasible and will substantially strengthen the manuscript.

Major weaknesses that need to be addressed

1. Given the relevance of Mbnl protein expression levels in the mouse model, it would be important to determine the extent of silencing at the protein level. Despite a robust 80% Mbnl1 silencing at the mRNA level, compensatory mechanisms could reduce the silencing at the protein level. By adding this new data, the experimental system would be better described.
2. At line 126, the authors state that a shift toward oxidative fiber types is detected. However, although Figure S1 shows immunodetection of different myosin isoforms, no quantification is provided. Such an analysis would serve as an important validation of the findings later presented in Figure 2A.

3. Given that control and Mbnl-KD muscles were obtained from contralateral legs of the same animal, all comparisons are inherently paired. For consistency, it would be preferable to present the data as paired in the figures (e.g., connecting lines or matched symbols per animal). This would be particularly useful to support some analyses, like Fig. 3G, where significance seems to rely on one datapoint, in addition to applying paired statistical tests. This would better reflect the experimental design and highlight within-animal differences, while reducing the impression of inter-individual variability. Note that this includes the bioinformatics analyses of bulk RNA -sequencing on Mbnl KD and control TA muscles, in which it is unclear whether this has been taken into account.

4. While the area under the relaxation curve (AUC) is a reasonable measure of myotonia, it is influenced by the magnitude of the tetanic contraction. In Fig. 1C, the authors report specific force, which varies between groups, and this variation could confound the interpretation of AUC values. It would be helpful to clarify whether higher AUCs truly reflect slower relaxation kinetics or are partly a consequence of reduced force generation. Normalizing AUC to tetanic force, or providing kinetic parameters such as $t_{1/2}$ or τ of relaxation, would strengthen the functional analysis.

5. It would be valuable to assess whether the degree of Mbnl1/Mbnl2 silencing correlates with myotonia or loss of specific force (Fig. 1B–C). Even with limited numbers, plotting knockdown levels against functional measures and reporting simple correlation coefficients (non-parametric correlation coefficients) could clarify whether the variability observed reflects differences in silencing efficiency or stems from other uncontrolled factors.

6. In Fig. 1E, the distribution of fibre cross-sectional areas is shown, and the text refers to increased heterogeneity and hypertrophy in Mbnl-KD muscles. However, no statistical analysis is provided to confirm whether the two distributions differ significantly. I recommend including an appropriate test to support the claim of altered fibre size distribution. Similar comment with Fig. 4B,C, E,F.

7. For Supplementary Fig. S2 and other comparisons, can the authors use a quantitative similarity metric to substantiate the developmental mapping beyond the UMAP visualization?

8. The experiments described in panels 3E and 3H should be explained more effectively to enhance the readers' understanding. It is not entirely clear what is being represented in the corresponding schematics, as they are not genetic crosses nor molecular depictions of what is happening molecularly.

9. Fig. 4 appears to be presented descriptively (no statistical tests indicated in the legend). Given n=3 per group and the muscle mismatch with DM1-2, this is reasonable. However, if claims like correlations are retained in the text (e.g., "increase/correlation with repeat length"), please either tone them down as trends or provide patient-level statistics and state this explicitly in the legend.

10. Could the authors detect MBNL1 in central nuclei from the patient samples to establish parallelisms with the in vivo results? Indeed, the snRNA-seq already includes this information at the nuclear RNA level.

11. In panel 4H it is unclear whether the data correspond to Ki67-positive nuclei, as indicated in the text, or to PAX7-positive nuclei, as shown in the figure. Globally, in this figure, it is not specified what each individual data point in the bar graphs represents. Also, the reported density of ~100 PAX7-positive nuclei per mm^2 appears too high if images are representative. Could the authors check whether these data are correct?

12. Because DM1 samples show both a shift toward oxidative fibres and larger fibre diameters, increases in peripheral/central myonuclei could partly reflect fibre-type composition and geometry. Please clarify whether nuclear counts were normalised for fibre size (e.g., perimeter- or area-normalised "nuclear density") and, if possible, stratify or adjust counts by fibre type (see Suppl. fibre-typing) to decouple composition/size from disease effects.

13. Fig. 5A–C (cluster abundance): In the per-sample UMAPs, cluster 4 is non-negligible in Ctrl_3 (closer to DM1_1 than expected), and cluster 2 appears larger in at least one DM1 sample than in Ctrl_3, whereas the text describes cluster-2 reduction as the clearest DM1 change. As conclusions currently rely mainly on these visualizations, please provide quantitative support at the donor level for clusters 2 and 4 and clarify the denominator used (all nuclei vs. myonuclei). Otherwise, these statements should be framed as qualitative trends given the small n and the muscle mismatch in DM1_2. Adding the quantitative support would substantially strengthen this and other claims similarly based on UMAP representations. The same is applicable to Fig. S5.

14. This text is apparently not fully accurate according to the graph shown in Fig. 5D and supplementary figure S6: "Fast-twitch nuclei (types IIa and IIx) showed increased DMPK only in patients 2 and 3, coinciding with fibre-type-specific abnormalities, including fibre size variability. Outside of slow fibres, DMPK was notably enriched only in Cluster X, but not in canonical MuSCs". In any case, a statistical test is necessary to support these conclusions. Also, define the plotting conventions (e. g. colour scale and line/dot thickness), and clarify whether values are averaged per donor or pooled, because this representation is not at all easy to follow. According to Figure S6, CNT3 displays a similar level of DMPK expression as in the patient samples, which does not align with the authors' statement at line 325. In addition, in cluster X of Figure 5D, no expression is observed in the controls, whereas in the supplementary figure, one control shows a level of expression comparable to DM1. The authors should clarify precisely what is being represented in these panels.

15. Fig. 5E (bulk validation of "Cluster X" signature): The panel works well as an external validation with an apparent severity gradient. Please (i) resolve the control-sample discrepancy (legend says 9 controls, Methods 11), and (ii) given that bulk signals can reflect tissue composition, it would be helpful to note how this was considered or include this possibility as a limitation of the approach.

16. Fig. 5F and Supplementary Fig. S7 qualitatively support increased MYOG/MYF6 and broad ANKRD1 upregulation in DM1 myonuclei. To substantiate these statements, please provide quantitative metrics focused on the biological unit (the donor).

17. Supplementary Fig. S8 shows bulk expression of ANKRD1/CDKN1A/MYF6 stratified by dorsiflexion strength (9 controls, 43 DM1), but no donor-level statistics or correlation coefficients are provided. If the text states that ANKRD1 and MYF6 are significantly upregulated and correlate with weakness, please include the corresponding donor-level tests and effect sizes, or rephrase as a descriptive trend.

18. Please state the unit of aggregation for the violins (pooled nuclei vs. per-donor averages within each cluster). Define the expression metric/scaling used and any threshold for calling a nucleus "expressing" a given gene.

19. The manuscript reports increased $Ki67^{+}$ nuclei (peripheral and central) and elevated p21/CDKN1A in central myonuclei.

To avoid misinterpretation, please clarify whether Ki67 and p21 are mutually exclusive at the single-nucleus level (cycling vs. post-fusion/arrest). If possible, include a double-immunostaining or a co-localization/overlap quantification; otherwise, state explicitly that these markers were assessed separately and reflect different stages (MuSC proliferation vs. post-fusion myonuclear arrest).
20. The link between the murine Meg3⁺/Ankrd1⁺ myonuclear states (Mbnl-KD) and the human "Cluster X" is presented largely on qualitative grounds (visual overlap and a few shared markers). It remains unclear whether Cluster X is a direct counterpart of the mouse states or a related but distinct transitional program. Because several translational inferences hinge on this connection, please make explicit your hypothesis (counterpart vs. analogous state), summarize the evidence that supports it, and delineate its limits. An additional effort to integrate results from both experimental systems would improve the quality of the paper.
21. In the Methods section, the authors state that data were first tested for normality, and if the distribution was not normal, Welch's test or the Mann–Whitney test was applied when comparing two groups. However, in the figure legends, all comparisons are indicated as being performed with t-tests or ANOVA. Could the authors please confirm whether, in all cases, the data from all groups passed the normality test?
22. If the aim is to establish a parallel between the findings in mice and the patient biopsies, could the authors clarify why they used tibialis anterior muscle for the mouse experiments? The tibialis is relatively enriched in type I (oxidative) fibers, whereas most patient biopsies are taken from biceps brachii, which is predominantly composed of type II (fast, glycolytic) fibers. Wouldn't the extensor digitorum longus (EDL), which is type II–rich, represent a more appropriate counterpart for comparison? Please justify/discuss this choice.

Minor points

1. Please use knockdown in the abstract as it is more accurate, given that 20% and 50% of Mbnl1 and Mbnl2, respectively, of gene function remains after the experimental manipulation. Also, use the official name in the keywords and introduction (muscleblind-like splicing regulator, the same in the introduction).
2. The introduction needs to incorporate new ideas regarding the DM1 pathogenesis pathway. Papers like PMID: 31116797 and PMID: 39383229 already support active repression of MBNL1 expression, in addition to the classic CUG RNA sequestration, as a cause of MBNL1 depletion.
3. Please discuss the mechanistic basis for the apparent increase of DMPK in myonuclei with an immature/transitional profile ("Cluster X").
4. Please add a reference supporting the muscle specificity of the Spc5.12 promoter. At line 116 the authors state that AAV9 was used, whereas in the Materials and Methods section (line 545) they refer to AAV9/2. This discrepancy should be clarified.
5. In Fig. 1G, some control fibres are reported as containing only a single nucleus. Since adult myofibres are multinucleated, I assume this reflects nuclei intersected in a single transverse section rather than the total nuclear content of the fibre. It would be helpful to clarify this point in the legend or methods to avoid misinterpretation.
6. In Fig. 1H, the heatmap of splicing events appears to be based on three biological replicates per group (three columns shown), whereas the legend states that at least five mice were used in each group. It would be helpful to clarify the exact sample size for the RNA-seq analysis, to avoid confusion with the numbers reported for histological and functional experiments.
7. Please provide the parameters used for clustering and UMAP projection, as these choices can markedly influence cluster separation.
8. For clarity, it would be useful to add a short statement in the figure legend or Methods indicating that the UMAP plots represent a 2D projection of nuclei based on transcriptomic similarity. This would help non-specialist readers interpret the clustering.
9. In Fig. 2B, the subclustering is described as including "all the myonuclei," but it is not explicitly stated which clusters from Fig. 2A were considered myonuclei. For clarity, please indicate whether this refers to the fibre type clusters (e.g., type I, IIa, IIx) and confirm that other mononucleated populations such as MuSCs or FAPs were excluded.
10. Please define DTA at first mention, noting that Cre activation drives cell-autonomous ablation of Pax7⁺ cells. This will aid readers unfamiliar with the abbreviation.
11. The Supplementary Fig 3 is appropriate to document MuSC ablation, but the legend mentions Mbnl1/2 qPCR while the y-axis reads "Relative Pax7 mRNA"; please correct the target gene in the legend.
12. In Fig. 3B, the y-axis and legend indicate "percentage of myonuclei positive for EdU," yet panels C and D also quantify EdU⁺ myonuclei (central vs. peripheral) and show values of zero in controls. This seems inconsistent, since the total in B should equal the sum of C and D. Please clarify precisely what population is quantified in panel B versus panels C–D (e.g., satellite cells vs. myonuclei, or different normalization), to avoid confusion.
13. In Fig. 3F and others (e.g. Fig. 4J), the representative images are presented at a very small scale, making it difficult to appreciate EdU signal or nuclear localization. Enlarging the panels or providing zoomed-in views would improve clarity.
14. Please discuss this sentence "In patient DM1_3, the highest number of central nuclei was detected, with almost all fibres presenting with central nuclei (Fig. 4C/D)" because there is no correlation with a functional parameter like ambulatory capacity, which is preserved in this patient.
15. Correct "bule" for "blue" in Supplementary Fig S5.
16. Please state whether mutant DMPK or both alleles are quantified in Fig. 5.
17. Whereas DMPK levels are shown in the order "CTR-DM1" in Fig. 5D they are shown reversed in Suppl S6. Please show them in the same order to improve readability.
18. Line 344 should refer to Fig. 5F, instead of E.
19. In Fig. 6A, each cluster×gene tile appears split into multiple vertical bars. Please specify what each sub-bar represents, the ordering (Ctrl_1–3, DM1_1–3), and the expression metric/scaling used. This will help readers interpret donor-level consistency rather than pooled values.

20. No data in the paper seems to support this sentence "we also propose additional mechanisms such as impaired nuclear migration due to altered nucleo-cytoskeletal integrity", which should be rewritten as state of the art, not a new proposal arising from the paper.
21. In light of previous comments, note that the statement in line 482 of the paper must be toned down unless it is statistically backed.
22. Please include the actual sequences targeted by shRNAs used in the AAV (lines 525-529), for better reproducibility of results reported.
23. Please confirm whether myotonia was measured under constant temperature conditions, as this may impact measurements.
24. The Methods state that nuclei isolation was performed "as previously described, with some modifications optimized for the muscle," but only the addition of BSA is explicitly mentioned. For reproducibility, it would be important to clearly specify all modifications introduced relative to the published protocol.
25. Please clarify in the Methods how fiber area quantification was performed (e.g., software, number of fibers analyzed, blinding).
26. The sequences of the primers used are not provided. For reproducibility and transparency, please include the exact primer sequences in the corresponding section.

Reviewer #3

(Remarks to the Author)
Todorow et al. show the muscle stem cells (MuSCs) are activated and fuse to myofibers when Mbnl 1/2 are reduced in myofibers, a common feature of myotonic dystrophy. By using mouse genetic models and snRNA sequencing they showed that MuSCs contribute new nuclei into Mbnl 1/2 KD myofibers and they make interpretations that these nuclei do not immature transcriptional state. By using snRNA sequencing and histology from human muscle biopsies of healthy and MD patients, Todorow et al. showed that MuSCs are also activated and found the presence of an immature myonuclear cluster, similar to what they observed in the Mbnl 1/2 KD muscles. The snRNAseq data provide a good resource for the muscle community. There are not major concerns about the technical validity of the data. However, I am not able to support publication because the interpretations are invalid and not supported by the data, there is a major disconnect between the themes in the manuscript (MuSCs, snRNAseq in mouse, snRNAseq in human), and the overall premise of the paper is not communicated well.
1. The main premise of the paper as stated in the abstract is that there are questions whether DM1 maintains its regenerative potential or shows MuSC defects. Based on my review of the literature, it already known that there is regeneration and MuSC defects. It seems obvious that MuSCs contribute to the central nuclei in DM1 muscle. The authors should communicate more clearly about the specific questions in DM1 related to MuSCs and how their study contributes.

2. Line 29-30: what are the data that central nucleation is independent of degeneration? Some negative effects must be occurring in myofibers since the manipulation is myofiber-specific. The authors have not assessed what is happening in myofibers.

3. Line 34-36: This is a major over-interpretation and based on the literature is likely to be incorrect. I don't see any data indicating that the cells fusing into DM1 myofibers give rise to 'immature' nuclei. It is just as likely that these are nuclei already in the myofiber that exhibit this signature. Assessing gene expression in the DM1 model after MuSC ablation could be one way to test this idea.

4. The statement that this immaturity is 'likely' due to RNA effects on transcriptional reprogramming is also not substantiated. It can be appropriate to include these type of comments in the discussion but not in the abstract.

5. Line 36-37: I don't think MuSCs have been previously overlooked in DM1 pathology. In fact there was a Nature Communications paper (Conte et al 2023) that showed senolytics can restore myogenesis in DM1. Also, what is the 'role' of MuSCs that this current work has defined?

6. The model used to knockdown Mbnl1/2 in myofibers is perplexing because DM1 is also caused by Mbnl1/2 defects in MuSCs. Based on comments throughout the paper (such as Lines 211-212), are the authors thinking that the Mbnl depletion is occurring in MuSCs? This can be directly tested since AAVs can transduce MuSCs albeit at lower levels than myofibers. I don't see any direct data to support this and based on their system I was thinking that depletion occurs mostly in myofibers, causing defects in myofibers leading to MuSC activation and fusion.

7. Lines 104-106: It is unclear whether the data suggest impaired myonuclear maturation because the signature they are detecting could be from recently fused cells.

8. Lines 124-126: what are the data showing fibre splitting?

9. Lines 164-165: when was snRNAseq performed after Mbnl1 KD? Should be added to results or figure legend.

10. Lines 165-167: Fig 2A should highlight the distinct myonuclear population.

11. Lines 180-182: It is good practice to test transcriptional states with other available datasets. However, wouldn't it be more appropriate to do this with a dataset from adult regeneration (mdx, denervation, or muscle overload) instead of postnatal development?

12. Figure 2: Full marker gene lists are needed to evaluate the emerging myonuclear populations in DM1. These are not provided. How similar are the Meg3+ and Ankrd1+ myonuclei during postnatal development and DM1? Are they simply reversion to immaturity that is normal or immaturity that is maladaptive? Perhaps integration of the datasets would be helpful or a venn diagram of marker gene lists.

a. I am also not sure that Ankrd1 and Meg3 signifies 'immaturity'. It is difficult without seeing all genes enriched in those populations. Also, multiple papers in the field show Ankrd1 is associated with times of muscle growth and remodeling.

13. For snRNAseq in figure 2, how many samples were analyzed? There should be some validation of the data in independent animals. The authors could do RNAscope for marker genes. This would also help determine if these populations are centrally or peripherally located, which could indicate whether a signature is derived from MuSCs.

14. There is a disconnect between the snRNAseq analysis and the MuSC accretion phenotype in the Mbnl 1/2 KD muscles. The snRNAseq is focused on characterizing the transcriptional changes in the immature myonuclear population (which the authors incorrectly say is MuSC-derived) but nothing on the stem cell compartment. Also, are activated myoblasts found in this dataset, which would be expected.

a. There is also a lack of connection between the human and mouse transcriptomic data, especially within the immature myonuclear populations and stem cells. A better characterization will make the story more compelling. It is not intuitive to the reader that what is observed in the mouse is also present in human samples. For instance, authors characterized p21 in human samples but p21 is not even mentioned in the mouse model, which makes the rationale weak or not clear. There are many examples of these discrepancies.

Minor comments:

1) Line 308-309 authors argue that IIx fibers are reduced in human biopsies, but data is highly variable, so they should either increase the n or remove the statement.

2) Graph Figure 4E is hard to distinguish the categories. A color bar graph might be better.

Reviewer #4

(Remarks to the Author)

I co-reviewed this manuscript with one of the reviewers who provided the listed reports. This is part of the Nature Communications initiative to facilitate training in peer review and to provide appropriate recognition for Early Career Researchers who co-review manuscripts.

Version 1:

Reviewer comments:

Reviewer #1

(Remarks to the Author)

I appreciate that the authors sincerely addressed most of my concerns. The new data indicate a limited contribution of MuSCs to the increased total number of myonuclei (response to original comment #1). In addition, the increased number of myonuclei derived from muscle satellite cells does not appear to contribute to the pathology or functional deficits observed in DM1 model mice. The authors speculate about the contribution of non-myogenic cells; however, these cells have not been identified. In addition, the authors did not address my original comment #6; therefore, it remains unclear why MuSCs begin to proliferate in DM1.

Lines 203-: myonuclear transcriptional profiles revealed decreased expression of Mbnl1

--- Fig. 2A shows the expression of MBNL1/2 in KD mice. To what extent is MBNL1/2 expression decreased? If the majority of myonuclei express MBNL1/2, this would suggest that many myofibers have died and were subsequently replaced by satellite cell–derived regenerated myofibers.

In contrast, the results in Fig. S5C indicate that even after 10 weeks, the expression levels of MBNL1/2 remain very low. It is therefore important to clearly indicate how many myonuclei with reduced MBNL1/2 levels (i.e., pre-existing myonuclei) remain, as this would support the notion that myofiber necrosis is not overt.

Lines 234-: Their presence may reflect the incorporation of activated MuSC nuclei into existing fibres following a process of regeneration and/or myonuclear accretion.

---'regeneration' should be replaced with 'postnatal development'.

Lines 237-

---Why did the authors use a denervation model? Do MuSCs become activated and enter a proliferative state in denervated

muscles? The authors should clearly describe the aim of this experiment. If the possibility of neurodegeneration occurring in MBNL-KD is being considered, this should be clearly stated. In fact, MyoD and Myog expression is increased in myofibers following denervation (Cell. 2010 Oct 1;143(1):35-45. doi:10.1016/j.cell.2010.09.004.).

Lines 241; Collectively, this suggests that these populations are in response of muscle to physiological perturbations such as injury or overload (Supplementary Fig. S4C,D).
---Related to the above point, did the authors use denervation as an injury model?

SFig 5H (related to original comment #3 and #4):
---There are a significant number of EdU-positive myonuclei despite the absence of MuSCs. Is it possible that pre-existing myonuclei incorporate EdU? While the authors mention potential non-myogenic contributions, the possible contribution of pre-existing myonuclei is not discussed.
The authors state that MBNL1 is expressed in Meg3⁺ or Ankrd1⁺ myonuclei. Is this result described in the main text? Are there no MBNL1-negative myonuclei ing the Meg3⁺ or Ankrd1⁺ myonuclei? Similarly, was MBNL expression detected in the EdU-positive myonuclei observed in Pax7-DTA mice? In addition, is it possible that pre-existing myonuclei underwent division, leading to a reversion of MBNL1/2 expression to baseline levels?

Reviewer #2

(Remarks to the Author)
I would like to thank the authors for the thorough and careful work carried out during this revision. They have addressed my previous comments with clarity and added substantial new analyses and explanations where needed. The manuscript has improved considerably in both rigor and presentation. I appreciate the effort invested in refining the study and in providing detailed, constructive responses throughout.

Reviewer #3

(Remarks to the Author)
The authors were responsive to critiques and the manuscript is improved. There are altered interpretations and the main piece of new data is the snRNAseq data on Pax7-DTA; Mbnl KD muscle, which clarifies the claim that fusion of MuSCs is only partially impacting gene expression in myofibers. While I am supportive of publication, I would strongly encourage the authors to highlight, perhaps in abstract, final paragraph of intro, and/or discussion, that there is not an obvious effect on the muscle phenotype (myotonia, muscle function) when MuSCs are deleted. I think this is an important result when much of the discussion is about these transient nuclear states of unknown significance, and mentioning no effect on the muscle phenotype does not detract from the main point of the paper that Mbnl loss in myofibers induces fusion.

I also have one minor concern about the below excerpt in the discussion:

546 Our findings therefore provide, to our knowledge, the first direct in vivo evidence in a
547 mammalian disease model, and supportive evidence in human DM1 muscle, that MuSC
548 fusion occurs under pathological conditions in muscle.

This sentence reads that this paper is the first to show MuSC fusion under pathological conditions. That is certainly not true as MuSC fusion is known to occur in other muscle pathologies such as DMD.

Reviewer #4

(Remarks to the Author)
I co-reviewed this manuscript with one of the reviewers who provided the listed reports. This is part of the Nature Communications initiative to facilitate training in peer review and to provide appropriate recognition for Early Career Researchers who co-review manuscripts.

Version 2:

Reviewer comments:

Reviewer #1

(Remarks to the Author)
As the authors note, MuSC activation is likely governed by multiple pathways rather than a single mechanism; thus, a request for detailed elucidation of the underlying mechanism would be beyond the reasonable scope of the present study. In addition, the authors have addressed all other concerns in a careful and constructive manner. Therefore, I have no further requests for revision.

Reviewer #3

(Remarks to the Author)
I have no further concerns.

Open Access This Peer Review File is licensed under a Creative Commons Attribution 4.0 International License, which permits use, sharing, adaptation, distribution and reproduction in any medium or format, as long as you give appropriate credit to the original author(s) and the source, provide a link to the Creative Commons license, and indicate if changes were made.
In cases where reviewers are anonymous, credit should be given to 'Anonymous Referee' and the source.
The images or other third party material in this Peer Review File are included in the article's Creative Commons license, unless indicated otherwise in a credit line to the material. If material is not included in the article's Creative Commons license and your intended use is not permitted by statutory regulation or exceeds the permitted use, you will need to obtain permission directly from the copyright holder.
To view a copy of this license, visit https://creativecommons.org/licenses/by/4.0/

REVIEWER COMMENTS

Reviewer #1 (Remarks to the Author):

In this study, the authors integrated Mbnl knockdown mouse models, including MuSC-depleted mice, together with human samples to elucidate the mechanisms underlying the increase in total and central myonuclei, a hallmark of DM1 pathology. Moreover, they identified a CUG-repeat–dependent Cluster X in DM1 which is characterized by an immature transcriptional profile, elevated DMPK expression, and increased p21 expression. Overall, this work is highly interesting; however, the conclusions drawn from the Pax7-DTA model remain limited, and more detailed analyses are required to clarify the contribution of MuSCs to the pathophysiological features of DM1. A critical issue is whether MuSC activation, proliferation, and fusion occur independently of muscle degeneration and regeneration.

Major points

1. In the abstract and discussion, the authors state that ‘central myonuclei accumulation independent of degeneration’. However, even if overt signs of degeneration are absent in the analyzed samples, degeneration may have occurred at an earlier stage. In Figure 1, it is essential to clarify whether central nuclei arise primarily from MuSC–myofiber fusion, from regeneration following degeneration, or through both mechanisms.

Compared to other muscular dystrophies characterized by active cycles of regeneration and degeneration, the absence of marked variation in myofiber size (Fig. 1E) supports the notion of limited regenerative and degenerative activity in this model. Analyzing the relationship between myonuclear number and myofiber size would further strengthen the authors’ interpretation. If the authors intend to make this claim, it is essential to clearly explain that the increased frequency of centrally located nuclei is primarily attributable to MuSC-mediated fusion with pre-existing myofibers, supported by evidence including human studies. Additionally, the results of myofiber number should be included in Figure 1.

> We agree that, even in the absence of necrosis, prior degenerative events cannot be formally excluded and that additional analyses are required to clarify the origin of centrally located nuclei.
>
> To strengthen our interpretation, we have now analysed the relationship between myofibre cross-sectional area (CSA) and myonuclear number, distinguishing between centrally located and peripheral nuclei. These new analyses provide insight into whether central nuclei are more consistent with degeneration–regeneration cycles or with MuSC-mediated fusion into pre-existing myofibres.
>
> We found that fibres containing central nuclei span a broad CSA range (≈250–5000 μm²), with a peak in intermediate-sized fibres (≈1000–2000 μm²), whereas peripheral myonuclei were preferentially enriched in larger fibres (>4000 μm²). Importantly, fibres containing one or two central nuclei were significantly smaller than the overall myofibre population, while fibres harbouring three or more central nuclei exhibited sizes comparable to the mean CSA of all fibres. In addition, the total number of myofibres was not significantly altered in Mbnl-knockdown muscles.

Importantly, this distribution is inconsistent with canonical degeneration–regeneration cycles, which typically produce clusters of uniformly small, newly regenerated fibres with centralized nuclei. Instead, centrally nucleated fibres in our model span a wide size range and are not accompanied by fibre loss, supporting a mechanism of myonuclear accretion within pre-existing myofibres.

These results are now included in Supplementary Figure S2A-C and described in the Results section as follow (line 149):

"However, the total number of myofibres was not significantly altered in Mbnl-depleted TA muscles (Supplementary Fig. S1H). … , associated with a 2-fold increase of peripheral nuclei per fibre (Fig. 1F-H). Analysis of the relationship between myofibre size and myonuclear number (both centralized and peripheral) further revealed that fibres containing centralized nuclei had cross-sectional areas ranging from 250 to 5000 μm^2, whereas peripheral myonuclei were more frequent in larger fibres (above 4000 μm^2; Supplementary Fig. 2A-B). Moreover, fibres containing one or two centralized nuclei were significantly smaller than the average of all myofibres, while those with three or more centralized nuclei exhibited sizes comparable to the mean of total myofibres (Supplementary Fig. S2C)."

2. In this study, the authors used the Spc5.12 promoter instead of Mhck7. The direct effect of AAV-mediated silencing on MuSCs needs to be clarified, even though the expression level of Mbnl1 may be very low. Alternatively, based on past reports, etc., please state that there is no direct effect of Mbnl silencing by AAV on MuSC.

We agree that any potential direct effect of AAV-mediated Mbnl silencing on muscle stem cells (MuSCs) must be carefully addressed. Several lines of evidence indicate that such direct effects are unlikely under our experimental conditions.

First, the spC5.12 promoter used in this study consists of a combination of four muscle-specific TFBSs (TEF1, SRE, MEF1, and MEF2) and a core promoter derived from the chicken skeletal muscle α-actin gene. These transcription factors are predominantly expressed or activated during myogenic differentiation. As a result, Spc5-12 is largely inactive in proliferating myoblasts and non-muscle cells but becomes transcriptionally active in post-mitotic myotubes and mature myofibre nuclei. This differentiation-dependent and muscle-specific regulation is an intrinsic feature of the promoter's design, providing a mechanistic explanation for its activity profile observed *in vivo* and *in vitro* (Li et al. 1999; Wang et al. 2008; Rincon et al. 2015; Skopenkova et al. 2021). It is therefore not active in quiescent MuSCs, and expression from this promoter in resting MuSCs is not expected.

Second, multiple studies have shown that transduction of MuSCs by AAV serotype 2/9 is very inefficient , typically below 1–2%, even at high intramuscular or systemic doses (Boutin et al. 2010; Arnett et al. 2014), making substantial direct delivery of the silencing cassette to MuSCs improbable.

Third, our single-nucleus RNA-seq data support a muscle fibre–restricted effect. The decrease in Mbnl1 transcript was observed in myonuclei TypeIIx/a and Type IIb fibres

while no downregulation was observed in MuSC cells (Supplementary Fig. S3A and Supplementary Tables T3-5).

Taken together, these points argue against a meaningful direct effect of AAV-mediated Mbnl silencing on MuSCs in our study. We have added the above cited references to the revised manuscript and explicitly mention the snRNA-seq observation in the Result section.

3. It remains unclear whether the reactivation of Meg3 and Ankrd1 is restricted to MuSC-derived nuclei or also occurs in pre-existing myonuclei. This issue could be addressed by analyzing the Pax7-DTA model.

To address this point, we have now performed snRNA-seq on muscles from Pax7-depleted mice 10 weeks after Mbnl KD (Pax7-DTA Mbnl KD) and compared them to Mbnl KD in $Pax7^{CreERT2}$ control mice (Pax7-CreERT2 Mbnl KD). The newly generated data are now included in Figure 4F–H.

Using this approach, we first confirmed that DTA efficiently reduces the $Pax7^{+}$ cell population by approximately 85%, validating effective MuSC depletion. Notably, Pax7 depletion resulted in a partial reduction of the *Meg3*$^{+}$/*Ankrd1*$^{+}$ nuclear subpopulation (Figure 4G-H; Supplementary Fig. S6B). This suggests that although Pax7+ cells contribute, they are not the only source of these new populations.

Importantly, this experiment does not allow us to definitively determine whether *Meg3* and *Ankrd1* reactivation occurs within pre-existing myonuclei or arises from fusion of non–Pax7-derived progenitor cells. However, in the original experiment, we observed that both *Meg3*$^{+}$ and *Ankrd1*$^{+}$ populations express *MBNL1*, suggesting that they indeed derive from a cell type that was not targeted by the AAV virus for knockdown.

Taken together, these results support a model in which *Meg3* and *Ankrd1* reactivation reflects a mixed origin, involving both MuSC-derived nuclei and additional myonuclear populations, in agreement with both the mouse genetic model and human disease data.

4. Figure 3: The Pax7-DTA results clearly demonstrate that majority of central nuclei in DM1 originate from MuSCs. Were the other central myonuclei in Pax7-DTA mice originated from pre-existing myonuclei or from remained MuSCs? Additionally, the authors need to analyze the total number of myonuclei and skeletal muscle mass in Pax7-DTA model as shown in Figure 1A and 1G. If a significant increase in peripheral myonuclei is observed in Pax7-DTA, this would strongly suggest division of pre-existing myonuclei. By investigating the number of myofibers and muscle mass, it will become clear whether these are due to myofiber splitting or to new myofiber formed via MuSC.

We thank the reviewer for this insightful comment and for suggesting additional analyses to clarify the origin of central and peripheral myonuclei in the $Pax7^{DTA}$ model.

Our data indicate that, in $Pax7^{DTA}$ mice, ablation of MuSCs led to a decrease in central nuclei in Mbnl KD TA muscles, while the number of peripheral myonuclei and overall muscle mass were unaffected, as well as myotonia and specific muscle force. In addition, in Mbnl KD TA muscles, we observed no change in total myofibre number,

neither in Pax7^{DTA} mice further supporting that peripheral myonuclei and fibre number remain stable even when central nuclei are reduced.

Taken together, these observations indicate that the majority of central nuclei originate from Pax7^{+} MuSCs. However, the unaffected number of peripheral nuclei in Pax7^{DTA} mice raises the possibility that they may derive from non-Pax7 muscle stem/progenitor populations or other unidentified sources. This question would need further investigations to fully delineate the origin of peripheral nuclei.

These data are now presented in Figure 4A-C and Supp. Fig. S5D-F, with the associated text in the Results section.

5. Figure 4J: The detection of Ki67+ myonuclei is intriguing. Are Ki67+ central and peripheral myonuclei observed in the mouse model as observed in human samples? If so, the authors can address whether these nuclei originated from MuSCs or not using the Pax7-DTA model. The authors need to clarify the source of Ki67+ myonuclei and also examine the contribution of pre-existing myonuclei. EdU-injection experiment also should be performed whether pre-existing myonuclei uptake EdU by the loss of Mbn1.

To investigate the proliferative status of myonuclei, we performed Ki67 staining in TA muscles from wild-type mice following Mbnl KD. We observed fewer than 0.5% of Ki67^{+} centralized nuclei, while a small proportion of peripheral myonuclei were Ki67^{+} (~2%), although this difference was not statistically significant.

This difference is likely explained by the distinct temporal resolution of Ki67 detection. Ki67 marks actively cycling cells within a narrow time window, which may vary substantially between human and mouse tissue. In particular, gradual or transient proliferative activity may be missed by Ki67 staining.

In contrast, EdU incorporation provides a cumulative readout of S-phase entry, permanently labeling nuclei even after proliferative activity has ceased. To directly assess the contribution of proliferating Pax7^{+} MuSCs, we therefore performed EdU incorporation experiments on Pax7-DTA mice. Importantly, EdU labelling revealed a significant decrease in both EdU^{+} centralized nuclei and EdU^{+} peripheral myonuclei in Pax^{DTA} Mbnl KD muscles compared to Mbnl KD muscles in Pax7^{CreERT2} control mice. These findings indicate that newly incorporated nuclei, particularly centralized nuclei, are largely derived from Pax7^{+} MuSCs, and that a subset of newly added peripheral myonuclei also originates from Pax7^{+} MuSCs.

Taken together, these observations support that the majority of central nuclei arise from MuSCs and further suggest that MuSCs partially contribute to peripheral myonuclear accretion following Mbnl knockdown. The persistence of total peripheral myonuclear number despite reduced EdU incorporation may likely reflects the long-term stability of pre-existing myonuclei.

These data are now presented in Figure 4A,D-E and described in the Results section.

6. Figure 4: In relation to Comment 1, it is essential to confirm that this MuSC proliferation is not simply a consequence of degeneration, but rather results from the disruption of quiescence signaling in MuSCs, caused directly or indirectly by MBNL deficiency in myofibers. Based on the snRNA-seq data, the authors should discuss potential candidate factors that may impair MuSC quiescence or promote their activation.

We fully agree that it is critical to distinguish MuSC activation driven by degeneration-associated regeneration from activation resulting from altered quiescence signaling, potentially secondary to MBNL deficiency in myofibres. These findings argue against a model in which MuSC activation is driven by a strong cell-autonomous loss of quiescence signaling within MuSCs themselves.

To address this, we examined MuSC transcriptional states in our mouse snRNA-seq dataset. Only a limited number of genes (n=4) are differentially expressed (*Gm20721*, *Morrbid*, *Col8a1* and *Cdh13*), and these changes do not indicate a disruption of quiescence signaling. These findings argue against a model in which MuSC activation is driven by a strong cell-autonomous loss of quiescence signaling within MuSCs themselves.

In parallel, exploratory analysis of the human snRNA-seq dataset revealed alterations in signaling-related pathways within DM1 satellite cells, including increased *IGF1* expression and changes associated with ECM–integrin interactions (e.g., *LAMA2–ITGB1*), as well as enrichment of Wnt signaling–related gene ontology categories. Notably, LAMA2–ITGB1 signaling has been implicated in MuSC niche regulation and activation through extracellular matrix remodeling mechanisms. While these observations are not included in the manuscript, they are consistent with altered niche and signaling environments in human DM1 muscle.

Importantly, incorporation of these human-specific signaling analyses would require substantial additional validation and cross-species contextualization, which falls beyond the scope of the present study. To maintain conceptual and analytical consistency between the mouse model and human datasets, we therefore chose not to include these preliminary observations in the revised manuscript.

Taken together, our data support a model in which MuSC activation arises predominantly through indirect, microenvironmental mechanisms rather than primary degeneration-driven regeneration or strong MuSC-intrinsic defects.

Minor comments:

1. Since both muscle fiber and myofiber are used, the terminology should be standardized as myofiber throughout the manuscript.

We now used “myofibre” throughout the manuscript.

2. Line 34; The term “repair” generally implies the restoration process following deterioration or damage. If the authors intend to argue that MuSC activation and proliferation occur independently of degeneration, this reviewer believes that the term “repair” should be avoided, as it may confuse readers.

We agree that “repair” could be misinterpreted as implying prior degeneration. Our intention was to describe MuSC fusion and myonuclear accretion as a compensatory or homeostatic process rather than classic tissue repair. We have revised the text accordingly and now use terms such as “homeostatic maintenance,” “myonuclear accretion,” or “adaptive fusion” in place of “repair” to avoid confusion.

Corresponding wording changes have been implemented in the Abstract, Introduction, and Discussion where appropriate.

3. Lines 59-61; ‘Indeed, MBNL proteins bind with high affinity to YGCY motifs and, therefore, to expanded CUG repeats in proportion to the size of this expansion.’

It would aid reader comprehension to add the clarification: “Y = pyrimidine.”

We added this now for reader comprehension.

4. Lines 81-83; MuSC can also fuse to existing fibres to increase peripheral myonuclear abundance in response to hypertrophic stimuli through accretion or in response to mechanical load during exercise.

Since exercise itself can act as a hypertrophic stimulus, the distinction between these two examples is unclear. Please provide additional explanation or rephrase this sentence.#

We thank the reviewer for this helpful suggestion. The sentence referenced by the reviewer has been revised during restructuring of the Introduction. To improve conceptual clarity regarding MuSC fusion to existing fibres, the text in the discussion (Line 542 ff) now reads:

“Instead, repair of sublethal myofibre damage has been shown to occur through MuSC-independent mechanisms involving redistribution of resident myonuclei, whereas MuSC fusion to existing fibres is most clearly supported in contexts of physiological adaptation, such as exercise-induced hypertrophy and myonuclear accretion[56].”

5. Lines 127 and 130; The authors state that ‘no signs of necrosis were detected. However, they subsequently describe the central myonuclei as ‘a hallmark of ongoing or recent regeneration’. Necrosis occurs, followed by regeneration, so this description may cause confusion for readers. It would improve clarity if the authors provided an example demonstrating generation of central myonuclei in the absence of regeneration.

Our intent was to indicate that, in our Mbnl-KD model, central nuclei arise through MuSC fusion events rather than as a consequence of myofibre necrosis and regeneration. Indeed, the literature shows that centrally located nuclei can appear in the absence of acute degeneration: for example, during myonuclear accretion with mechanical overload or exercise (Masschelein et al., PMID: 32646489; Murach et al., PMID: 32881361). We have revised the manuscript to clarify this distinction and provided reference to these “non-degenerative central nucleation” contexts in the introduction and discussion, thereby improving clarity for the reader.

Revised manuscript text

Before (Lines 127–130):

"A shift toward oxidative fibre types was also observed while no signs of necrosis were detected in Mbnl-depleted TA muscles, features that were absent in contralateral control muscles (Fig. 1D, E, Supplementary Fig.1C-E). Additionally, a two-fold increase in the total number of nuclei per fibre was observed, with centrally located nuclei, a hallmark of ongoing or recent regeneration, present in 18% of myofibres (Fig. 1F, G)."

Revised (Line 148 ff) :

"However, the total number of myofibres was not significantly altered in Mbnl-knockdown TA muscles (Supplementary Fig. S1H). A shift toward oxidative fibre types was observed in Mbnl-depleted TA muscles, with type IIa fibres increasing from 15% to 62% and type IIb fibres decreasing from 50% to 25%; these features were absent in contralateral control muscles (Supplementary Fig. S1I-K). Importantly, these histological changes occurred in the absence of major myofibre degeneration or necrosis, consistent with previous reports in MBNL KO mice[26]."

6. Lines 190-192; The authors state that 'Their presence (Meg3+ or Ankrd1+ myonuclei) may reflect the incorporation of activated MuSC nuclei into existing myofibres following a process of regeneration and/or myonuclear accretion.'

As noted above, an alternative explanation is that pre-existing myonuclei re-express these proteins. Pax7-DTA experiments would be useful to clarify this point.

We thank the reviewer for this important comment and for highlighting the alternative possibility that *Meg3*$^{+}$ and *Ankrd1*$^{+}$ myonuclei could arise from transcriptional reprogramming of pre-existing myonuclei, rather than exclusively from fusion of activated MuSCs.

To address this point, we considered evidence from multiple complementary approaches. As discussed above, Pax7^{DTA} experiments revealed a partial but significant reduction of the *Meg3*$^{+}$ and *Ankrd1*$^{+}$ myonuclear populations, indicating that MuSC-derived nuclei contribute to their emergence, but are not their sole source. This finding supports a model in which these populations have a mixed origin.

In addition, we observed that *Mbnl1* transcripts were detectable in both *Meg3*$^{+}$ and *Ankrd1*$^{+}$ myonuclear clusters (Supplementary Tables T6, T7). While this signal may partially reflect residual RNA due to incomplete knockdown, and acknowledging that RNA interference primarily acts in the cytoplasm, these data also raise the possibility that these nuclei derive from a cell population that was not initially targeted by the shRNA AAV injection and therefore retained *Mbnl1* expression prior to fusion.

Taken together, these data indicate that the presence of *Meg3*$^{+}$ and *Ankrd1*$^{+}$ myonuclei cannot be attributed solely to incorporation of MuSC-derived nuclei. Rather, our results are consistent with a model in which these transcriptionally immature myonuclei arise from multiple sources, including MuSC fusion as well as potentially other myonuclear or muscle-resident progenitor populations.

7. Lines 205-206; 'Bulk and snRNA-seq analyses indicated that Mbnl KD promotes MuSCs activation in adult mouse TA muscles.'
Please specify which dataset(s) directly demonstrate MuSC activation in Mbnl KD muscles.

> We have now included dot plots (Supplementary Figure S3B) that show an increase in expression of classic myoblast activation markers such as Myog and Myod1 in the myonuclear populations. Additionally, we show GO term analysis for the genes upregulated in the myonuclei (Supplementary Figure S3C,D).

8. Line 331; Fig 5D should be corrected to Fig 5E.

> We corrected this.

9. Lines 365-368: The authors should provide appropriate references concerning myogenesis and Klf5.

> We have now added a specific citation demonstrating the established role of Klf5 in skeletal muscle differentiation and regeneration. These studies show that KLF5 cooperates with MyoD and Mef2 to promote myogenic differentiation. We have incorporated the following key reference:
>
> Hayashi, S. et al. "Klf5 regulates muscle differentiation by directly targeting muscle-specific genes in cooperation with MyoD in mice" PMID: 27743478

10. Lines 489-491; Considering the additional experiment proposed above, the authors should discuss whether these myonuclei derive from pre-existing myonuclei or not.

> We thank the reviewer for this important point. In light of the additional experiments performed, we have expanded the Discussion to explicitly address whether the observed myonuclei could derive from pre-existing myonuclei.

Reviewer #2 (Remarks to the Author):

This paper by Todorow et al is a well-conceived, multi-layered study that links MBNL depletion to defective myonuclear maturation. The mouse AAV knockdown model is characterized functionally and histologically, MuSC involvement is probed with Pax7–DTA, and single-nucleus RNA-seq defines a transcriptional state ("Cluster X") that is traced to human DM1 and supported by additional readouts (IF, fibre typing, and re-analysis of bulk RNA-seq). The in vivo approach and the focus on myonuclear states are timely and potentially impactful for Nat Comm. Although the comments may appear numerous, most can be addressed with clarifications, additional analyses of the current data, or improved presentation. Only a small subset may require new experiments. We are confident that these revisions are feasible and will substantially strengthen the manuscript.

Major weaknesses that need to be addressed

1. Given the relevance of Mbnl protein expression levels in the mouse model, it would be important to determine the extent of silencing at the protein level. Despite a robust 80% Mbnl1 silencing at the mRNA level, compensatory mechanisms could reduce the silencing at the protein level. By adding this new data, the experimental system would be better described.

We thank the reviewer for this important suggestion. To better characterize the extent of MBNL silencing at the protein level, we performed Western blot analyses of Mbnl1 and Mbnl2 in Mbnl KD muscles.

We observed an ~50% decrease in Mbnl1 protein, which is less pronounced than the ~80% reduction at the mRNA level, suggesting post-transcriptional compensation or protein stability effects. Interestingly, in contrast to the mRNA data, we detected a mean 1.5-fold increase in Mbnl2 protein, indicating a potential compensatory mechanism as already reported in the literature (Lee et al., 2013). Analysis of splicing from RNA-seq revealed abnormal inclusion of Mbnl2 exon 9, which is normally regulated by Mbnl1 protein. Inclusion of exon 9 is known to stabilize the Mbnl2+ex9 isoform (Nitschke et al., 2023) and likely contributes to the observed increase in Mbnl2 protein. Together, these data suggest that co-treatment with shmiR against Mbnl2 in our model would mitigate the compensatory increase in Mbnl2 in response to Mbnl1 knockdown. Importantly, despite this partial compensation at the protein level, Mbnl KD muscles exhibit DM1-relevant phenotypes, including myotonia, muscle weakness, fibre-type transitions, and extensive central nucleation. These observations indicate that functional MBNL activity remains sufficiently disrupted to drive disease-associated pathology.

These data are presented in Supplementary Fig. S1C-F and described in the Results section and provide a more complete description of the molecular consequences of Mbnl1 and Mbnl2 silencing in our experimental system:

"To assess whether these changes were reflected at the protein level, we performed Western blot analysis of MBNL1 and MBNL2. Consistent with the transcript data, MBNL1 protein levels were decreased by approximately 50% in Mbnl KD muscles. In contrast, MBNL2 protein abundance was increased (Supplementary Fig. S1C-E). RNA-seq analysis revealed abnormal inclusion of Mbnl2 exon 9, a splicing event regulated

by MBNL1. Inclusion of this exon is known to stabilize the MBNL2+ex9 isoform[44]", likely contributing to the elevated MBNL2 protein levels (Supplementary Fig. S1F) as previously described."

2. At line 126, the authors state that a shift toward oxidative fiber types is detected. However, although Figure S1 shows immunodetection of different myosin isoforms, no quantification is provided. Such an analysis would serve as an important validation of the findings later presented in Figure 2A.

In response to this comment, we have now quantified the myosin isoform immunostaining originally shown in Supplementary Fig. S1.

This analysis confirmed a shift in fibre type composition, with type IIa fibres increasing from 15% to 62% and type IIb fibres decreasing from 50% to 25%, validating the oxidative fibre type shift described in the manuscript and supporting the findings presented in the Results section. In addition, we have modified Supplementary Fig. S1 (Supplementary Fig. SI-K) to provide a more comprehensive view, including representative images of fibre types and the corresponding quantification, to facilitate interpretation. The results text was modified accordingly (Line 150 ff):

"A shift toward oxidative fibre types was observed in Mbnl-depleted TA muscles, with type IIa fibres increasing from 15% to 62% and type IIb fibres decreasing from 50% to 25%; these features were absent in contralateral control muscles."

3. Given that control and Mbnl-KD muscles were obtained from contralateral legs of the same animal, all comparisons are inherently paired. For consistency, it would be preferable to present the data as paired in the figures (e.g., connecting lines or matched symbols per animal). This would be particularly useful to support some analyses, like Fig. 3G, where significance seems to rely on one datapoint, in addition to applying paired statistical tests. This would better reflect the experimental design and highlight within-animal differences, while reducing the impression of inter-individual variability. Note that this includes the bioinformatics analyses of bulk RNA -sequencing on Mbnl KD and control TA muscles, in which it is unclear whether this has been taken into account.

We agree that, given that control and Mbnl KD muscles were obtained from contralateral legs of the same animal, the data are inherently paired. Accordingly, we have updated figures to display paired data points (connecting lines between contralateral muscles) in relevant panels (Fig. 1A-C; Fig. 3F (Tomato intensity), Supplementary Fig. S5E-G) and re-analysed the data using paired statistical tests (paired t-tests), which better reflect within-animal differences and reduce the impression of inter-individual variability. This update includes all relevant panels. Importantly, this re-analysis did not modify the statistical significance of the results.

Regarding the bulk RNA-seq analysis, the data were processed using the DESeq2 pipeline, which by default does not implement paired statistics. While we acknowledge that it would be possible to incorporate a paired design to further account for within-animal variability, we believe that the overall conclusions are robust and strongly supported by other experimental readouts.

4. While the area under the relaxation curve (AUC) is a reasonable measure of myotonia, it is influenced by the magnitude of the tetanic contraction. In Fig. 1C, the authors report specific force, which varies between groups, and this variation could confound the interpretation of AUC values. It would be helpful to clarify whether higher AUCs truly reflect slower relaxation kinetics or are partly a consequence of reduced force generation. Normalizing AUC to tetanic force, or providing kinetic parameters such as t½ or τ of relaxation, would strengthen the functional analysis.

We acknowledge that the area under the relaxation curve (AUC) can be influenced by the magnitude of the tetanic contraction. In our analysis, the AUC values were already normalized to tetanic muscle force, so the reported differences reflect myotonia independent of variations in force generation. To make this clearer, we have modified the Y-axis label of the relevant graphs (Normalized Myotonia) and updated the Materials & Methods section to explicitly describe the normalization procedure: "Myotonia was derived from the area under the relaxation curve (AUC), normalised by tetanic force to account for variations in maximal contractile strength."

5. It would be valuable to assess whether the degree of Mbnl1/Mbnl2 silencing correlates with myotonia or loss of specific force (Fig. 1B–C). Even with limited numbers, plotting knockdown levels against functional measures and reporting simple correlation coefficients (non-parametric correlation coefficients) could clarify whether the variability observed reflects differences in silencing efficiency or stems from other uncontrolled factors.

We thank the reviewer for this insightful suggestion. To determine whether variability in functional parameters reflects differences in knockdown efficiency, we performed Spearman non-parametric correlation analyses between MBNL protein abundance, downstream splicing defects, and physiological measures. All analyses were conducted within the Mbnl KD cohort (data below).

Correlation analysis revealed no detectable association between MBNL1 protein levels and myotonia severity ($r = -0.14$, $P = 0.75$). MBNL2 protein levels exhibited a moderate negative correlation with myotonia ($r = -0.71$, $P = 0.058$), which did not reach statistical significance given the limited sample size. Correlations between MBNL protein abundance and specific force were minimal (MBNL1: $r = 0.071$; MBNL2: $r \approx 0.07$; $P > 0.4$).

We further examined whether downstream *Clcn1* mis-splicing predicts functional variability. *Clcn1* exon 7a inclusion levels were consistently elevated across Mbnl KD samples (PSI ≥ 0.5), markedly higher than in healthy muscle, where exon 7a inclusion is typically negligible. Exon 7a inclusion within this range is well established to reduce functional chloride conductance below the threshold required to prevent myotonia.

Importantly, however, myotonia severity is not expected to scale linearly with *Clcn1* exon 7a inclusion levels. Exon 7a inclusion levels do not directly quantify functional CLCN1 channel abundance or chloride conductance. Variability in myotonia magnitude may therefore arise from differences in total CLCN1 protein levels, channel stability, membrane localization, and additional modulatory factors influencing membrane excitability. Consistent with this interpretation, Spearman analysis did not reveal a

detectable correlation between exon 7a inclusion and myotonia severity (r = 0.095, P = 0.84; n = 8).

Altogether, these findings indicate that the molecular defects observed in Mbnl KD muscles are uniformly sufficient to permit myotonia, whereas variability in functional outcomes likely reflects the multifactorial nature of muscle excitability and contractile performance rather than differences in knockdown efficiency alone.

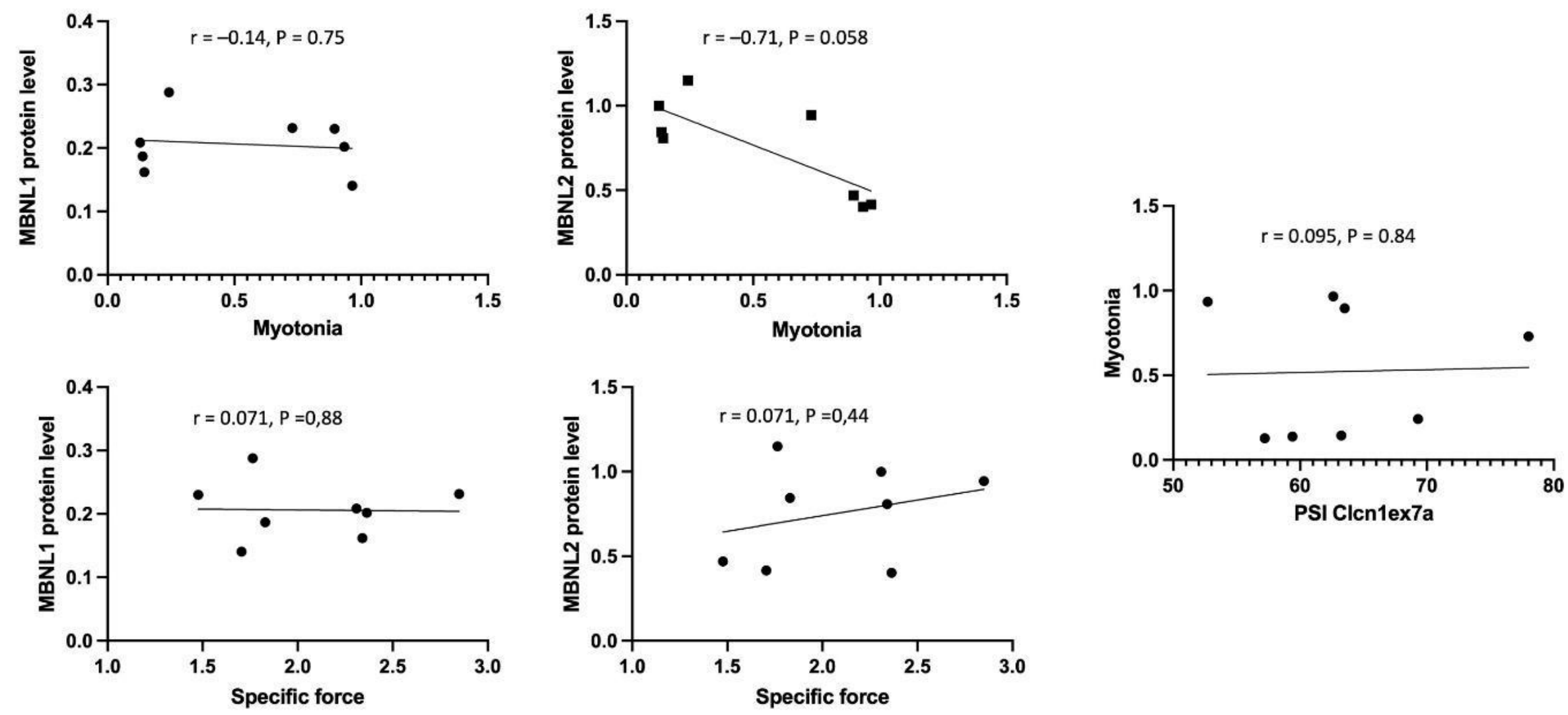

6. In Fig. 1E, the distribution of fibre cross-sectional areas is shown, and the text refers to increased heterogeneity and hypertrophy in Mbnl-KD muscles. However, no statistical analysis is provided to confirm whether the two distributions differ significantly. I recommend including an appropriate test to support the claim of altered fibre size distribution. Similar comment with Fig. 4B,C, E,F.

We performed statistical analysis of the fibre cross-sectional area distributions using a two-way ANOVA with Sidak's post hoc test. In Mbnl KD muscles, we observed significantly fewer percentage of fibres in the 750–1000 µm² and 1000–2000 µm² ranges, and significantly more fibres in the 2000–3000, 4000–5000, 5000–6000, and >6000 µm² ranges, confirming the presence of fibre hypertrophy and increased heterogeneity. These statistical analyses are now presented in the figures, providing clear confirmation of the reported changes in fibre size distributions.

Statistics were now also performed for the human samples (former figure 4, now figure 5). All figures have been updated accordingly to display the results of these statistical tests, thereby providing quantitative support for the conclusions drawn from the distribution analyses.

7. For Supplementary Fig. S2 and other comparisons, can the authors use a quantitative similarity metric to substantiate the developmental mapping beyond the UMAP visualization?

Uniform Manifold Approximation and Projection (UMAP) visualization are based on projection of high dimensional data in 2D space, thus there is indeed quantitative metric behind the visualization, including the calculation of nearest neighbors. Thus, the UMAP projection gives some information about the similarity, even if the distances are not informative *per se*.

8. The experiments described in panels 3E and 3H should be explained more effectively to enhance the readers' understanding. It is not entirely clear what is being represented in the corresponding schematics, as they are not genetic crosses nor molecular depictions of what is happening molecularly.

We agree that illustrations in panels 3E and 3H were not sufficiently informative and could be misinterpreted.

In response to the reviewer's comment and based on additional data, we have modified the figure and removed the schematics**.** Explanation of $Pax7^{DTA}$ model has been included in the result section.

"Next, we used $Pax7^{CreERT2;}$ $R26R^{DTA}$ mice, in which Cre-mediated activation of the diphtheria toxin A (DTA) chain in $Pax7^{+}$ cells results in the cell-autonomous ablation of muscle stem cells following tamoxifen treatment"

9. Fig. 4 appears to be presented descriptively (no statistical tests indicated in the legend). Given n=3 per group and the muscle mismatch with DM1-2, this is reasonable. However, if claims like correlations are retained in the text (e.g., "increase/correlation with repeat length"), please either tone them down as trends or provide patient-level statistics and state this explicitly in the legend.

We agree that, given the limited sample size (n = 3 per group) and the use of different muscles for patient DM1_2, the analyses presented in Fig. 4 were initially descriptive and should be interpreted with caution.

In response to this comment, we have now added patient-level quantitative data and statistical analyses for the parameters shown in Fig. 4. These data are presented at the individual donor level, and the corresponding statistics are explicitly reported in the figure panels.

10. Could the authors detect MBNL1 in central nuclei from the patient samples to establish parallelisms with the in vivo results? Indeed, the snRNA-seq already includes this information at the nuclear RNA level.

We analysed MBNL1 nuclear RNA expression across all clusters in the human snRNA-seq dataset (pseudobulk expression; Supplementary Fig. S12). MBNL1 shows a consistent downward trend in DM1 across myonuclear fibre types (Type I, IIa, IIx) and is also lower in several interstitial populations, but these differences do not reach statistical significance with n=3 controls vs n=3 DM1 donors. Importantly, MBNL1

transcripts remain detectable in all nuclear populations, including Cluster X, and Cluster X does not appear selectively depleted compared with other myonuclear types at the RNA level. This likely reflects the fact that MBNL1 transcript abundance does not capture functional MBNL1 availability, which in DM1 is primarily reduced through sequestration of the protein by CUG-expanded *DMPK* RNA rather than through reduced transcription. Thus, although MBNL1 RNA is present in Cluster X nuclei, its protein function is expected to be compromised, consistent with our model linking MBNL loss of function to impaired myonuclear maturation.

Importantly, Cluster X also displays markedly elevated *DMPK* expression (the source of the toxic CUG-expanded transcripts), supporting the interpretation that MBNL1 protein is functionally depleted in this population due to enhanced sequestration. This strengthens the parallel with our *in vivo* mouse model, where central/immature nuclei emerge in the context of MBNL loss of function.

11. In panel 4H it is unclear whether the data correspond to Ki67-positive nuclei, as indicated in the text, or to PAX7-positive nuclei, as shown in the figure. Globally, in this figure, it is not specified what each individual data point in the bar graphs represents. Also, the reported density of ~100 PAX7-positive nuclei per mm² appears too high if images are representative. Could the authors check whether these data are correct?

We have re-checked both the quantification and the associated text.

First, we confirm that the values shown in Fig. 5H (formerly referenced as Fig. 4H) correspond to PAX7-positive nuclei, as correctly indicated in the figure and legend.

We agree that ambiguity arose from imprecise wording in the Results section, which has now been corrected. The revised text reads:

“All three DM1 muscle samples exhibited increased numbers of nuclei positive for the proliferation marker Ki67, relative to controls (Fig. 5G,H). The number of Ki67PAX7-positive nuclei (per mm2) increased with repeat length (Fig. 5H). Co-staining of Ki67 and PAX7 showed an increased number of double-positive nuclei across all DM1 muscle specimens (Fig. 5I). Strikingly, there were Ki67-positive nuclei also detected within myofibres (Fig. 5J), and the majority of these were centralized (Fig. 5K,L).”

Second, regarding the apparent high density of ~100 $PAX7^{+}$ nuclei per mm², we confirm that these values are correct and arise from normalization of counts obtained within defined image areas. Quantification was performed on images covering 280 µm × 280 µm (0.0784 mm²). Scaling to nuclei per mm² yields the reported densities.

12. Because DM1 samples show both a shift toward oxidative fibres and larger fibre diameters, increases in peripheral/central myonuclei could partly reflect fibre-type composition and geometry. Please clarify whether nuclear counts were normalised for fibre size (e.g., perimeter- or area-normalised “nuclear density”) and, if possible, stratify or adjust counts by fibre type (see Suppl. fibre-typing) to decouple composition/size from disease effects.

To address this concern, we have now normalised nuclear counts to tissue area and added patient-level statistics showing the number of nuclei per unit area for both peripheral and central nuclei. These analyses are presented in Supplementary Fig. S8.

13. Fig. 5A–C (cluster abundance): In the per-sample UMAPs, cluster 4 is non-negligible in Ctrl_3 (closer to DM1_1 than expected), and cluster 2 appears larger in at least one DM1 sample than in Ctrl_3, whereas the text describes cluster-2 reduction as the clearest DM1 change. As conclusions currently rely mainly on these visualizations, please provide quantitative support at the donor level for clusters 2 and 4 and clarify the denominator used (all nuclei vs. myonuclei). Otherwise, these statements should be framed as qualitative trends given the small n and the muscle mismatch in DM1_2. Adding the quantitative support would substantially strengthen this and other claims similarly based on UMAP representations. The same is applicable to Fig. S5.

To address this point, we have now added donor-level cluster quantification to the Supplementary Information (Supplementary Fig. S10).

This analysis reports, for each donor, the proportion of nuclei assigned to each cluster, using all myonuclei as the denominator (this is now explicitly stated in the figure legend). The donor-level quantification confirms that cluster 4 (Cluster X) is present at a non-negligible level in Ctrl_3, and that cluster 2 (type IIx myonuclei) displays marked donor-specific variability, including a relatively high representation in one DM1 sample.

In light of these quantitative data, and given the small cohort size (n = 3 per group) as well as the muscle-type mismatch in DM1_2, we agree that differences in cluster abundance should be interpreted as qualitative trends rather than statistically supported shifts. We have therefore revised the Results text accordingly to avoid overinterpretation of the UMAP visualizations.

14. This text is apparently not fully accurate according to the graph shown in Fig. 5D and supplementary figure S6: "Fast-twitch nuclei (types IIa and IIx) showed increased DMPK only in patients 2 and 3, coinciding with fibre-type-specific abnormalities, including fibre size variability. Outside of slow fibres, DMPK was notably enriched only in Cluster X, but not in canonical MuSCs". In any case, a statistical test is necessary to support these conclusions. Also, define the plotting conventions (e. g. colour scale and line/dot thickness), and clarify whether values are averaged per donor or pooled, because this representation is not at all easy to follow. According to Figure S6, CNT3 displays a similar level of DMPK expression as in the patient samples, which does not align with the authors' statement at line 325. In addition, in cluster X of Figure 5D, no expression is observed in the controls, whereas in the supplementary figure, one control shows a level of expression comparable to DM1. The authors should clarify precisely what is being represented in these panels.

We agree that our original wording and the previous visualization made the interpretation of *DMPK* expression insufficiently clear.

In response to this comment, Fig. 5D (now 6D) was redesigned: we replaced the pooled violin/feature-style representation with donor-level boxplots (each dot = one donor) showing *DMPK* expression per donor and cell type/cluster. This makes it explicit that

values are not pooled across donors, and directly visualizes donor-to-donor variability (including Ctrl_3).

We added statistical testing at the donor level (non-parametric tests on donor-level summaries) to support (or refute) statements about *DMPK* enrichment across clusters.

We clarified plotting conventions in the figure legend (what each dot represents, what summary is shown, and the scale/normalization used).

To avoid confusion and redundancy, and following another reviewer's request to simplify the presentation, we removed Supplementary Fig. S6 (which was showing a different ordering/format and contributed to the apparent inconsistencies).

15. Fig. 5E (bulk validation of "Cluster X" signature): The panel works well as an external validation with an apparent severity gradient. Please (i) resolve the control-sample discrepancy (legend says 9 controls, Methods 11), and (ii) given that bulk signals can reflect tissue composition, it would be helpful to note how this was considered or include this possibility as a limitation of the approach.

We apologize for the inconsistency regarding the number of controls used. Fig. 5E includes 11 controls, we have corrected the figure legend accordingly. We agree that bulk RNA-seq reflects the composite signal of all cell types present and therefore may be influenced by variation in fibre-type composition or non-myogenic content. While our goal was not to infer Cluster X abundance, but rather to determine whether Cluster X marker genes exhibit consistent dysregulation patterns across independent datasets, we acknowledge that bulk RNA-seq cannot distinguish between cell-intrinsic regulation and tissue composition effects. We have now added a statement in the Discussion explicitly noting this limitation: "Because bulk RNA-seq reflects the combined transcriptional output of all cell types present in the biopsy, the increased abundance of Cluster X markers in the independent cohort may partly reflect differences in tissue composition (e.g., fibre-type proportions or non-myogenic content) rather than changes within a specific myonuclear population. Thus, while the bulk data provide supportive external validation, they should be interpreted as indicative rather than definitive evidence for Cluster X expansion."

16. Fig. 5F and Supplementary Fig. S7 qualitatively support increased MYOG/MYF6 and broad ANKRD1 upregulation in DM1 myonuclei. To substantiate these statements, please provide quantitative metrics focused on the biological unit (the donor).

We agree that statements based on single-nucleus visualizations should be supported by donor-level quantification, particularly given the small cohort size.

To address this, we now provide per-donor quantitative summaries of *MYOG*, *MYF6*, and *ANKRD1* expression in myonuclei, shown as donor-level plots in the revised Supplementary material (Supplementary Fig. S13). Specifically, for each donor we computed cluster-level/pseudobulk expression values and plotted these as individual data points (one point per donor), allowing direct comparison between control and DM1 samples at the level of the biological replicate.

These donor-level analyses confirm the qualitative trends observed in Fig. 5F and the original Supplementary Fig. S7: *MYOG* and *MYF6* are increased in DM1 myonuclei, and *ANKRD1* is broadly upregulated across myonuclear fibre-type clusters. Given the limited n, we describe these as donor-consistent trends, and where applicable we report non-parametric donor-level statistics in the figure panels/legend.

17. Supplementary Fig. S8 shows bulk expression of ANKRD1/CDKN1A/MYF6 stratified by dorsiflexion strength (9 controls, 43 DM1), but no donor-level statistics or correlation coefficients are provided. If the text states that ANKRD1 and MYF6 are significantly upregulated and correlate with weakness, please include the corresponding donor-level tests and effect sizes, or rephrase as a descriptive trend.

Done (now Supplementary Figure S14).

18. Please state the unit of aggregation for the violins (pooled nuclei vs. per-donor averages within each cluster). Define the expression metric/scaling used and any threshold for calling a nucleus “expressing" a given gene.

Done.

19. The manuscript reports increased Ki67^{+} nuclei (peripheral and central) and elevated p21/CDKN1A in central myonuclei. To avoid misinterpretation, please clarify whether Ki67 and p21 are mutually exclusive at the single-nucleus level (cycling vs. post-fusion/arrest). If possible, include a double-immunostaining or a co-localization/overlap quantification; otherwise, state explicitly that these markers were assessed separately and reflect different stages (MuSC proliferation vs. post-fusion myonuclear arrest).

We agree with the reviewer that this is an important point, because Ki67 and p21/CDKN1A are commonly interpreted as markers of distinct cell-cycle states (cycling vs. post–cell-cycle-exit/arrest), and we want to avoid any suggestion that we are claiming they co-occur in the same nuclei without evidence.

In our study, Ki67 and p21 were assessed in separate stainings, on different sections/assays, and we therefore cannot conclude whether they are mutually exclusive at the single-nucleus level in human DM1 muscle. We have now made this explicit in the text to prevent misinterpretation: “Ki67 and p21/CDKN1A stainings were performed on separate sections and were not co-localized in the current study; therefore, we cannot determine marker overlap at single-nucleus resolution. We therefore interpret Ki67 as marking proliferating MuSCs (pre-fusion), whereas p21/CDKN1A marks post-fusion myonuclei in a non-cycling/arrested state.

20. The link between the murine Meg3^{+}/Ankrd1^{+} myonuclear states (Mbnl-KD) and the human “Cluster X” is presented largely on qualitative grounds (visual overlap and a few shared markers). It remains unclear whether Cluster X is a direct counterpart of the mouse states or a related but distinct transitional program. Because several translational inferences hinge on this connection, please make explicit your hypothesis (counterpart vs. analogous state), summarize

the evidence that supports it, and delineate its limits. An additional effort to integrate results from both experimental systems would improve the quality of the paper.

We agree with the reviewers and we have added the following three comparisons:

1) We have overlapped the snRNA-seq data from the human and mouse data, where we can largely appreciate that the *Ankrd1+* cells broadly overlap with the totality of the DM myonuclei, including but not limited to Cluster X (Supplementary Fig. 15).

2) We compared the list of marker genes that distinguish the *Meg3+* myonuclei, the *Ankrd1+* myonuclei and the Cluster X myonuclei from the DM patients and found similar percentage of overlap between the two populations (32/236 *Meg3+* ~13% 205/1666 *Ankrd1+* ~12%). (Line 510 ff)

3) The list of genes which overlap between the two different human and mouse clusters are listed in (Supplemental Table T9).

Together, these analyses support that human Cluster X is analogous to the mouse *Ankrd1*$^{+}$ transitional myonuclear program, rather than a strict one-to-one counterpart. The overlap is partial and likely reflects shared stress/immaturity pathways across different myonuclear contexts; therefore, we limit cross-species inferences to the shared signature rather than asserting direct equivalence.

21. In the Methods section, the authors state that data were first tested for normality, and if the distribution was not normal, Welch's test or the Mann–Whitney test was applied when comparing two groups. However, in the figure legends, all comparisons are indicated as being performed with t-tests or ANOVA. Could the authors please confirm whether, in all cases, the data from all groups passed the normality test?

For mice experiments, we confirm that all datasets passed the normality test (Shapiro–Wilk test)**.** Accordingly, comparisons were appropriately performed using paired t-tests (as requested in point #3) or ANOVA as indicated in the figure legends. The Methods section has been updated to clarify this point.

22. If the aim is to establish a parallel between the findings in mice and the patient biopsies, could the authors clarify why they used tibialis anterior muscle for the mouse experiments? The tibialis is relatively enriched in type I (oxidative) fibers, whereas most patient biopsies are taken from biceps brachii, which is predominantly composed of type II (fast, glycolytic) fibers. Wouldn't the extensor digitorum longus (EDL), which is type II–rich, represent a more appropriate counterpart for comparison? Please justify/discuss this choice.

We acknowledge that the tibialis anterior (TA) muscle is relatively more oxidative than the biceps brachii, which is predominantly composed of type II fibres. Our choice of the TA muscle was primarily motivated by the fact that the physiological setup for *in situ* assessment of muscle force and myotonia in our platform is optimized for this muscle, ensuring reproducible and standardized recordings. Regarding human samples, the availability of patient biopsies often constrains direct fibre-type matching across species. We agree that myofibre composition could influence the magnitude or profile

of Mbnl-related phenotypes. This point is now discussed in the Discussion section to acknowledge its potential impact on the interpretation of mouse–human comparisons.

To address this explicitly, we have added the following statement to the Discussion: "Our snRNA-seq analysis also revealed differences between human and mouse datasets. These discrepancies may partially reflect the distinct muscle origins analysed in each species, with mouse data derived from TA muscles and human samples obtained from biceps brachii biopsies. Variations in fibre-type composition and muscle-specific transcriptional programs could contribute to these species-specific patterns and should be considered when interpreting cross-species comparisons."

Minor points

1. Please use knockdown in the abstract as it is more accurate, given that 20% and 50% of Mbnl1 and Mbnl2, respectively, of gene function remains after the experimental manipulation. Also, use the official name in the keywords and introduction (muscleblind-like splicing regulator, the same in the introduction).

Thank you for pointing this out, to be accurate we have updated the abstract to use "knockdown". Regarding the official name: MBNL is officially listed as muscleblind-like protein (UniProt). To avoid any misunderstanding we added now "(also known as muscleblind-like splicing regulators)" to the introduction at the first mention of MBNL.

2. The introduction needs to incorporate new ideas regarding the DM1 pathogenesis pathway. Papers like PMID: 31116797 and PMID: 39383229 already support active repression of MBNL1 expression, in addition to the classic CUG RNA sequestration, as a cause of MBNL1 depletion.

We agree that recent work supports additional mechanisms contributing to MBNL1 depletion, including post-transcriptional repression of MBNL1 by specific microRNAs and isoform/partitioning changes that reduce functional MBNL protein, complementing the classic sequestration model. We have revised the Introduction to incorporate these points and cite supporting studies:

"Beyond sequestration, additional mechanisms lower effective MBNL1 levels in DM1. Patient-derived myogenic models show repeat-dependent dysregulation of MBNL1/2 splicing, with increased inclusion of specific exons and imbalanced isoform pools that reduce functional protein and alter nuclear–cytoplasmic partitioning already at the myoblast stage[19]. These data indicate that MBNL insufficiency starts upstream of mature fibres. Moreover, microRNA-mediated repression of MBNL1—most notably by miR-23b and miR-218, whose levels correlate with CTG repeat size—actively suppresses MBNL1 protein; antimiR treatment both restores MBNL1 and improves DM1 molecular and cellular phenotypes[20]. Together, these findings support a multi-hit model in which sequestration, isoform/partitioning changes, and active post-transcriptional repression converge to deplete functional MBNL1 in DM1."

3. Please discuss the mechanistic basis for the apparent increase of DMPK in myonuclei with an immature/transitional profile (“Cluster X”).

> The apparent enrichment of *DMPK* transcripts in myonuclei with an immature/transitional profile (Cluster X) likely reflects a combination of cellular origin, transcriptional state, and nuclear RNA handling, rather than a simple increase in *DMPK* gene activation *per se*.
>
> One plausible mechanism relates to the developmental status of these nuclei. Cluster X exhibits transcriptional features reminiscent of recently incorporated or incompletely matured myonuclei, including expression of genes associated with early myogenic programs and impaired terminal maturation. During early myogenesis, *DMPK* expression is relatively high, and persistence of this developmental expression pattern in immature myonuclei could contribute to elevated *DMPK* transcript levels in this population.
>
> In addition, Cluster X nuclei may represent nuclei that have recently fused into myofibres and have not yet fully adopted the transcriptional and post-transcriptional regulatory landscape of mature myonuclei. Such nuclei may display altered RNA processing, nuclear retention, or turnover, potentially leading to accumulation of DMPK transcripts within the nucleus. This interpretation is consistent with the observation that *DMPK* enrichment is particularly evident at the nuclear RNA level, as assessed by snRNA-seq.
>
> We currently interpret elevated nuclear DMPK in Cluster X as reflecting a transitional myonuclear state with altered RNA handling and/or incomplete maturation, rather than definitive evidence of increased DMPK transcription per se. Cluster X is characterized by an immature transcriptional signature and high DMPK at the nuclear RNA level, consistent with the possibility of increased nuclear retention/stability of DMPK transcripts in these nuclei. We now explicitly present this as a hypothesis and discuss alternative explanations (e.g., cell-state composition and technical factors inherent to nuclear RNA measurements).

4. Please add a reference supporting the muscle specificity of the Spc5.12 promoter. At line 116 the authors state that AAV9 was used, whereas in the Materials and Methods section (line 545) they refer to AAV9/2. This discrepancy should be clarified.

> The virus used throughout the study was AAV2/9, in which the AAV2 genome (containing the expression cassette and ITRs) is packaged into an AAV9 capsid, conferring muscle tropism. The mention of “AAV9” in the main text has been clarified to specify AAV2/9 for consistency. A reference supporting the muscle specificity of the Spc5.12 promoter has also been added in the text.

5. In Fig. 1G, some control fibres are reported as containing only a single nucleus. Since adult myofibres are multinucleated, I assume this reflects nuclei intersected in a single transverse section rather than the total nuclear content of the fibre. It would be helpful to clarify this point in the legend or methods to avoid misinterpretation.

The observation of fibres containing a single nucleus indeed reflects nuclei intersected in a single transverse section, rather than the total nuclear content of the myofiber. We have clarified this point in both the Methods section to avoid any potential misinterpretation.

Methods - Muscle histopathology:

"For quantification of myonuclei per fiber, nuclei were counted in single transverse sections; thus, values represent the number of nuclei intersected in the section rather than the total nuclear content of each myofiber."

6. In Fig. 1H, the heatmap of splicing events appears to be based on three biological replicates per group (three columns shown), whereas the legend states that at least five mice were used in each group. It would be helpful to clarify the exact sample size for the RNA-seq analysis, to avoid confusion with the numbers reported for histological and functional experiments.

The reviewer is correct, the RNA-seq analysis was performed on three biological replicates per group**.** We have corrected the figure legend accordingly to ensure consistency with the data presented and to avoid confusion with the sample sizes used for histological and functional experiments.

7. Please provide the parameters used for clustering and UMAP projection, as these choices can markedly influence cluster separation.

We have now clarified the analysis workflow and reported the key parameters used for dimensionality reduction, clustering, and UMAP projection in the Methods section. Briefly, single-nucleus datasets were processed using Seurat v5 following quality control, doublet removal, and normalization/integration using the SCTransform framework. Clustering and UMAP embeddings were generated from the integrated latent space using a consistent analysis pipeline, and the specific settings used are now explicitly described to ensure reproducibility.

8. For clarity, it would be useful to add a short statement in the figure legend or Methods indicating that the UMAP plots represent a 2D projection of nuclei based on transcriptomic similarity. This would help non-specialist readers interpret the clustering.

The first legend for a figure introducing a UMAP plot (Figure 2) now includes the following description: "Uniform manifold approximation and projection (UMAP) plot of nuclei from Ctrl and Mbnl KD TA muscles. Each point represents a single captured nucleus, whose position on the graph is determined by the ensemble of all detected genes. The colours identify different nuclear populations derived from clustering analysis."

9. In Fig. 2B, the subclustering is described as including "all the myonuclei," but it is not explicitly stated which clusters from Fig. 2A were considered myonuclei. For clarity, please indicate whether this refers to the fibre type clusters (e.g., type I, IIa, IIx) and confirm that other mononucleated populations such as MuSCs or FAPs were excluded.

We apologize for the confusion in the original text. This has now been clarified explicitly in the text. Indeed, only the II b/x myonuclear clulsters were included in the subclustering analysis, without the other populations.

10. Please define DTA at first mention, noting that Cre activation drives cell-autonomous ablation of $Pax7^{+}$ cells. This will aid readers unfamiliar with the abbreviation.

We have now defined DTA (diphtheria toxin A) at its first mention in the text and clarified that Cre activation drives cell-autonomous ablation of $Pax7^{+}$ cells, to aid readers unfamiliar with the abbreviation: "Next, we used $Pax7^{CreERT2;}$ $R26R^{DTA}$ mice, in which Cre-mediated activation of the diphtheria toxin A (DTA) chain in $Pax7^{+}$ cells results in the cell-autonomous ablation of muscle stem cells following tamoxifen treatment."

11. The Supplementary Fig 3 is appropriate to document MuSC ablation, but the legend mentions Mbnl1/2 qPCR while the y-axis reads "Relative Pax7 mRNA"; please correct the target gene in the legend.

We apologize for the oversight and have corrected the legend of Supplementary Figure referring to Pax7 mRNA so that the target gene is accurately indicated as Pax7 mRNA, in agreement with the Y-axis labelling.

12. In Fig. 3B, the y-axis and legend indicate "percentage of myonuclei positive for EdU," yet panels C and D also quantify EdU^{+} myonuclei (central vs. peripheral) and show values of zero in controls. This seems inconsistent, since the total in B should equal the sum of C and D. Please clarify precisely what population is quantified in panel B versus panels C–D (e.g., satellite cells vs. myonuclei, or different normalization), to avoid confusion.

We thank the reviewer for pointing this out. Indeed, panel 3B contains an error. The values should represent the sum of panels C and D; the mistake occurred because the data were not converted to percentages, leading to the incorrect display of 0.0. This has now been corrected.

13. In Fig. 3F and others (e.g. Fig. 4J), the representative images are presented at a very small scale, making it difficult to appreciate EdU signal or nuclear localization. Enlarging the panels or providing zoomed-in views would improve clarity.

The representative images in Fig. 3 have been enlarged, and zoomed-in views have been included for a better visualization. We did also provide zoom-ins now for figure 4J (now 5J).

14. Please discuss this sentence "In patient DM1_3, the highest number of central nuclei was detected, with almost all fibres presenting with central nuclei (Fig. 4C/D)" because there is no correlation with a functional parameter like ambulatory capacity, which is preserved in this patient.

We agree that the high prevalence of centrally located nuclei observed in patient DM1_3 does not correlate with functional measures, such as ambulatory capacity, which is preserved in this individual. This underscores the fact that central nucleation alone is not a direct predictor of clinical severity or muscle function in DM1.

One important factor that may contribute to this apparent discrepancy is the muscle origin of the biopsies. Muscle biopsy samples from patients DM1_1 and DM1_3 were obtained from the biceps brachii, whereas patient DM1_2 underwent biopsy of the quadriceps femoris. Differences in muscle group, fibre-type composition, mechanical load, and usage are well known to influence myonuclear organization and adaptive responses. Thus, the higher prevalence of central nuclei in DM1_3 may reflect muscle-specific or local remodeling processes rather than overall disease severity or functional impairment.

15. Correct "bule" for "blue" in Supplementary Fig S5.

Thank you for noticing. We corrected this.

16. Please state whether mutant DMPK or both alleles are quantified in Fig. 5.

The *DMPK* expression values shown in figure 5 reflect the combined signal from both the wild-type and mutant *DMPK* alleles, as single-nucleus RNA sequencing (snRNA-seq) quantifies total *DMPK* transcripts without distinguishing between allele-specific sequences. We have now explicitly stated this in the figure 5 legend: "(representing the combined signal from both the wild-type and mutant alleles)"

17. Whereas DMPK levels are shown in the order "CTR-DM1" in Fig. 5D they are shown reversed in Suppl S6. Please show them in the same order to improve readability.

Fig. 5D (now 6D) was redesigned: we replaced the pooled violin/feature-style representation with donor-level boxplots (each dot = one donor) showing *DMPK* expression per donor and cell type/cluster. This makes it explicit that values are not pooled across donors, and directly visualizes donor-to-donor variability (including Ctrl_3).

To avoid confusion and redundancy, and following another reviewer's request to simplify the presentation, we removed Supplementary Fig. S6 (which was showing a different ordering/format and contributed to the apparent inconsistencies).

18. Line 344 should refer to Fig. 5F, instead of E.

We corrected this.

19. In Fig. 6A, each cluster×gene tile appears split into multiple vertical bars. Please specify what each sub-bar represents, the ordering (Ctrl_1–3, DM1_1–3), and the expression metric/scaling used. This will help readers interpret donor-level consistency rather than pooled values.

We have updated the figure legend to improve clarity: “Each column represents a single nucleus, organized by cluster. Rows correspond to genes, and color intensity reflects scaled gene expression levels.” (now Fig. 7A)

20. No data in the paper seems to support this sentence "we also propose additional mechanisms such as impaired nuclear migration due to altered nucleo-cytoskeletal integrity", which should be rewritten as state of the art, not a new proposal arising from the paper.

We thank the reviewer for this important comment and agree that the original wording could be misinterpreted as proposing a new mechanism arising directly from our data, which was not our intention.

21. In light of previous comments, note that the statement in line 482 of the paper must be toned down unless it is statistically backed.

The statement has been revised to reflect that this observation represents a trend apparent across patient groups, rather than a formally tested statistical correlation. We have softened the language accordingly to avoid overinterpretation while maintaining the observation’s descriptive value: “Although this analysis was limited to a small number of DM1 muscle biopsies, Cluster X marker gene expression tended to increase with clinical disease severity in the independent cohort of 43 DM1 patients. While this trend is consistent with a potential link between immature myonuclear states and disease progression, formal statistical validation will require larger sample sizes and uniform clinical data.”

22. Please include the actual sequences targeted by shRNAs used in the AAV (lines 525-529), for better reproducibility of results reported.

The AAV-shRNA constructs targeting Mbnl1 and Mbnl2 were designed based on previously published shRNA sequences (Wang et al. 2012). The exact sequences are now provided in the method section.

Mbnl1 shRNA sequence: GAAGTATGTAGAGAGTTTCAA

Mbnl2 shRNA sequence: CGGCTATTAGCTTTGCTCCTT

LacZ shRNA sequence : AAATCGCTGATTTGTGTAGTC

23. Please confirm whether myotonia was measured under constant temperature conditions, as this may impact measurements.

We confirm that myotonia and *in situ* force measurements were performed under controlled temperature conditions, with the mice maintained on a temperature control-warm plate to ensure consistent physiological conditions. We added this to the Material and Methods description now: “Mice were anaesthetised with pentobarbital sodium (60 mg/kg, intraperitoneally) and kept on a temperature-controlled warm plate to ensure stable physiological conditions during the experiments.”

24. The Methods state that nuclei isolation was performed “as previously described, with some modifications optimized for the muscle,” but only the addition of BSA is explicitly mentioned. For reproducibility, it would be important to clearly specify all modifications introduced relative to the published protocol.

> We have changed the wording of the methods section to better highlight the modification. Indeed, all the different modifications were already marked related to the original protocol (BSA, cell strainers and no gradient centrifugation).

25. Please clarify in the Methods how fiber area quantification was performed (e.g., software, number of fibers analyzed, blinding).

> For mice analysis, fibre cross-sectional areas were quantified using MuscleJ on all fibres from a single transverse section of the TA muscle. Analyses were performed blinded to genotype or treatment group to ensure unbiased measurements. These details are now included in the Material and Method section.

26. The sequences of the primers used are not provided. For reproducibility and transparency, please include the exact primer sequences in the corresponding section.

> Primers sequences have now been added to the Material and Methods section.

Reviewer #3 (Remarks to the Author):

Todorow et al. show the muscle stem cells (MuSCs) are activated and fuse to myofibers when Mbnl 1/2 are reduced in myofibers, a common feature of myotonic dystrophy. By using mouse genetic models and snRNA sequencing they showed that MuSCs contribute new nuclei into Mbnl 1/2 KD myofibers and they make interpretations that these nuclei do not immature transcriptional state. By using snRNA sequencing and histology from human muscle biopsies of healthy and MD patients, Todorow et al. showed that MuSCs are also activated and found the presence of an immature myonuclear cluster, similar to what they observed in the Mbnl 1/2 KD muscles. The snRNAseq data provide a good resource for the muscle community. There are not major concerns about the technical validity of the data. However, I am not able to support publication because the interpretations are invalid and not supported by the data, there is a major disconnect between the themes in the manuscript (MuSCs, snRNAseq in mouse, snRNAseq in human), and the overall premise of the paper is not communicated well.

1. The main premise of the paper as stated in the abstract is that there are questions whether DM1 maintains its regenerative potential or shows MuSC defects. Based on my review of the literature, it already known that there is regeneration and MuSC defects. It seems obvious that MuSCs contribute to the central nuclei in DM1 muscle. The authors should communicate more clearly about the specific questions in DM1 related to MuSCs and how their study contributes.

We agree that the novelty and specific scope of our study were not articulated with sufficient clarity in the original version of the manuscript. We agree that prior studies,—many of which we have contributed to,—have reported abnormalities in the myogenic compartment in DM1, including impaired proliferation, differentiation, and premature senescence of DM1-derived myoblasts *in vitro* (Furling et al., 2001; Bigot et al., 2009; Beffy et al., 2010; Renna et al., 2017; Conte et al., 2023), as well as altered satellite cell cell numbers in DM1 patient muscle (Thornell et al., 2009). However, these studies primarily address myoblast behavior *ex vivo*, rather than directly examining the *in vivo* contribution and fate of MuSCs in adult DM1 muscle fibers.

To date, and to our knowledge, no study has directly demonstrated the cellular origin of centrally located nuclei in adult DM1 muscle *in vivo*. Central nucleation is often interpreted as evidence of regeneration following necrotic fiber loss, yet overt necrotic degeneration accompanied by immune cell infiltration are rarely observed in DM1 muscle (Thornton 2014). Thus, whether MuSC activation in DM1 reflects canonical degeneration–regeneration cycles, compensatory fusion into existing fibers, or aberrant post-fusion states has remained unclear.

Our study addresses this gap by (i) directly testing MuSC contribution to myonuclei in vivo using tracing and ablation of $Pax7^+$ MuSCs in an adult-onset, myofiber-restricted Mbnl1/2 knockdown model, and (ii) identifying a transcriptionally distinct population of immature myonuclei in human DM1 muscle by single-nucleus RNA sequencing. These data demonstrate that MuSCs actively fuse into adult DM1 muscle fibers independent of overt necrotic degeneration and give rise to nuclei with altered maturation states, a phenomenon that has not previously been demonstrated *in vivo*.

We have revised the Abstract and Introduction to more clearly distinguish prior findings on myoblast defects from the specific unresolved questions addressed here, namely the *in vivo* behavior and fate of MuSC-derived nuclei in adult DM1 muscle.

2. Line 29-30: what are the data that central nucleation is independent of degeneration? Some negative effects must be occurring in myofibers since the manipulation is myofiber-specific. The authors have not assessed what is happening in myofibers.

Indeed, we agree and our intent was not to suggest that myofibres are unaffected by Mbnl depletion, but rather to distinguish the observed central nucleation from classical degeneration–regeneration cycles involving myofibre necrosis, fibre loss, and replacement. While we did not comprehensively assess all aspects of myofibre pathology, several independent observations argue that overt degeneration–regeneration is not the primary mechanism underlying central nucleation in this model:

i) We did not detect histological hallmarks of myofibre necrosis or widespread regeneration, including fibre loss, inflammatory infiltration, or extensive regeneration-associated remodelling.

ii) The total number of myofibres remained unchanged, which is inconsistent with repeated cycles of fibre degeneration and replacement.

iii) Central nucleation was strongly reduced upon ablation of Pax7^{+} MuSCs (Pax7-DTA), indicating that MuSC fusion is required for the accumulation of centrally located nuclei. Importantly, if central nucleation were driven primarily by necrotic degeneration, MuSC ablation would be expected to result not only in loss of central nuclei but also in unresolved fibre loss (lower total number of fibres) and increased fibrotic replacement; such changes were not observed.

iv) Fibre size distributions and myonuclear analyses are most consistent with nuclear accretion into pre-existing fibres rather than the formation of new fibres following degeneration.

With respect to the reviewer's comment that "the authors have not assessed what is happening in myofibers," we note that MBNL depletion induced clear myofibre-intrinsic dysfunction, including myotonia, fibre splitting, altered force generation, fibre-type switching, and transcriptional changes detected by RNA sequencing. These findings demonstrate that myofibres are functionally and molecularly affected in this model; however, we interpret these changes as reflecting myofibre stress or dysfunction rather than overt necrotic degeneration driving central nucleation. We acknowledge that our original wording overstated the conclusion by implying complete independence from degeneration and we have revised the text accordingly

3. Line 34-36: This is a major over-interpretation and based on the literature is likely to be incorrect. I don't see any data indicating that the cells fusing into DM1 myofibers give rise to 'immature' nuclei. It is just as likely that these are nuclei already in the myofiber that exhibit this signature. Assessing gene expression in the DM1 model after MuSC ablation could be one way to test this idea.

We thank the reviewer for this comment. To determine whether *Meg3* and *Ankrd1* reactivation depends on MuSC-derived nuclei, we performed snRNA-seq on muscles from Pax7-knockdown mice 10 weeks after Mbnl KD ($Pax7^{DTA}$ Mbnl KD) and compared them to Mbnl KD in $Pax7^{CreERT2}$ control mice ($Pax7^{CreERT2}$ Mbnl KD). We first confirmed using this approach that DTA efficiently reduces the $Pax7^{+}$ cell population by 85% as well as the number of centralized nuclei. Notably, Pax7 depletion also resulted in a partial reduction of the *Meg3*$^{+}$/*Ankrd1*$^{+}$ nuclear subpopulation (Figure 4G-H ; Supplementary Fig S6). This suggests that although $Pax7^{+}$ cells contribute, they are not the only source of these new populations. From this experiment, it is not possible to determine if the reactivation occurs in the pre-existing nuclei, or if they are derived from other non-Pax7 derived fusing cells. However, in the original experiment, we observed that both *Meg3*$^{+}$ and *Ankrd1*$^{+}$ populations express *Mbnl1*, suggesting that they indeed derive from a cell type that was not targeted by the AAV virus for knockdown.

4. The statement that this immaturity is 'likely' due to RNA effects on transcriptional reprogramming is also not substantiated. It can be appropriate to include these type of comments in the discussion but not in the abstract.

We agree that the statement implying that myonuclear immaturity is *"likely"* caused by RNA-mediated effects on transcriptional reprogramming was too speculative for the Abstract and not directly demonstrated by our data.

To address this, we have removed causal language from the Abstract and limited mechanistic interpretations to the Discussion, where they are clearly framed as hypotheses rather than conclusions. The Abstract now describes the observations in a strictly data-driven manner, focusing on the identification of immature/transitional myonuclear states without attributing a specific underlying mechanism.

Specifically, we revised the Abstract as follows:

<u>Before:</u>

"These findings imply that MuSCs in DM1 contribute to muscle maintenance and adaptive fusion by integrating into myofibres, but give rise to immature myonuclei, likely due to toxic RNA effects on transcriptional reprogramming."

<u>Revised:</u>

"Together, these findings indicate that MuSC-mediated fusion drives myonuclear remodeling in DM1 muscle, giving rise to centrally located myonuclei with altered transcriptional states."

In the Discussion, we now explicitly state that RNA toxicity and MBNL loss-of-function are potential mechanisms, consistent with existing literature, but that causality cannot be inferred from the present data.

5. Line 36-37: I don't think MuSCs have been previously overlooked in DM1 pathology. In fact there was a Nature Communications paper (Conte et al 2023) that showed senolytics can restore myogenesis in DM1. Also, what is the 'role' of MuSCs that this current work has defined?

We agree that MuSC involvement in DM1 pathology has been previously documented and was not overlooked. It also appears that the Nature Communications paper (Conte et al) has unfortunately been incorrectly edited and did not appear in the references, even though the results of this work are mentioned in the introduction. A substantial body of work, including studies from our own groups, has reported intrinsic abnormalities in the myogenic compartment in DM1, notably impaired proliferation, differentiation, and premature senescence of DM1-derived myogenic progenitors in vitro (Furling et al., 2001; Bigot et al., 2009; Beffy et al., 2010; Renna et al., 2017). In this context, the study by Conte et al. (Nature Communications, 2023) is fully consistent with earlier findings and extends them by demonstrating that pharmacological clearance of senescent myogenic progenitor cells can restore myogenic potential, thereby establishing progenitor cell senescence as a therapeutically relevant component of DM1 pathology.

However, these studies address the intrinsic state and regenerative competence of MuSCs and myoblasts, rather than defining their in vivo contribution within adult DM1 muscle fibers. Specifically, Conte et al. focus on myogenesis in vitro and on the regenerative capacity of expanded DM1 myoblasts following transplantation into acutely injured mouse muscle, but do not examine MuSC behavior, fusion, or nuclear fate within adult DM1 muscle in situ.

The role of MuSCs defined in the present study is therefore distinct and complementary: using an adult-onset DM1 mouse model, we demonstrate in vivo that MuSCs actively fuse into existing myofibres, that this fusion is required for the accumulation of centrally located nuclei, and that the resulting MuSC-derived myonuclei exhibit an immature or altered transcriptional identity. Importantly, we further demonstrate the presence of this altered myonuclear population in human DM1 muscle using histological analyses and single-nucleus RNA sequencing.

6. The model used to knockdown Mbnl1/2 in myofibers is perplexing because DM1 is also caused by Mbnl1/2 defects in MuSCs. Based on comments throughout the paper (such as Lines 211-212), are the authors thinking that the Mbnl depletion is occurring in MuSCs? This can be directly tested since AAVs can transduce MuSCs albeit at lower levels than myofibers. I don't see any direct data to support this and based on their system I was thinking that depletion occurs mostly in myofibers, causing defects in myofibers leading to MuSC activation and fusion.

In our Mbnl1/2 knockdown model, the AAV-mediated silencing is largely restricted to myofibres and does not significantly affect MuSCs (*see response to Referee #1*). This is due to two key factors: (i) the SPc5-12 promoter used in the constructs is muscle-specific and largely inactive in proliferating or quiescent MuSCs, becoming transcriptionally active only in post-mitotic myonuclei; and (ii) AAV serotype 2/9 transduction of MuSCs is very inefficient (<1–2%), even at high intramuscular or systemic doses (Boutin et al., Nat Commun 2010; Kimura et al., Mol Ther 2014; Arnett et al., Mol Ther Nucleic Acids 2014). Together, these factors make substantial direct delivery of the silencing cassette to MuSCs improbable. Finally, our single-nucleus RNA-seq data support a muscle fibre–restricted effect. The decrease in *Mbnl1* transcript was observed in myonuclei Type IIx/a and Type IIb fibres while no downregulation was observed in MuSC cells (Supplementary Fig. S3 A).

In contrast, in DM1 patients, the causal pathogenic factor (toxic CUG-expanded *DMPK* transcripts) is found in both myofibres and MuSCs. Therefore, MBNL depletion could occur in both cell types depending on the level of *DMPK* expression, which may contribute to the observed defects.

7. Lines 104-106: It is unclear whether the data suggest impaired myonuclear maturation because the signature they are detecting could be from recently fused cells.

We fully agree with their interpretation. In our Mbnl1/2 knockdown model, AAV-mediated silencing occurs predominantly in myofibres and not in MuSCs, and our conclusions are based on this myofibre-restricted perturbation.

This restriction is supported by several independent lines of evidence:

- Promoter specificity

  The SPc5.12 promoter used in our AAV constructs is muscle-specific and differentiation-dependent. It is largely inactive in quiescent or proliferating MuSCs and becomes transcriptionally active primarily in post-mitotic myotubes and mature myofibre nuclei. Therefore, expression of the shmiR cassette in resting MuSCs is not expected.

- Limited AAV transduction of MuSCs

  AAV serotype 2/9 has been shown to transduce MuSCs very inefficiently (<1–2%), even at high intramuscular or systemic doses (Boutin et al., Nat Commun 2010; Kimura et al., Mol Ther 2014; Arnett et al., Mol Ther Nucleic Acids 2014), making a substantial direct effect on MuSCs unlikely.

- snRNA-seq validation

  Our single-nucleus RNA-seq analysis provides direct evidence for myofibre-restricted knockdown. A reduction in Mbnl1 transcripts was detected in myonuclei (Type IIx/IIa and IIb fibres), whereas no downregulation was observed in MuSCs (Supplementary Fig. S3A).

Taken together, these data indicate that Mbnl depletion in our model occurs primarily in myofibres, leading to secondary effects on MuSC behaviour, including activation and fusion. We therefore do not interpret our results as reflecting a cell-autonomous loss of Mbnl in MuSCs, but rather as demonstrating that MBNL dysfunction in mature myofibres is sufficient to trigger MuSC activation and myonuclear accretion.

In human DM1, the pathogenic CTG repeat expansion in *DMPK* is present in both myofibres and MuSCs, resulting in MBNL loss of function in both compartments. Accordingly, our mouse model does not aim to fully recapitulate all aspects of DM1, but instead allows us to isolate the contribution of myofibre-intrinsic MBNL loss to MuSC activation and altered myonuclear dynamics *in vivo*. We thank the reviewer for this important point and fully agree that alternative interpretations must be explicitly acknowledged.

We have therefore revised the text from:
"This work, however, establishes that myonuclear maturation is impaired or stalled after fusion, a mechanism that could contribute to DM1 pathology."
to:
"Together, these findings indicate that MuSC fusion contributes to myonuclear heterogeneity in DM1 muscle and gives rise to centrally located myonuclei with altered transcriptional states and high expression of the toxic *DMPK* transcript."

In the discussion section:
"Although our findings support the idea of impaired or delayed acquisition of a mature myonuclear program following fusion, we cannot completely rule out an alternative possibility that the observed signature reflects a sustained influx of recently fused MuSC-derived nuclei that have not yet fully completed maturation."

8. Lines 124-126: what are the data showing fibre splitting?

We agree that fibre splitting is not clearly visible in the representative image presented in Figure 1. To provide clearer evidence, we have added transmission electron microscopy (TEM) images in the Supplementary Data, which more clearly illustrate fibre splitting (Supplementary Fig. S1G).

9. Lines 164-165: when was snRNAseq performed after Mbnl1 KD? Should be added to results or figure legend.

snRNA-seq was performed 10 weeks after AAV-mediated Mbnl knockdown. This information has been added to the Results section for clarity.

10. Lines 165-167: Fig 2A should highlight the distinct myonuclear population.

Figure 2A has been updated to better highlight the distinct myonuclear populations, improving clarity and visual interpretation.

11. Lines 180-182: It is good practice to test transcriptional states with other available datasets. However, wouldn't it be more appropriate to do this with a dataset from adult regeneration (mdx, denervation, or muscle overload) instead of postnatal development?

The original idea of the overlap was to show the "developmental" nature of the signatures of the *Meg3*$^{+}$ and *Ankrd1*$^{+}$ myonuclei after *Mbnl* KD. However, we agree that it could be interesting to also do this with adult models. Therefore, we compared our model to a denervation model (Li et al., 2022) and see an increase *Ankrd1* population but not the *Meg3*. Similarly, we reanalyzed data from an overload model and saw an increase in the *Ankrd1* expression and a slight increase in *Meg3*$^{+}$ nuclei (Sun et al., 2024).

12. Figure 2: Full marker gene lists are needed to evaluate the emerging myonuclear populations in DM1. These are not provided. How similar are the Meg3+ and Ankrd1+ myonuclei during postnatal development and DM1? Are they simply reversion to immaturity that is normal or immaturity that is maladaptive? Perhaps integration of the datasets would be helpful or a venn diagram of marker gene lists.

a. I am also not sure that Ankrd1 and Meg3 signifies 'immaturity'. It is difficult without seeing all genes enriched in those populations. Also, multiple papers in the field show Ankrd1 is associated with times of muscle growth and remodeling.

We have now provided the full marker gene lists for the *Meg3*$^{+}$ and *Ankrd1*$^{+}$ myonuclear subpopulations in a Supplementary Table (Supplementary Table T5-6), allowing direct evaluation of their transcriptional signatures.

We agree with the reviewer that *Meg3* and *Ankrd1* expression should not be interpreted solely as markers of immaturity. Accordingly, we have revised the Results and Discussion sections to clarify that these populations likely reflect a stress-responsive and remodeling-associated myonuclear state rather than developmental immaturity per se.

As asked by referee #2, we have added the following three comparisions:

1) We have overlapped the snRNA-seq data from the human and mouse data, where we can largely appreciate that the Ankrd1+ cells broadly overlap with the totality of the DM myonuclei, including but not limited to Cluster X (Supplementary Fig. 15).

2) We compared the list of marker genes that distiguish the Meg+ myonuclei, the Ankrd1+ myonuclei and the Cluster X myonuclei from the DM patients and found similar percentage of overlap between the two popualtions (32/236 Meg+ ~13% 205/1666 Ankrd1 ~12%). 3) The list of genes which overlap between the two different human and mouse clusters are listed in Supplementary Table 9).

Likely, Cluster X represents an analogous state to the Ankrd1+ nuclei in the mouse model, as Ankrd1+ nuclei have been seen in other damage myofiber scenarios, including muscle overloading (Sun et al Nat Commun 2024).

13. For snRNAseq in figure 2, how many samples were analyzed? There should be some validation of the data in independent animals. The authors could do RNAscope for marker genes. This would also help determine if these populations are centrally or peripherally located, which could indicate whether a signature is derived from MuSCs.

For the snRNA-seq analysis in Figure 2, two biological samples per condition were analysed. We chose to address the cellular origin of the signatures using snRNA-seq on Pax7-DTA mice rather than RNAscopes. Indeed, histological analysis revealed that the number of peripheral myonuclei remains unchanged after MuSC ablation, whereas the number of central nuclei decreased. To determine whether Meg3 and Ankrd1 reactivation depends on MuSC-derived nuclei, we performed snRNA-seq on muscles from Pax7-depleted mice 10 weeks after Mbnl KD (Pax7^{DTA} Mbnl KD) and compared them to Mbnl KD in Pax7^{CreERT2} control mice (Pax7^{CreERT2} Mbnl KD). We first confirmed using this approach that DTA efficiently reduces the Pax7^{+} cell population at 85%. Notably, Pax7 depletion resulted in a partial reduction of the *Meg3*$^{+}$/*Ankrd1*$^{+}$

nuclear subpopulation (Figure 3G-H ; Supplementary Fig S6B). This suggests that although Pax7$^+$ cells contribute, they are not the only source of these new populations. From this experiment, it is not possible to determine if the reactivation occurs in the pre-existing nuclei, or if they are derived from other non-Pax7 derived fusing cells. However, in the original experiment, we observed that both *Meg3*$^+$ and *Ankrd1*$^+$ populations express MBNL1, suggesting that they indeed derive from a cell type that was not targeted by the AAV virus for knockdown.

14. There is a disconnect between the snRNAseq analysis and the MuSC accretion phenotype in the Mbnl 1/2 KD muscles. The snRNAseq is focused on characterizing the transcriptional changes in the immature myonuclear population (which the authors incorrectly say is MuSC-derived) but nothing on the stem cell compartment. Also, are activated myoblasts found in this dataset, which would be expected.

We thank the reviewer for this comment. In the MuSC compartment, the number of nuclei captured in our snRNA-seq dataset was insufficient to draw robust conclusions, as snRNA-seq of whole muscle is dominated by myonuclei. Consistent with this limitation, only a small number of differentially expressed genes were identified between Pax7$^+$ MuSCs in control and Mbnl knockdown muscles. Nevertheless, although not statistically significant, we observed a modest increase in the proportion of nuclei expressing the proliferation marker Mki67 in the knockdown tibialis anterior compared to control. To further address the reviewer's concern, we have now included dot plots (Supplementary Fig. S3B) showing increased expression of canonical myogenic activation markers, including *Myod* and *Myog*, within myonuclear populations. In addition, Gene Ontology analysis of genes upregulated in myonuclei revealed enrichment for pathways associated with muscle activation and remodeling (Supplementary Fig. S3C–D).

In response to the reviewer's concern that we "incorrectly say [the immature myonuclear population] is MuSC-derived," we sought to directly test MuSC contribution to this transcriptional program in vivo. To this end, we performed snRNA-seq following Pax7^{DTA}–mediated MuSC depletion in the Mbnl knockdown background. Pax7$^+$ cells were reduced by approximately 85%, confirming efficient but incomplete ablation. Notably, this resulted in a partial reduction of the *Meg3*$^+$/*Ankrd1*$^+$ myonuclear population, indicating that MuSC-derived nuclei contribute to this program but are not its sole source.

The incomplete reduction is consistent with residual MuSC fusion due to incomplete ablation and/or additional mechanisms, including fusion of non–Pax7-derived progenitors or transcriptional reactivation within pre-existing myonuclei. Consistent with this interpretation, the *Meg3*$^+$/*Ankrd1*$^+$ cluster shows partial transcriptional overlap with markers associated with interstitial progenitor populations such as pericytes and fibro–adipogenic progenitors. We have revised the Results and Discussion to clarify this mixed-origin interpretation and to avoid implying that this population is exclusively MuSC-derived.

a. There is also a lack of connection between the human and mouse transcriptomic data, especially within the immature myonuclear populations and stem cells. A better characterization will make the story more compelling. It is not intuitive to the reader that what is observed in the mouse is also present in human samples. For instance, authors characterized p21 in human samples but p21 is not even mentioned in the mouse model, which makes the rationale weak or not clear. There are many examples of these discrepancies.

Indeed, there are differences between the mouse model and human DM1 samples, both in muscle physiology and transcriptomic responses. Factors such as the timing and extent of MBNL depletion, fibre type composition, and the presence of expanded CTG in *DMPK* transcripts in human cells can all influence gene expression. However, the main argument behind the manuscript is not to draw a direct link between populations seen in the mouse and the human data but rather to compare conserved biological processes, including $Pax7^+$ MuSCs activation and the emergence of myonuclei with immature or remodeling-associated transcriptional programs. As detailed in our response to Comment 12, we performed additional cross-species analyses that support the existence of analogous transcriptional states between mouse and human myonuclei, while acknowledging species-specific differences.

Minor comments:

1) Line 308-309 authors argue that IIx fibers are reduced in human biopsies, but data is highly variable, so they should either increase the n or remove the statement.

We thank the reviewer for this important observation. We agree that, given the small cohort size and the donor-to-donor variability observed in the snRNA-seq and histological data, the evidence is insufficient to support a definitive statement that type IIx fibres are reduced in human DM1 biopsies.

Accordingly, we have removed the claim of a reduction in IIx fibres from the main text and reframed this observation as a qualitative trend rather than a statistically supported conclusion. The revised text now explicitly acknowledges inter-individual variability and avoids overinterpretation of fibre-type abundance changes in the human samples.

2) Graph Figure 4E is hard to distinguish the categories. A color bar graph might be better.

We changed figure 4E (and figure 4F, now figure 5) to color bar graphs to make it easier to distinguish the different categories.

Reviewer #4 (Remarks to the Author):

I co-reviewed this manuscript with one of the reviewers who provided the listed reports. This is part of the Nature Communications initiative to facilitate training in peer review and to provide appropriate recognition for Early Career Researchers who co-review manuscripts.

Reviewer's Comments:

*Line numbers refer to the revised manuscript with all changes accepted.

Reviewer #1 (Remarks to the Author)

I appreciate that the authors sincerely addressed most of my concerns. The new data indicate a limited contribution of MuSCs to the increased total number of myonuclei (response to original comment #1). In addition, the increased number of myonuclei derived from muscle satellite cells does not appear to contribute to the pathology or functional deficits observed in DM1 model mice. The authors speculate about the contribution of non-myogenic cells; however, these cells have not been identified. In addition, the authors did not address my original comment #6; therefore, it remains unclear why MuSCs begin to proliferate in DM1.

We agree that understanding why MuSCs become activated in the context of MBNL depletion is a key mechanistic question that requires further clarification.
In our study, the snRNA-seq analysis of MuSCs revealed only minimal transcriptional changes, arguing against a strong cell-autonomous disruption of quiescence pathways within MuSCs themselves. These data indicate that MuSC activation is primarily driven by extrinsic signals originating from MBNL-deficient myofibres.
Consistent with this interpretation, bulk RNA-seq revealed increased expression of multiple growth factors (Hgf, Fgf6, Areg, Gdf11, Gdf15), together with upregulation of extracellular matrix components and remodeling enzymes (Col1a1, Fn1, Mmp3, Postn), supporting a coordinated remodelling of the muscle niche and MuSC activation. In addition, we have now performed CellChat analysis on the snRNA-seq data, which revealed an overall increase in ligand–receptor interactions targeting MuSCs in the Mbnl KD TA compared to control conditions (added as Supplementary Figure S3E). These interactions originated not only from type IIx and IIb myofibers but also from FAPs, and involved multiple signaling axes, including growth factor signaling (FGF, EGF, BMP/TGF-β), Notch pathway activity, and extracellular matrix–mediated interactions (collagen/laminin–CD44/integrins), suggesting enhanced signaling inputs to MuSCs and consistent with their activation upon MBNL depletion.
Added (lines 180-185): " ...and revealed increased expression of multiple growth factors (*Hgf*, *Fgf6*, *Areg*, *Gdf11*, *Gdf15*), concomitant with upregulation of extracellular matrix components and remodeling enzymes (*Col1a1*, *Fn1*, *Mmp3*, *Postn*; Supplementary Table T2). Together, these changes reflect a shift in the muscle microenvironment that is favourable for MuSC activation and suggest that MBNL depletion in post-mitotic fibres promotes the activation and proliferation of MuSCs and/or other cell types present in the muscle."

Added (lines 215-220): "Further CellChat analysis revealed an overall increase in ligand–receptor interactions toward MuSCs in the Mbnl KD condition, originating from type IIx and IIb myofibres and FAPs, and involving growth factor signalling (*Fgf*, *Egf*, *Bmp/Tgf-β*), Notch pathway activity, and extracellular matrix–related interactions (collagen/laminin–CD44/integrins) supporting MuSCs activation upon Mbnl depletion (Supplementary Fig. S3E)."

We also agree with the reviewer that MuSC-derived nuclei do not appear to significantly contribute to the functional deficits observed in our model. We have now highlighted this point more clearly in the revised manuscript, emphasizing that MuSC fusion represents a remodelling response whose functional consequences remain to be defined.

Added: "Although MuSC activation is clearly observed in response to MBNL depletion, the underlying trigger remains unresolved. Our findings support a model in which MBNL-deficient myofibres alter the muscle microenvironment, thereby promoting MuSC activation and fusion in the absence of overt degeneration. Potential contributing factors may include altered mechanical signalling, metabolic stress, or changes in paracrine signalling; however, the precise molecular drivers remain to be identified." (line 622 ff).

Finally, our results suggest that the fusion of non-myogenic cells, such as FAPs or pericytes, may also contribute to the observed phenotype. Although these cells were not identified, as this was beyond the scope of the present study, this hypothesis has been discussed (line 674 ff).

Lines 203-: myonuclear transcriptional profiles revealed decreased expression of Mbnl1

--- Fig. 2A shows the expression of MBNL1/2 in KD mice. To what extent is MBNL1/2 expression decreased? If the majority of myonuclei express MBNL1/2, this would suggest that many myofibers have died and were subsequently replaced by satellite cell–derived regenerated myofibers.

In contrast, the results in Fig. S5C indicate that even after 10 weeks, the expression levels of MBNL1/2 remain very low. It is therefore important to clearly indicate how many myonuclei with reduced MBNL1/2 levels (i.e., pre-existing myonuclei) remain, as this would support the notion that myofiber necrosis is not overt.

In our snRNA-seq data, Mbnl1 and Mbnl2 transcripts remain detectable in a large proportion of myonuclei in Mbnl-KD muscles. However, this reflects transcript detection sensitivity and the intrinsic limitations of nuclear RNA profiling rather than preservation of normal expression levels. Importantly, shRNA-mediated knockdown primarily acts in the cytoplasm to reduce target mRNA levels, while snRNAseq captures nuclear RNA, which can include residual and unprocessed transcripts that are not representative of steady-state cytoplasmic mRNA abundance or protein levels.
This interpretation is further supported by our orthogonal analyses: Western blotting demonstrates an approximate 50% reduction in MBNL1 protein levels, and RNA-seq analysis shows widespread splicing abnormalities consistent with MBNL loss of function. Together, these data indicate that the majority of myonuclei in Mbnl-KD muscles are functionally MBNL-deficient.
We do not observe evidence for a substantial population of "replacement" myonuclei with restored MBNL expression that would be expected following extensive degeneration–regeneration cycles. This is further supported by the absence of changes in total myofibre number and lack of histological signs of necrosis.
To clarify this point, we have revised the *Results* section to explicitly describe the global shift in Mbnl1/2 expression levels across myonuclei and to avoid any ambiguity arising from detection-based visualization in UMAP plots: "myonuclear transcriptional profiles revealed decreased expression of *Mbnl1*"

was now changed to "transcriptional profiles revealed a global reduction in *Mbnl1* expression across myonuclear populations, without evidence for a distinct subpopulation retaining control-like expression levels–in contrast to other cell types–…". (line 209 ff).

Lines 234-: Their presence may reflect the incorporation of activated MuSC nuclei into existing fibres following a process of regeneration and/or myonuclear accretion. ---'regeneration' should be replaced with 'postnatal development'.

We agree that the term "regeneration" may be misleading in this context, as it could imply degeneration–regeneration cycles. However, we believe that "postnatal development" is not appropriate for adult muscle. We have therefore revised the text to refer more precisely to "myonuclear accretion," which better reflects MuSC fusion into existing fibres without implying prior degeneration. It now reads: "Their presence may reflect the incorporation of activated MuSC nuclei into existing fibres through myonuclear accretion, rather than classical regeneration following degeneration." (lines 246-248).

Lines 237-

---Why did the authors use a denervation model? Do MuSCs become activated and enter a proliferative state in denervated muscles? The authors should clearly describe the aim of this experiment. If the possibility of neurodegeneration occurring in MBNL-KD is being considered, this should be clearly stated. In fact, MyoD and Myog expression is increased in myofibers following denervation (Cell. 2010 Oct 1;143(1):35-45. doi:10.1016/j.cell.2010.09.004.).

The denervation model was not intended to imply that neurodegeneration contributes to the phenotype observed in Mbnl-KD muscles. Rather, it was included as a reference perturbation model to assess whether the transcriptional signatures identified in Mbnl-KD myonuclei (e.g., *Ankrd1*$^+$ and *Meg3*$^+$ populations) are specific to MBNL loss or represent a more general response to muscle stress or remodeling.
Denervation is a well-established condition that induces profound transcriptional remodeling in myofibres, including activation of genes such as *Myod1* and *Myog*, as noted by the reviewer. Our intention was therefore to determine whether similar transcriptional programs are activated in an independent, non-MBNL-related perturbation.
We agree that denervation can lead to MuSC activation and proliferation and involves complex changes including altered neuromuscular signaling and muscle remodeling. However, we emphasize that our use of this model is comparative rather than mechanistic, and does not imply that denervation or neurodegeneration occurs in the Mbnl-KD model.
Importantly, we do not observe evidence of denervation in Mbnl KD TA muscles, consistent with previous reports in MBNL1-KO mouse models (Lee et al., 2013; Frison-Roche et al., 2024).
We have now revised the manuscript to clarify this point and explicitly state the purpose of the denervation experiment. The paragraph "To test this hypothesis, we further compared our model with snRNA-seq data from muscle 14 days after sciatic nerve transection[48]. Despite expected differences in the myonuclei composition, we observed a similar population of *Ankrd1*+ myonuclei in the denervated animals that was absent in the controls. Finally, we re-analysed data from sorted myonuclei after overload[49] and observed a marked increase in *Ankrd1* expression and the appearance of the *Meg3*+ population, similar to what was observed in Mbnl KD."
now reads:

"To assess whether these transcriptional states are specific to MBNL depletion or reflect a more general response to muscle perturbation, we compared our data with published snRNA-seq datasets from muscle 14 days after sciatic nerve transection[48], a well-established model of myofibre stress and transcriptional remodelling. Despite expected differences in myonuclear composition, we observed a similar population of *Ankrd1*⁺ myonuclei in denervated muscle that was absent in controls. Finally, we re-analysed data from sorted myonuclei after overload[49] and observed a marked increase in *Ankrd1* expression and the appearance of a *Meg3*⁺ population, similar to that observed in Mbnl-KD muscle." (line 248 ff).

Lines 241; Collectively, this suggests that these populations are in response of muscle to physiological perturbations such as injury or overload (Supplementary Fig. S4C,D).

---Related to the above point, did the authors use denervation as an injury model?

We agree that the previous wording could be misinterpreted as implying that denervation was used as an injury model or that similar mechanisms operate in the Mbnl-KD condition. This was not our intention. The denervation dataset was used as a comparative reference to assess whether similar transcriptional states occur in other muscle perturbation contexts. We have revised the text to remove the term "injury" and to clarify that these comparisons are descriptive and do not imply that denervation or neurodegeneration contributes to the Mbnl-KD phenotype.

To clarify that, we changed the text to "Collectively, this suggests that these populations represent a common myonuclear response to muscle perturbation and remodelling, rather than a process specific to MBNL depletion, and do not imply that denervation or injury contributes to the Mbnl-KD phenotype" (line 256 ff).

SFig 5H (related to original comment #3 and #4):

---There are a significant number of EdU-positive myonuclei despite the absence of MuSCs. Is it possible that pre-existing myonuclei incorporate EdU? While the authors mention potential non-myogenic contributions, the possible contribution of pre-existing myonuclei is not discussed.

The authors state that MBNL1 is expressed in $Meg3^+$ or $Ankrd1^+$ myonuclei. Is this result described in the main text? Are there no MBNL1-negative myonuclei ing the $Meg3^+$ or $Ankrd1^+$ myonuclei? Similarly, was MBNL expression detected in the EdU-positive myonuclei observed in Pax7-DTA mice? In addition, is it possible that pre-existing myonuclei underwent division, leading to a reversion of MBNL1/2 expression to baseline levels?

Regarding the presence of EdU-positive myonuclei in Pax7-DTA mice, we agree that this observation requires careful interpretation. While EdU incorporation is commonly used to mark newly incorporated nuclei derived from proliferating MuSCs, it is important to note that EdU labels DNA synthesis rather than cell division per se. Therefore, in the absence of MuSCs, EdU incorporation may reflect alternative processes.
Importantly, mature myonuclei are widely considered post-mitotic and are not thought to undergo cell division under physiological or pathological conditions. Thus, we consider it unlikely that EdU-positive myonuclei arise from division of pre-existing myonuclei. However, EdU incorporation in these nuclei could reflect DNA repair, endoreduplication,

or contribution from non–Pax7-derived progenitor populations that are not ablated in the Pax7-DTA model.

We agree that the possibility of EdU incorporation in pre-existing myonuclei should be acknowledged. We have now expanded the Discussion to explicitly include this alternative interpretation and to avoid over-attributing EdU-positive nuclei exclusively to MuSC-derived fusion.

Added lines 601 ff): "To further refine the interpretation of these findings, particularly with respect to EdU-based lineage tracing, it is important to consider potential alternative sources of EdU incorporation. While EdU incorporation is commonly interpreted as evidence of newly fused MuSC-derived nuclei, it is important to note that EdU labels DNA synthesis rather than cell division. In the absence of MuSCs, EdU-positive myonuclei could reflect alternative processes such as DNA repair, endoreduplication, or contribution from non–Pax7-derived progenitor populations. Given that mature myonuclei are considered post-mitotic, direct division of pre-existing myonuclei is unlikely, but cannot be excluded." (line 599 ff)

Regarding MBNL1 expression in *Meg3*$^{+}$ and *Ankrd1*$^{+}$ myonuclei, we confirm that Mbnl1 transcripts are detectable in these populations in our snRNA-seq dataset. However, as noted above, shRNA-mediated knockdown primarily acts in the cytoplasm to reduce target mRNA levels. For this reason, nuclear Mbnl1 transcript detection cannot be used to infer whether these nuclei originate from pre-existing MBNL1-negative or MBNL1-positive myonuclear pools. Accordingly, we consider it too speculative to interpret MBNL1 nuclear transcript levels in these subpopulations in terms of nuclear provenance or prior expression history.

Similarly, we did not specifically assess MBNL expression in EdU-positive myonuclei at the single-cell level. We agree that such analysis would be informative but not using the current snRNAseq approach; however, it is beyond the scope of the current study.

Reviewer #2 (Remarks to the Author):

I would like to thank the authors for the thorough and careful work carried out during this revision. They have addressed my previous comments with clarity and added substantial new analyses and explanations where needed. The manuscript has improved considerably in both rigor and presentation. I appreciate the effort invested in refining the study and in providing detailed, constructive responses throughout.

We thank the reviewer for their positive evaluation of our revised manuscript and for their constructive comments throughout the review process. We appreciate that the revisions and additional analyses have addressed the concerns raised.

Reviewer #3 (Remarks to the Author)

The authors were responsive to critiques and the manuscript is improved. There are altered interpretations and the main piece of new data is the snRNAseq data on Pax7-DTA; Mbnl KD muscle, which clarifies the claim that fusion of MuSCs is only partially impacting gene expression in myofibers. While I am supportive of publication, I would strongly encourage the authors to highlight, perhaps in abstract, final paragraph of intro, and/or discussion, that there is not an obvious effect on the muscle phenotype (myotonia, muscle function) when MuSCs are deleted. I think this is an important result when much of the discussion is about these transient nuclear states of unknown significance, and mentioning no effect on the muscle phenotype does not detract from the main point of the paper that Mbnl loss in myofibers induces fusion.

> We thank the reviewer for this constructive suggestion and for their support of the manuscript. We agree that the absence of a measurable improvement in muscle phenotype following MuSC ablation is an important finding that provides critical context for interpreting the role of MuSC-mediated fusion and the emergence of transcriptionally distinct myonuclear states.
> In response, we have revised the manuscript to explicitly highlight this point in the Abstract, the final paragraph of the Introduction, the Results section, and the Discussion. We now clearly state that, although MuSC ablation reduces central nucleation and partially affects myonuclear composition, it does not significantly alter key functional parameters, including myotonia and muscle force.
>
> *Abstract*: "MuSC lineage tracing and ablation demonstrate that MuSC fusion contributes to central nucleation, and that MuSC deletion has no effect on the muscle phenotype, such as myotonia and muscle strength, in this model." (lines 35-37)
>
> *Results*: "However, no significant changes in functional readouts including myotonia and specific force were observed following MuSC deletion and reduced central nucleation." (lines 310-311)
>
> *Discussion*: "However, this intervention did not result in measurable improvements in muscle function, including myotonia or force generation, indicating that MuSC-mediated fusion is not a primary driver of the functional phenotype." (lines 595-598)
>
> *and* "Taken together, these findings suggest that MuSC-mediated fusion represents a prominent histopathological feature contributing to myonuclear remodelling and heterogeneity, but has a limited direct impact on overt muscle dysfunction in this model, which is likely primarily driven by intrinsic defects in MBNL-deficient myofibres." (lines 618-621)

I also have one minor concern about the below excerpt in the discussion:

"Our findings therefore provide, to our knowledge, the first direct in vivo evidence in a mammalian disease model, and supportive evidence in human DM1 muscle, that MuSC fusion occurs under pathological conditions in muscle."

This sentence reads that this paper is the first to show MuSC fusion under pathological conditions. That is certainly not true as MuSC fusion is known to occur in other muscle pathologies such as DMD.

> We changed this sentence to "Our findings therefore provide a direct *in vivo* evidence in a mammalian model of DM1, supported by observations in human DM1 muscle, that MuSC

fusion contributes to myonuclear accretion under pathological conditions in DM1 muscle." (lines 565-567)

Reviewer #4 (Remarks to the Author):

I co-reviewed this manuscript with one of the reviewers who provided the listed reports. This is part of the Nature Communications initiative to facilitate training in peer review and to provide appropriate recognition for Early Career Researchers who co-review manuscripts.
